# First comprehensive identification of cardiac proteins with putative increased O-GlcNAc levels during pressure overload hypertrophy

**DOI:** 10.1371/journal.pone.0276285

**Published:** 2022-10-26

**Authors:** Wei Zhong Zhu, Teresa Palazzo, Mowei Zhou, Dolena Ledee, Heather M. Olson, Ljiljana Paša-Tolić, Aaron K. Olson

**Affiliations:** 1 Seattle Children’s Research Institute, Seattle, Washington, United States of America; 2 Environmental Molecular Sciences Division, Pacific Northwest National Laboratories, Richland, Washington, United States of America; 3 Division of Cardiology, Department of Pediatrics, University of Washington, Seattle, Washington, United States of America; Rutgers New Jersey Medical School, UNITED STATES

## Abstract

Protein posttranslational modifications (PTMs) by O-GlcNAc globally rise during pressure-overload hypertrophy (POH). However, a major knowledge gap exists on the specific proteins undergoing changes in O-GlcNAc levels during POH primarily because this PTM is low abundance and easily lost during standard mass spectrometry (MS) conditions used for protein identification. Methodologies have emerged to enrich samples for O-GlcNAcylated proteins prior to MS analysis. Accordingly, our goal was to identify the specific proteins undergoing changes in O-GlcNAc levels during POH. We used C57/Bl6 mice subjected to Sham or transverse aortic constriction (TAC) to create POH. From the hearts, we labelled the O-GlcNAc moiety with tetramethylrhodamine azide (TAMRA) before sample enrichment by TAMRA immunoprecipitation (IP). We used LC-MS/MS to identify and quantify the captured putative O-GlcNAcylated proteins. We identified a total of 700 putative O-GlcNAcylated proteins in Sham and POH. Two hundred thirty-three of these proteins had significantly increased enrichment in POH over Sham suggesting higher O-GlcNAc levels whereas no proteins were significantly decreased by POH. We examined two MS identified metabolic enzymes, CPT1B and the PDH complex, to validate by immunoprecipitation. We corroborated increased O-GlcNAc levels during POH for CPT1B and the PDH complex. Enzyme activity assays suggests higher O-GlcNAcylation increases CPT1 activity and decreases PDH activity during POH. In summary, we generated the first comprehensive list of proteins with putative changes in O-GlcNAc levels during POH. Our results demonstrate the large number of potential proteins and cellular processes affected by O-GlcNAc and serve as a guide for testing specific O-GlcNAc-regulated mechanisms during POH.

## Introduction

Posttranslational modifications (PTM) of serine/threonine protein residues by O-linked β-N-acetylglucosamine (O-GlcNAc) are a dynamic and reversible process affecting hundreds of proteins [1–3]. Diverging from most PTMs, protein O-GlcNAcylation is regulated by a single enzyme for attachment (O-GlcNAc transferase, OGT), and a single enzyme for removal (O-GlcNAcase, OGA). Thus, protein O-GlcNAc modifications typically increase or decrease in a coordinated manner in response to a stimulus.

Human and animal models have repeatedly shown increased global protein O-GlcNAc levels in the heart during pressure overload hypertrophy (POH) and heart failure [4–10]. Yet, there remains a major knowledge deficit on O-GlcNAc’s mechanisms of action partially because only a small number of specific proteins with altered O-GlcNAc levels have been identified during these pathologies [11–13]. The fragility of the O-GlcNAc moiety, which is lost during standard mass spectrometry methodologies used to derive peptide sequence information and/or assign the modification site during proteomic studies, has hampered identification of O-GlcNAc changes on specific proteins [14]. The O-GlcNAc moiety is also low abundance, so sample enrichment is required to detect these modified proteins [15]. Methodologies have emerged to enrich O-GlcNAcylated proteins (or peptides) allowing for their identification and potential quantitation using standard LC-MS/MS techniques [15–17]. However, these approaches have not been used to identify changes in O-GlcNAc levels on cardiac proteins during POH [17].

O-GlcNAc’s myocardial effects during POH are presumably mediated by changes from baseline in O-GlcNAc levels on specific proteins. Accordingly, our goal was to create a comprehensive list of proteins undergoing changes in O-GlcNAc levels during POH. We used the established model of POH from transverse aortic constriction (TAC) to increase global protein O-GlcNAc levels. From these hearts, we first modified and tagged the O-GlcNAc moiety with tetramethylrhodamine azide (TAMRA) and then enriched for O-GlcNAc modified proteins using a TAMRA immunoprecipitation [17]. Subsequently, we identified and quantified the captured proteins via LC-MS/MS. A limitation with our approach, however, is that it does not determine the specific O-GlcNAc modified peptides within the proteins which would have provided further confirmation that the enrichment was due to O-GlcNAc modification. Thus, we refer to the TAMRA-enriched proteins as putative O-GlcNAcylated proteins. Nevertheless, the discovery of proteins with purported changes in O-GlcNAc levels during POH is an important step towards comprehensively understanding O-GlcNAc’s mechanisms of action during this pathological process. Follow-up studies should confirm the O-GlcNAc status of these candidate proteins while evaluating the functional consequences from these changes.

## Materials and methods

### Ethics statement

This investigation conforms to the Guide for the Care and Use of Laboratory Animals published by the National Institute of Health (NIH Pub. No. 85–23, revised 1996) and were reviewed and approved by the Office of Animal Care at Seattle Children’s Research Institute. All materials used in this study are available commercially from the indicated vendors.

### Mice

Pressure overload affects cardiac function and hypertrophic growth differently in males and females, so we performed these experiments in a single gender [18, 19]. Male C57BL/6J mice from the Jackson Laboratory (Bar Harbor, ME) between the ages of 3 and 5 months were used throughout. All mice were allowed free access to water and Teklad #7964 chow (Envigo, East Millstone, NJ, USA). Teklad ¼ inch corncob bedding was utilized in the cages (Envigo, East Millstone, NJ, USA). The mice were on a 12-hour light cycle.

### Experimental set-up

For the proteomics studies, we compared hearts 1-week post-surgery from either TAC (POH) or Sham (Sham). We chose this duration after surgery because our prior study showed total protein O-GlcNAc levels are substantially increased by approximately 70% at this time and this is also a period of active hypertrophic growth and remodeling [10].

POH often alters total levels along with other posttranslational modifications like phosphorylation for many proteins compared to Sham, which could affect protein activities independently of O-GlcNAc status. Therefore, to better isolate and assess O-GlcNAc’s specific effects on protein functions during POH, we used a separate model previously established in our lab to alter O-GlcNAc levels during POH only [10]. Male mice 4-weeks post-TAC were given intraperitoneal injections of the OGA inhibitor thiamet-g (TMG) for 2 weeks to increase total O-GlcNAc levels or vehicle (V). The groups for these experiments are TMG-TAC and V-TAC and were previously detailed [10]. We showed total protein O-GlcNAc levels were significantly increased by about 4-fold in TMG-TAC versus V-TAC male mice (10) [10]. Cardiac function and morphometrics were also unchanged in male TMG-TAC versus V-TAC [10].

### Surgery

We performed TAC or Sham surgery as previously described in our lab [9, 10, 20]. Briefly, mice were initially anesthetized with 3% isoflurane in 100% O2 at a flow of 1 LPM and then maintained with 1.5% isoflurane for the duration of the surgery. A midline sternotomy was performed to expose the aorta. The aorta was constricted with 7–0 silk suture tied against a 22-gauge blunt needle. Sham mice underwent similar surgery but did not have the suture tightened around the aorta. For pain control, mice received intraperitoneal buprenorphine (0.05–0.1 mg/kg IP) starting pre-operatively and continuing approximately every 12 hours for two and a half days.

### Echocardiograms

We performed echocardiograms as previously described in our lab [9, 20–22] to measure cardiac function and the degree of aortic constriction from the surgery. Briefly, mice were initially sedated with 3% isoflurane in O2 at a flow of 1 LPM and placed in a supine position at which time the isoflurane is reduced to 1.0% administered via a small nose cone. ECG leads were placed for simultaneous ECG monitoring during image acquisition. Mice were maintained at a temperature of greater than 37.0° C throughout the echocardiogram. Echocardiographic images were performed with a Vevo 2100 machine using a MS250 or MS550 transducers (VisualSonics, Inc, Toronto, Canada). M-Mode measurements at the midpapillary level of the left ventricle (LV) were performed at end-diastole (EDD) and end-systole (ESD) to determine LV function via the fractional shortening [(LVEDD-LVESD)/LVEDD * 100] in a parasternal short axis mode for at least three heart beats. We measured the pulse wave Doppler velocity across the aortic constriction site for a non-invasive estimate of the peak instantaneous pressure gradient across the constriction site as calculated by the simplified Bernoulli equation (Δ pressure gradient = 4*velocity^2^). The echocardiogram reader was blinded to treatment. We previously found that isoflurane anesthesia during echocardiograms increases myocardial total protein O-GlcNAc levels for several hours after the procedure (unpublished data), so the echocardiograms were always performed one day prior to sacrifice.

### Thiamet-G (TMG)

We used the selective OGA inhibitor TMG (Cayman Chemical Company, Ann Arbor, MI) at 20 mg/kg/day once daily for two weeks intraperitoneal to increase protein O-GlcNAc levels as previously described in our lab [10]. TMG was prepared in dimethyl sulfoxide (approximately 20 microliters) and the V-TAC received an equivalent volume of the vehicle only.

### Western blot for global protein O-GlcNAc levels

Global protein O-GlcNAc levels were determined by western blots using the RL2 antibody from Abcam (ab2739, Cambridge, MA, USA) on freshly isolated protein lysates as previously described from our lab [9, 10, 20]. Briefly, protein was isolated with a lysis buffer containing 10 mM Tris (pH = 7.6), 150 mM NaCl, 0.5% deoxycholate, 0.1% SDS, 1% NP-40 containing phosphatase inhibitor (ThermoFisher Scientific, Waltham, MA, USA) plus the OGA inhibitors PUGNAc 20 μM (Toronto Research Chemical, North York, ON, Canada) and Thiamet-G 1μM (Adooq Bioscience, Irvine, CA, USA). Fifty micrograms of total protein extract from mouse heart tissue was separated electrophoretically and transferred to PVDF membrane. The RL2 antibody is raised in mice and the secondary anti-mouse antibody can react with IgG from the tissue resulting in a non-specific band at either 55 kD (from IgG heavy chain) or 25 kD (from IgG light chain). To allow for unobstructed detection of O-GlcNAc bands in the 55 kD region, we only used a light chain specific secondary antibody (Peroxidase AffiniPure goat anti-mouse IgG, light chain specific; Jackson ImmunoResearch Laboratories, Inc., West Grove, PA) for our O-GlcNAc immunoblots and excluded the 25 kD band during quantification. Total (global) O-GlcNAc levels were measured by densitometry of the entire western blot lane and normalized to the protein staining of the entire lane by Thermo Scientific Pierce Reversible Protein Stain Kit for PVDF Membranes (Thermo Scientific, Rockford, IL, USA).

### Enzymatic labeling of O-GlcNAc-modified proteins for sample enrichment

O-GlcNAc modified proteins were labeled using Click-iT^™^ O-GlcNAc Enzymatic Labeling System (Thermo Fisher Scientific, Waltham, MA) according to manufacturer’s instructions. Briefly, protein was extracted in RIPA lysis buffer plus phosphatase inhibitor (Thermo Fisher Scientific, Waltham, MA), the OGA inhibitors PUGNAc 20 μmol/L (Toronto Research Chemical) and Thiamet-G 1 μmol/L (Adooq Bioscience, Irvine CA). According to the manufacturer’s instructions, we used only the soluble fraction from our protein lysate to avoid non-specific Click-iT^™^ labeling of N-glycosylated cell-surface proteins. 200 μg of total protein was precipitated using chloroform/methanol to remove the detergents followed by centrifugation at 14,000g for 5 minutes at 4°C. The interface layer containing the protein precipitate was washed twice with methanol. The pellet was dried for 5 minutes in a standard fume hood. The dried pellet was resuspended in 40μl of SDS (1%) and HEPES buffer (20 nM, pH 7.9), boiled at 90°C for 5 minutes, vortexed briefly, and allowed to cool on ice for 3 minutes. Labeling buffer was then added followed by UDP-GalNAz and Gal-T1. After overnight incubation at 4°C, the GalNAz-labeled O-GlcNAc-modified protein mixture was precipitated using the chloroform/methanol precipitation method and resuspended in 50μl of buffer containing SDS (1%) and Tris (50mM, pH 8). The sample was subsequently labeled with the TAMRA-alkyne dye using the Click-It^™^ TAMRA Glycoprotein Detection Kit (Thermo Fisher Scientific, Waltham, MA). The mixture was vortexed for 5 seconds after the addition of each component according to manufacturer’s instructions. The mixture was then rotated for 1 hour at 4°C for the conversion of the azide group to a stable triazole conjugate (TAMRA). DTT (25 mM) was added to stop the reaction. Labeled samples were precipitated using methanol/chloroform/water, brought up to a concentration of 2 mg/mL in 1% SDS and HEPES buffer (20 nM, pH 7.9), plus Complete^™^ protease inhibitors.

### Immunoprecipitation of TAMRA-labeled putative O-GlcNAcylated proteins

TAMRA labeled proteins were immunoprecipitated using Pierce MS-Compatible Magnetic IP Kit (Thermo Fisher Scientific, Waltham, MA) according to manufacturer’s instructions. Briefly, the labeled protein solution was precleared against washed protein A/G magnetic beads (20 μL/200 μg of protein) at 4°C for 1 h. After bead separation, the supernatant was collected and incubated with an anti-TAMRA antibody (Thermo Fisher Scientific, Waltham, MA) at 10 μg/200 μg of protein at 4°C overnight. The samples were then added to pre-washed protein A/G magnetic beads (25 μL) for 1 hour. The beads were collected and washed three times with IP-MS Wash Buffer. After washing, the beads were mixed with 100 μl elution buffer for 10 min. The supernatant was collected and dried for MS analysis.

### Mass spectrometry

From the same hearts, we prepared and ran TAMRA-enriched samples for assessing putative O-GlcNAc protein levels along with unenriched samples for a comparison to global protein levels. The samples were reduced with 5 mM dithiothreitol at 37°C for 1 h, and alkylated with 10 mM iodoacetamide in the dark at 25°C for 45 min. Then 100 mM Tris HCl was added. Samples were first digested with LysC (FUJIFILM Wako Chemicals, Richmond, VA) at enzyme to substrate ratio of 1:50 at 25°C for 2h, followed by trypsin (Promega Corporation Madison, WI) digestion overnight (c.a. 14 h) at 25°C with enzyme to substrate ratio of 1:50. Digested peptides with TAMRA enrichment were vialed at 0.1 μg/μL in 0.1% formic acid for liquid chromatography mass spectrometry (LCMS). A Waters NanoAcquity was operated in direct injection mode. Mobile phase A (MPA) was 0.1% formic acid in 97:3 water: acetonitrile, and mobile phase B (MPB) was 0.1% formic acid in 10:90 water: acetonitrile. LC column dimension was 20 cm × 75 μm i.d. with 1.9 μm ReproSil C18 packing prepared in house. 5 μL of peptides were loaded at 2% MPB for 30 min. Separation was performed by a gradient of 6% to 30% MPB over 85 min, followed by 10 min ramp to 60%. Flow rate was constant at 0.2 μL/min and column temperature was at 50°C. Mass spectra were collected using a Thermo Fisher Scientific Orbitrap Lumos (Waltham, MA) with data-dependent acquisition. MS resolution was 60k for MS1 and 30k for MS2. Alternating HCD (charge state 2–10) and ETD (charge state 3–10) were used for MS2. Data were processed with PEAKS Studio (10.0 build 2190129) with label free quantitation. Mouse proteome FASTA was accessed from UniProt, and common contamination proteins were appended. Dynamic modifications included protein N-terminal acetyl, methionine oxidation, carbamidomethylation, phosphorylation, O-HexNAc, and O-HexNAc with TAMRA label (only for TAMRA labeled samples). Mass error tolerance was 10 ppm for precursor, and 0.02 Da for fragments. False discovery rate filter was 1% with decoy-fusion option in PEAKS Studio. The protein abundances (total areas) were log2 transformed and normalized to median in Perseus [23]. Missing values were imputed from normal distribution. Raw data, FASTA, PEAKS output tables, and normalized results are available in massive.ucsd.edu (Accession: MSV000088797).

### Bioinformatics analysis

To determine whether the putative O-GlcNAcylated proteins in our study have previous citations similarly identifying them to be O-GlcNAcylated in vertebrates (humans, bovine, rats, or mice), we used the O-GlcNAc Database version 1.2 from the Olivier-Van Stichelen Lab at the Medical College of Wisconsin and noted the findings in S1 Table [24]. We used the gene ontology suite, DAVID Bioinformatics Resources 6.8 [25, 26] and KEGG (Kyoto Encyclopedia of Genes and Genomes) database [27] to assign molecular function (MF) and biological processes to acquire information on the proteins demonstrating significantly higher putative O-GlcNAc levels during POH.

### Immunoprecipitation (IP) to confirm O-GlcNAc levels on proteins of interest

Two metabolic enzymes identified as having increased putative O-GlcNAc levels with POH were chosen for further evaluation namely the pyruvate dehydrogenase (PDH) complex and carnitine O-palmitoyltransferase 1, muscle isoform (CPT1B). We used the PDH Rodent Immunocapture Kit (Abcam, Waltham, MA) to examine the subunits comprising this complex in POH versus Sham. However, due to product supply constraints and delays, we assessed O-GlcNAcylation of PDH in V-TAC versus TMG-TAC via a different method discussed in the next paragraph. Briefly, frozen heart tissues were lysed with 1X Extraction Buffer with a protease inhibitor cocktail and the OGA inhibitors PUGNAc 20 μmol/L (Toronto Research Chemical, North York, ON, Canada) and Thiamet-G 1 μmol/L (Adooq Bioscience, Irvine, CA). Solubilized homogenate was immunocaptured by mixing with PDH antibodies coupled agarose beads for 3 hours at 4 C in a tube rotator. The beads were collected by centrifugation for 2 minutes at 300×g and washed three times. The immunoprecipitated proteins were eluted with 40-μl SDS Elution Buffer. The samples were separated by SDS-PAGE and transferred to onto a polyvinylidene fluoride membrane. The membrane was subsequently stained for total protein with Pierce^™^ Reversible Protein Stain Kit (Thermo Fisher, Waltham, MA). The membranes were probed with Anti-O-Linked N-Acetylglucosamine antibody (anti-RL2, Novus Biologicals, Littleton, CO). The immunoblots were visualized with enhanced chemiluminescence using the chemiDoc-It^™^ imaging system (Analytik Jenna USA LLC). O-GlcNAc levels on the PDH proteins were normalized to protein staining in the same molecular weight region.

To assess changes in O-GlcNAc levels for CPT1B and the PDH complex subunits (TMG-TAC versus V-TAC only), we performed the immunoprecipitation following the instructions detailed in the Pierce Co-Immunoprecipitation Kit (#26149, Thermo Fisher Scientific, Waltham, MA). Briefly, the tissue lysate was precleared against washed protein A/G magnetic beads (20 μL/200 μg of protein) at 4°C for 1 h. After bead separation, the supernatant was collected and incubated with an anti-RL2 antibody (Novus Biologicals, Littleton, CO) at 10 μg/200 μg of protein at room temperature for 1 hour. The samples were then added to pre-washed protein A/G magnetic beads (25 μL) for 1 h. The beads were collected and washed three times with IP-MS Wash Buffer. After washing, the immunoprecipitated proteins were recovered by boiling in 50 μl 1X SDS buffer. The samples were separated by SDS-PAGE and transferred onto a polyvinylidene fluoride membrane. The membranes were probed with CPT1B antibody (Proteintech, Rosemont, IL) or the PDH antibody cocktail (Thermo Fisher Scientific, Waltham, MA). The immunoblots were visualized with enhanced chemiluminescence using the chemiDoc-It^™^ imaging system (Analytik Jenna USA LLC). We normalized the input protein levels to protein staining in the molecular weight region of interest. The IP fraction was normalized to input protein concentration as determined by the BCA assay (Thermo Fisher Scientific, Waltham, MA).

### CPT1 enzyme activity assay

CPT1 enzyme activity was determined through monitoring thiolytic cleavage of palmitoyl-CoA. The rate of the thiolytic cleavage was measured spectrophotometrically at 412 nm as described by Bieber et al. [28]. In brief, 20 mg of heart tissue were lysed and homogenized in ice-cold buffer containing 0.25 M sucrose, 1 mM EDTA, 0.1% ethanol, and protease inhibitors cocktail. Total protein was quantified by BCA assay (Thermo Fisher Scientific, Waltham, MA). The enzymatic activity was measured in 175 μl reaction buffer (116 mM Tris-HCl, 2.5 mM EDTA, 2 mM 5,5’-dithio-bis-(2-nitrobenzoic acid) (DTNB), pH 8.0) and 10 μg lysates in a final volume of 188 μl by adding homogenization buffer. The samples were then incubated at 30°C for 5 min to eliminate reactive thiol groups and the resulting background absorbance was measured. After preincubation, 10 μl palmitoyl-CoA (2 mM prepared in double distilled water) and 2 μl L-carnitine solution (1.2 mM in 1 M Tris-HCl, pH 8.0) were added to the reaction mixtures. Kinetic reads were collected at 2 minutes intervals for 30 min. In a parallel experiment, 0.2% Triton X-100 was added to inactivate CPT1. The CPT1 activity was calculated by subtracting the activity with Triton X-100 from total activity. Activity was defined as nmol CoA-SH released/min/mg protein. Calculations were made assuming a molar extinction coefficient of 13,600 M^−1^ cm^−1^ at 412 nm for the thiol reagent DTNB.

### PDH enzyme activity assay

PDH enzyme activity was measured according to the manufacturer’s instructions for the enzyme activity assay kit (K679-100, BioVision Inc., Milpitas, CA). Briefly, heart lysate was prepared with PDH assay buffer plus protease inhibitors (78440, Thermo Fisher, Waltham. MA). 20 μg/50 μL of lysate was loaded onto microplate wells. After adding 50 μL PDH reaction solution, PDH activity was determined by monitoring the change in absorbance at 450 nm over 60 min at 37°C. PDH activity was calculated by determining the slopes of the generated curves between the 10-min and 40-min time points where the increase in absorbance was linear. PDH Activity was defined as nmol NADH released/min/mg protein.

### Data visualization and statistical analysis

We preformed data visualization using GraphPad Prism version 8.3.3 (GraphPad Software, San Diego, CA, USA). All reported values are means ± standard error of the mean (SEM). All experiments except the TAMRA-enriched (O-GlcNAc) proteomics used an unpaired two-tailed student T-test. For the putative O-GlcNAc proteomics study, we and others have shown global protein O-GlcNAc levels significantly increase in POH versus Sham (see Results section). Accordingly, we *a priori* expected our results to show most proteins have higher log2 Fold levels towards POH. Of the 700 putative O-GlcNAcylated proteins identified in both POH and Sham (results shown later), only 8.2% had a log2 Fold level < 0 towards increased enrichment with Sham and none of these proteins reached statistical significance (p < 0.05 by an unpaired two-tailed student T-test). Thus, we were justified to use a one-tailed student T-test for the O-GlcNAc proteomics study only. Since our proteomics experiments were exploratory designed to generate hypotheses, we did not correct for multiple comparisons. Criterion for significance was set at p < 0.05.

## Results

### Echocardiographic and morphometric results during POH

**Fig 1:**
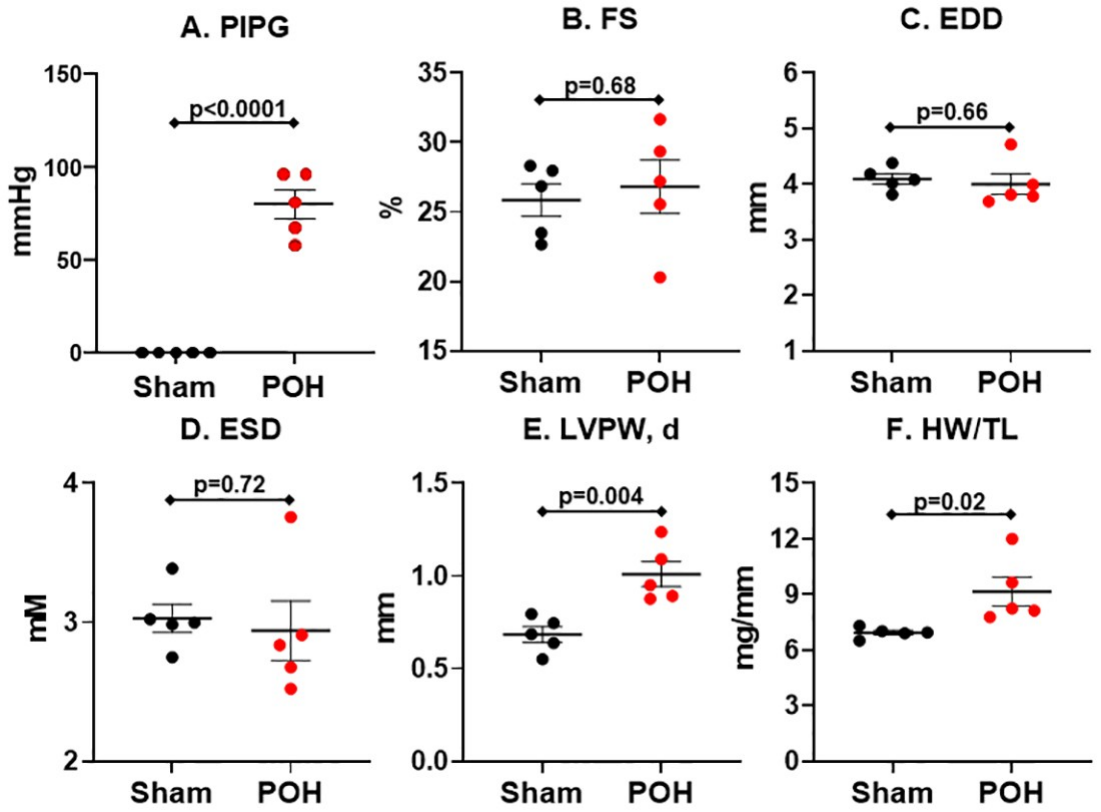
Echocardiogram and morphometrics in pressure-overload hypertrophy (POH) and sham (Sham) groups. Bars indicate mean± standard error of the mean. PIPG, peak instantaneous pressure gradient across the transverse aortic constriction site; FS, left ventricular fractional shortening; EDD, left ventricular end-diastolic diameter; ESD, left ventricular end-systolic diameter; LVPW, d, left ventricular posterior wall thickness in diastole; HW/TL, heart weight normalized to tibial length; mmHg, millimeter mercury; mM, millimeter; mG, milligram. N = 5 for all groups.

**Fig 2:**
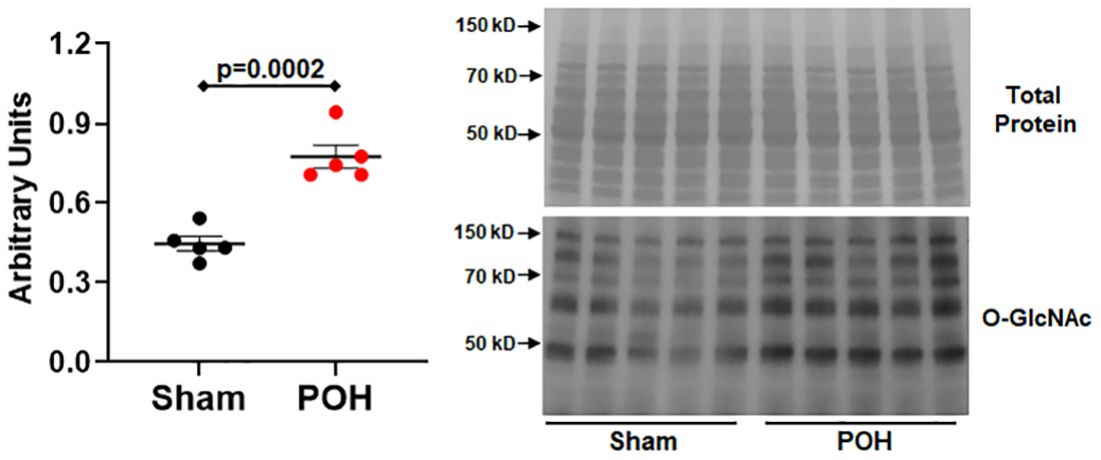
Global protein O-GlcNAc level in pressure-overload hypertrophy (POH) and sham (Sham) groups. Immunoblots are shown for global protein O-GlcNAc levels using the RL2 antibody normalized to protein staining shown above. Bars are mean± standard error of the mean. N = 5 per group.

We performed echocardiograms to show the cardiac parameters and the degree of aortic constriction in POH and Sham (Fig 1). The aortic constriction led to an average peak instantaneous pressure gradient of 79.6±7.7 mmHg across the constriction site indicating a high degree of stenosis from the constriction (Fig 1A). Left ventricular chamber size and systolic function was similar between the groups (Fig 1B–1D), but the left ventricular posterior wall thickness increased in POH providing echocardiographic evidence of hypertrophy (Fig 1E). Further, heart weight to tibial length (HW/TL) was significantly elevated in POH which also confirms the presence of cardiac hypertrophy (Fig 1F). We used immunoblots to confirm POH also increases global protein O-GlcNAc levels versus Sham in our experiments (Fig 2).

### Protein identification with analysis of biological processes and KEGG pathways

**Fig 3:**
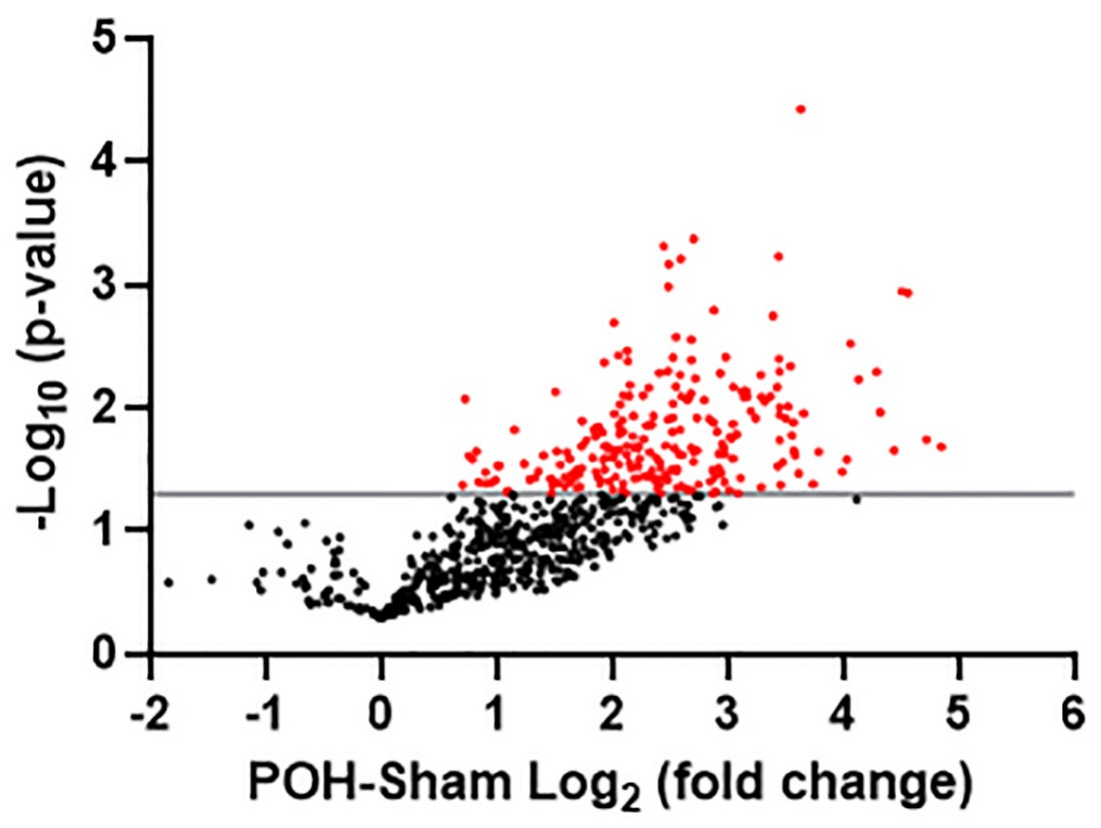
Volcano plot illustrating differentially enriched putative O-GlcNAcylated proteins in pressure-overload hypertrophy (POH) versus Sham. The −log_10_ p-value is plotted against the Log_2_ fold change in POH versus Sham. Each dot represents a single protein from the 700 total proteins identified in both POH and Sham. Red dots represent proteins with statistically significant enrichment changes (p<0.05) which is also demarcated by the gray horizontal line. n = 5 per group.

We discovered a total of 700 putative O-GlcNAcylated proteins present in both Sham and POH (Fig 3, S1 Table). Of these, 233 putative O-GlcNAcylated proteins were significantly increased in POH over Sham whereas no proteins were significantly decreased by POH (Fig 3, S1 and S2 Tables). After enrichment, proteins with putative higher O-GlcNAc levels could be due to (1) a greater percentage of O-GlcNAc modification on the protein, (2) a higher overall protein level with an unchanged percentage of O-GlcNAc modification, or (3) a combination of both circumstances. We were primarily interested in identifying proteins with a higher percentage of O-GlcNAc modification, so we further assessed global protein levels (modified and unmodified) in unenriched samples from the same hearts. Of the 233 proteins with putative increased O-GlcNAc levels in POH, 197 proteins were also identified in the global sample with 28 of these proteins having significantly higher global protein levels in POH. However, the fold change was higher for O-GlcNAc enrichment compared to global levels in POH for all but one protein (S3 Table). Accordingly, these results suggest that the significant changes in putative O-GlcNAc levels during POH are primarily from a higher percentage of O-GlcNAc modification on the proteins.

We used the DAVID software to analyze the 233 putative proteins with significantly increased O-GlcNAc levels (shown in S2 Table) to gain insight into the overrepresented biological processes and signaling pathways. A cutoff Benjamini corrected p-value of less than 0.05 was used in Tables 1 and 2. The biological processes and KEGG pathway analysis identified proteins predominantly involved in transcription, metabolic, and structural roles. S4 and S5 Tables include the list of putative proteins with increased O-GlcNAc levels within each significantly enriched biological process and KEGG pathway category.

**Table 1:** Biological processes with significant overexpression for putative O-GlcNAcylated protein changes during pressure overload hypertrophy (POH).

TERM	Number of proteins	Fold enrichment	p-value	Benjamini p-value
GO:0006412~translation	24	4.79	1.22E-09	1.80E-06
GO:0042407~cristae formation	7	43.08	5.54E-09	4.10E-06
GO:0055114~oxidation-reduction process	29	3.43	2.49E-08	1.23E-05
GO:0098609~cell-cell adhesion	13	5.50	4.51E-06	0.002
GO:0006754~ATP biosynthetic process	6	20.87	8.00E-06	0.002
GO:0046034~ATP metabolic process	7	14.00	9.36E-06	0.002
GO:0006096~glycolytic process	6	13.33	7.85E-05	0.02
GO:0006810~transport	42	1.84	1.30E-04	0.02

Note: Fold enrichment refers to the number expected versus the number of found putative O-GlcNAcylated proteins for a biological process.

**Table 2:** KEGG pathways with significant overexpression for putative O-GlcNAcylated protein changes during pressure overload hypertrophy (POH).

TERM	Number of proteins	Fold enrichment	p-value	Benjamini p-value
mmu01200:Carbon metabolism	17	7.88	2.81E-10	4.76E-08
mmu00190:Oxidative phosphorylation	18	6.96	5.18E-10	4.76E-08
mmu01130:Biosynthesis of antibiotics	20	5.03	1.12E-08	5.42E-07
mmu05012:Parkinson’s disease	17	6.14	1.18E-08	5.42E-07
mmu05016:Huntington’s disease	19	5.16	1.94E-08	7.13E-07
mmu03010:Ribosome	16	5.93	5.76E-08	1.77E-06
mmu05010:Alzheimer’s disease	17	5.17	1.37E-07	3.59E-06
mmu00010:Glycolysis / Gluconeogenesis	11	8.96	2.93E-07	6.73E-06
mmu01100:Metabolic pathways	47	1.99	1.96E-06	4.01E-05
mmu04932:Non-alcoholic fatty liver disease (NAFLD)	14	4.80	5.83E-06	1.07E-04
mmu00620:Pyruvate metabolism	7	9.65	7.04E-05	0.001177
mmu00280:Valine, leucine and isoleucine degradation	7	6.85	4.91E-04	0.007526
mmu00630:Glyoxylate and dicarboxylate metabolism	5	9.27	0.001848	0.026082
mmu00071:Fatty acid degradation	6	6.59	0.001984	0.026082
mmu00020:Citrate cycle (TCA cycle)	5	8.40	0.00268	0.03288
mmu04260:Cardiac muscle contraction	7	4.89	0.002882	0.033143

Note: Fold enrichment refers to the number expected versus the number of found putative O-GlcNAcylated proteins for a KEGG pathway.

### Immunoblotting and enzyme activities

**Fig 4:**
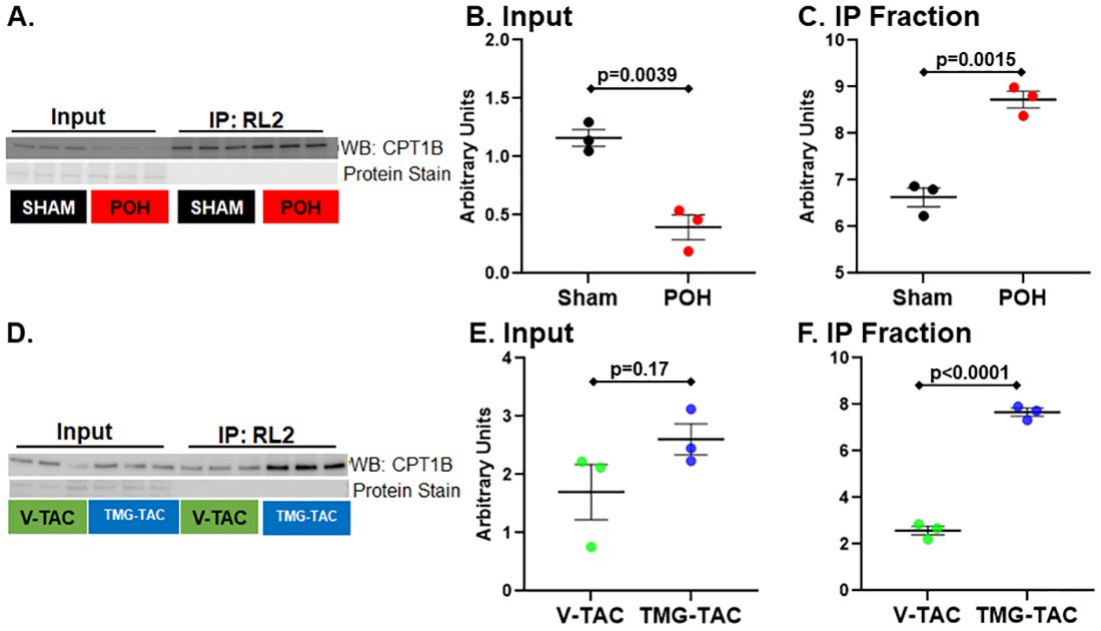
O-GlcNAc immunoprecipitation (IP) for carnitine palmitoyltransferase 1B (CPT1B). CPT1B western blots are show prior to (input) and after IP with the anti-O-GlcNAc antibody RL2 in POH and Sham (A) with quantification for input normalized to protein staining (B) and after IP (C). Western blots for experimental groups V-TAC and TMG-TAC are shown in D with quantification for input normalized to protein staining (E) and after IP (F). Bars indicate mean± standard error of the mean. N = 3 per group.

We chose two metabolic enzymes with putative increased O-GlcNAc levels from the proteomics results to validate O-GlcNAc status via a separate method along with an assessment on how the changes in O-GlcNAc levels during POH affect enzyme activity. CPT1B was chosen due to its function as a shuttle of long chain fatty acids into the mitochondria for beta-oxidation. First, CPT1B protein expression in whole cell tissue lysate, which we denote as the input levels for IP, was significantly higher in Sham versus POH (Fig 4A and 4B). Thus, overall protein levels of CPT1B fall with POH. Post- immunoprecipitation using the RL2 antibody to capture O-GlcNAcylated proteins showed CPT1B levels were greater in POH versus Sham confirming higher CPT1B O-GlcNAcylation during POH despite lower total levels (Fig 4A and 4C).

**Fig 5:**
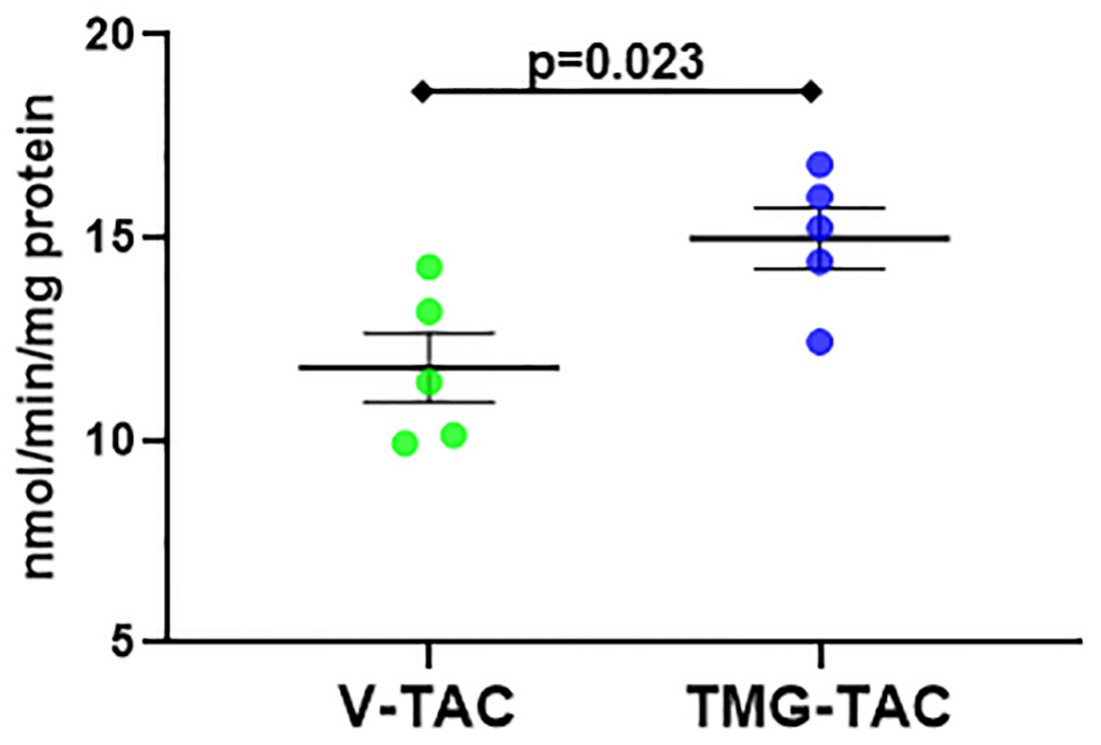
Quantification of enzyme activity for CPT1 in experimental groups V-TAC versus TMG-TAC. Activity is defined as nmol CoA-SH released/min/mg protein. Bars indicate mean± standard error of the mean. N = 5 per group.

We decided against using POH versus Sham to evaluate enzyme activities because POH often alters total protein levels (like for CPT1B) along with other posttranslational modifications, which would affect results separately from O-GlcNAc status. Thus, we compared V-TAC versus TMG-TAC to better isolate and assess O-GlcNAc’s specific effects on protein functions during POH [10]. Total CPT1B levels were similar in V-TAC versus TMG-TAC (denoted as the input for the IP), however CPT1B was greater in TMG-TAC after IP indicating higher CPT1B O-GlcNAcylation for TMG-TAC (Fig 4D–4F)). We found CPT1 enzyme activity was higher in TMG-TAC versus V-TAC suggesting O-GlcNAcylation increases CPT1 activity during POH (Fig 5).

**Fig 6:**
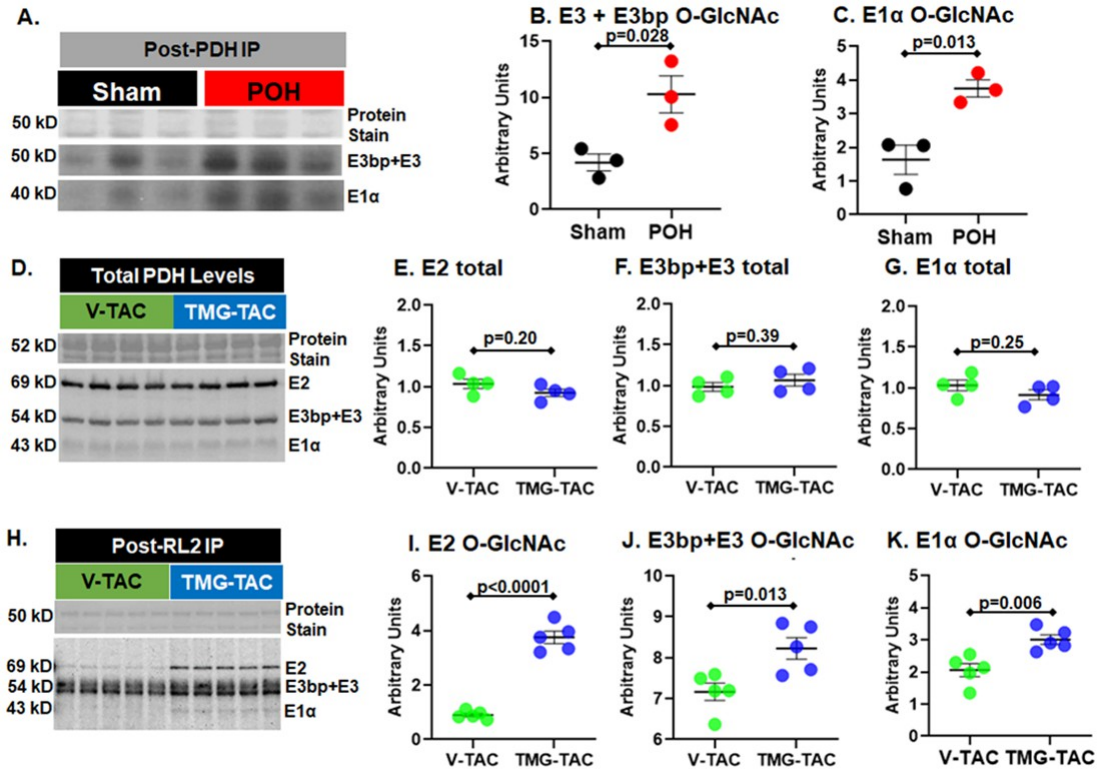
O-GlcNA levels on pyruvate dehydrogenase (PDH) subunits. A-C: O-GlcNAcylation of PDH subunits determined by PDH complex immunocapture followed by western blot with the anti-O-GlcNAc antibody RL2. (A) shows protein staining for the immunocaptured proteins and the RL2 bands at 50 kD and 40 kD. The bands at 50 kD correspond to the subunits E3 and E3 binding partner (E3bp) whereas 40 kD corresponds to the E1α subunit. (B) Quantification of E3 and E3bp normalized to protein staining. (C) Quantification of the E1α subunit normalized to protein staining. E2 band was not identified in this immunocapture. D-G: Total PDH protein levels via western blot in V-TAC and TMG-TAC. (D) Protein staining and western blot for PDH subunits. (E) Quantification of the E2 band normalized to protein staining. (F) Quantification of the E3bp+E3 bands normalized to protein staining. (G) Quantification of the E1α band normalized to protein staining. H-K: O-GlcNAcylation of the PDH subunits determined by O-GlcNAc immunoprecipitation (IP) with the RL2 antibody followed by western blot for PDH subunits. (H) Protein staining after IP with the RL2 antibody along with the post-IP western blot for the PDH subunits. (I) Quantification of the E2 band. (J) Quantification of the E3bp+E3 bands. (K) Quantification of the E1α band. Bars indicate mean± standard error of the mean. N = 3 per group for A-C, n = 4 per group for D-G, and n = 5 per group for H-K.

**Fig 7:**
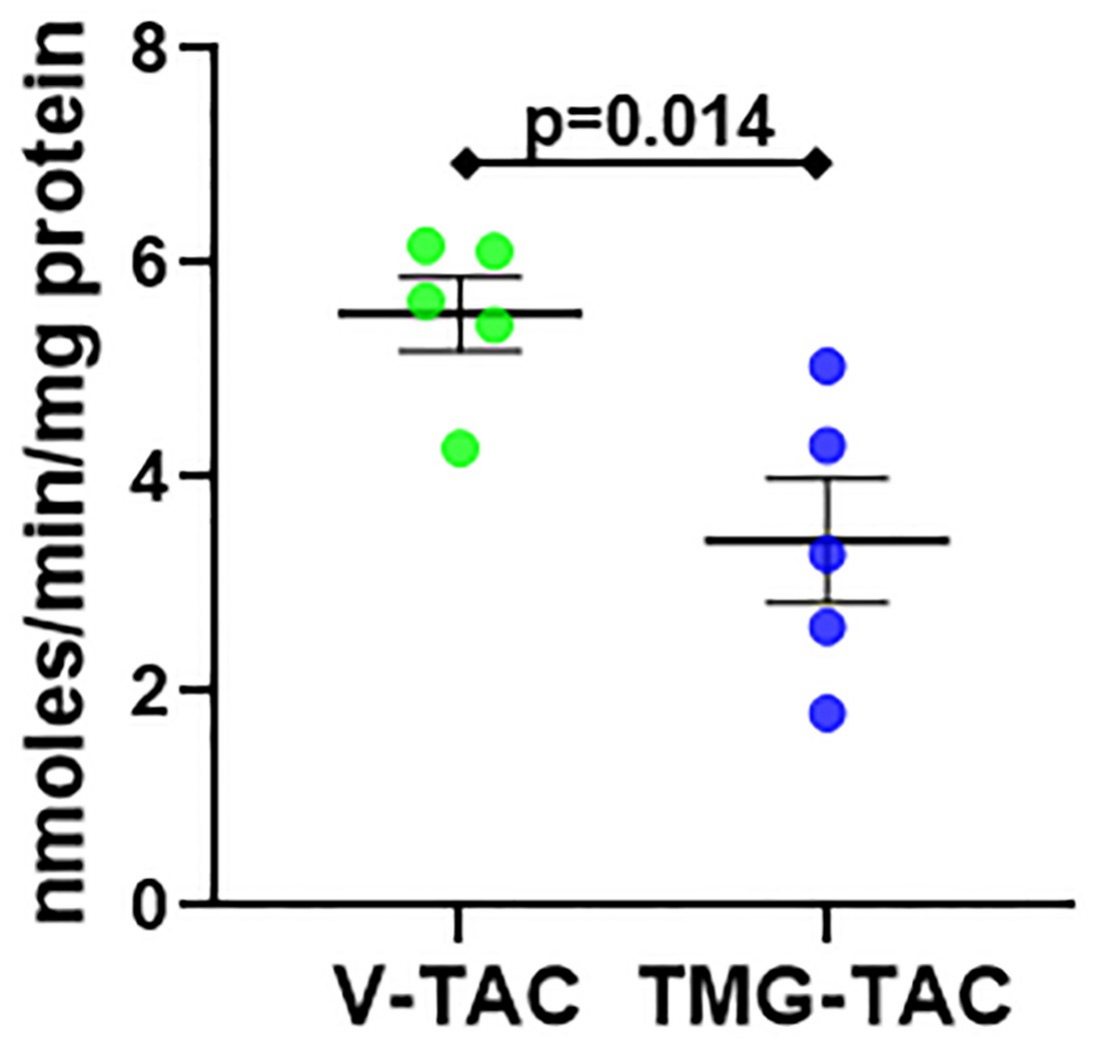
Quantification of enzyme activity for PDH in experimental groups V-TAC versus TMG-TAC. Bars indicate mean± standard error of the mean. N = 5 per group.

We next evaluated the PDH complex which is a metabolic enzyme converting pyruvate into acetyl-CoA for use in the citric acid cycle. The PDH complex is made up of the following subunits: pyruvate dehydrogenase (E1), dihydrolipoyl transacetylase (E2), dihydrolipoyl dehydrogenase (E3), and dihydrolipoyl dehydrogenase binding protein (E3BP). Our proteomics results showed putative increased O-GlcNAcylation of the PDH E2 and E3 subunits. We immunoprecipitated the PDH complex and then performed a RL2 western blot on the bound fraction to determine O-GlcNAc levels in Sham versus POH. We found increased O-GlcNAc levels on the immunoprecipitated PDH complexes in POH compared to Sham (Fig 6A–6C). We also assessed PDH subunit O-GlcNAcylation in the V-TAC versus TMG-TAC model following an initial RL2 IP. Total PDH protein levels were similar between V-TAC and TMG-TAC (Fig 6D–6G). However, we found higher PDH subunit levels after RL2 IP in TMG-TAC indicating higher O-GlcNAcylation (Fig 6H–6K). PDH enzyme activity was lower in TMG-TAC hearts suggesting that higher O-GlcNAcylation inhibits PDH activity during POH (Fig 7).

## Discussion

Our understanding of O-GlcNAc’s specific cellular effects during POH has been hampered by the absence of a large-scale list of proteins with changes in O-GlcNAc levels during this pathology. Utilizing Click-iT^™^ technology, we identified a total of 700 putative O-GlcNAcylated proteins in Sham and POH hearts, 233 of which exhibited significantly increased putative O-GlcNAcylation during POH. No proteins had significantly decreased O-GlcNAc levels during POH. Thus, we have generated the first comprehensive list of proteins with putative changes in O-GlcNAc levels during POH. Our results identify many proteins and cellular processes potentially affected by O-GlcNAc changes during POH. Future studies should advance our findings by confirming O-GlcNAc levels on specific proteins of interest followed by a determination of the functional effect on the O-GlcNAcylated proteins.

O-GlcNAcylation is often described as beneficial for acute stressors and detrimental with prolonged elevations [13, 29]. However, because of the large number of affected proteins, it is conceivable O-GlcNAc changes during POH are beneficial for some proteins/cellular functions while detrimental for others. Accordingly, we advocate future studies evaluate O-GlcNAc’s effects on specific proteins and cellular functions during POH. This knowledge could potentially optimize future translational therapies by identifying specific O-GlcNAcylated proteins to target rather than just global levels. Emerging technologies like aptamers or nanobodies could be developed to modify O-GlcNAc levels at specific protein sites [30]. It may also be possible to modify select groups of O-GlcNAcylated proteins by altering activity of the accessary proteins that target OGT to specific proteins [30].

### Putative O-GlcNAcylated proteins and cellular functions during POH

Previous studies indicate protein O-GlcNAcylation affects many cellular functions including cell cycle regulation, transcriptional and translational events, metabolism, mitochondria function, protein synthesis and quality control, autophagy, epigenetics, cellular signaling, contractile and cytoskeletal proteins, and calcium-handling [3, 31, 32]. In our study, proteins with putative increased O-GlcNAcylation were overrepresented for similar biological processes like translation, metabolism (ATP biosynthesis and metabolic processes, glycolytic processes), mitochondrial function (cristae formation) and cytoskeletal proteins (cell-cell adhesion). KEGG pathway analysis showed overrepresentation for multiple metabolic pathways, ribosome function and cardiac muscle contraction. During POH, ribosome function could affect hypertrophic growth and proteostasis whereas metabolic pathways and cardiac muscle contraction could directly alter cardiac function.

It is also noteworthy that many metabolic pathways including glycolysis, glucose/pyruvate oxidation, and fatty acid oxidation (FOA) were highly enriched for proteins with putative increased O-GlcNAcylation because this PTM is frequently described as a nutrient sensor that responds to metabolic changes [4, 8]. The hexosamine biosynthesis pathway (HBP) branches from glycolysis to produce UDP-GlcNAc, the moiety used by OGT for O-GlcNAc PTMs. OGT is sensitive to UDP-GlcNAc concentration making overall O-GlcNAc levels responsive to changes in HBP flux [33]. Thus, it is widely assumed that the elevated glycolytic rates occurring during POH or heart failure increase HBP flux to augment overall protein O-GlcNAc levels [8, 20]. However, we measured myocardial HBP flux for the first time and found that HBP flux does not change even through a 20-fold variation in glycolytic rates [34]. Our findings herein expand upon our prior work and raise the provocative possibility that O-GlcNAc alters metabolic enzymes during POH which could affect cardiac fuel metabolism. Whereas the unstressed heart relies mainly on fatty acid oxidation (FAO) as a fuel source, POH augments glucose utilization [35, 36]. This metabolic shift is important as changes in glucose metabolism directly affect hypertrophic growth and cardiac function [37–39]. Yet, strategies targeting fuel utilization for the citric acid cycle (CAC) to prevent heart failure in POH have not been realized partially because the mechanisms underlying these metabolic changes are incompletely known. Thus, it will be important the determine the contribution of O-GlcNAc to the metabolic alterations occurring during POH.

In this regard, we assessed two metabolic enzymes, the PDH complex and CPT1B, key enzymes for glucose/pyruvate oxidation and fatty acid metabolism respectively. We used immunoprecipitation to confirm the LC-MS/MS results suggesting increased O-GlcNAcylation for both enzymes during POH. Although O-GlcNAcylation increased, CPT1B total protein levels were substantially lower in POH versus Sham which would confound evaluating O-GlcNAc’s effect on enzyme activity between these groups. Therefore, we used the TMG-TAC and V-TAC hearts for both enzyme activity assays. Higher O-GlcNAcylation decreased PDH activity whereas it augmented CPT1B activity. O-GlcNAc’s effect on PDH could contribute to the finding that glucose oxidation often does not increase to the same extent as glycolysis during POH [35]. We speculate CPT1B O-GlcNAcylation could help preserve FAO in the face of alterations in other FAO enzymes occurring during POH [36]. However, we caution against making conclusions on O-GlcNAc’s metabolic effects during POH at this time because O-GlcNAc levels increased on multiple other enzymes involved in glucose and fatty acid metabolism; the citric acid cycle; and even mitochondrial function. Overall, our results show the clear need to comprehensively assess O-GlcNAc’s effect on myocardial substrate metabolism during POH.

### Limitations

Our approach relies on protein level enrichment and identification of primarily non-modified peptides derived from these enriched proteins using LC-MS/MS. Hence, it is probable that some of the proteins identified with our TAMRA enrichment protocol are not O-GlcNAcylated. First, Click-iT^™^ labels the terminal GlcNAc moiety on other glycosylation modifications beyond O-GlcNAcylation. Although we followed the manufacturer’s instructions to limit non-specific Click-iT^™^ labeling on N-glycosylated proteins, our raw spectra indicates that some nonspecific labeling occurred beyond O-GlcNAc. Further, some of our putative O-GlcNAcylated proteins may have bound to O-GlcNAcylated proteins during the TAMRA IP, nonspecifically bound the beads during IP, or represent other protein interferences from nonspecific labeling. Indeed, this is a common challenge when enriching low abundant proteins from a complex mixture using affinity purification methods. Proteins without other citations in the O-GlcNAc Database (indicated in S1 Table) are more likely to be false-positives. We advocate future studies confirm O-GlcNAc status of our reported proteins.

As discussed earlier, our methodology does not resolve site specificity for the O-GlcNAc modification. Site specific data would provide additional proof that an identified protein is O-GlcNAcylated along with determining whether an individual protein is modified on more than one site. Site specific information is also necessary for using gene editing techniques to remove O-GlcNAc sites while studying a protein of interest.

We only assessed male hearts at a single time during POH. It is likely that O-GlcNAcylation on specific proteins temporally vary based upon the duration of POH and/or the development of cardiac dysfunction/heart failure. It will also be important to determine whether female and male hearts have differences in O-GlcNAc protein specificity during POH.

Finally, a limitation with TMG is that it alters O-GlcNAc levels throughout the organism. Future studies assessing O-GlcNAc’s effects on specific proteins/cellular functions during POH should use cardiac specific methods to alter O-GlcNAc levels when possible.

## Conclusions

We generated the first comprehensive list of putative proteins with changes in O-GlcNAc levels during POH. Our list serves as a guide for testing specific O-GlcNAc-related mechanisms affecting the heart during POH.

## Supporting information

S1 Table Average O-GlcNAc levels for all identified putative proteins during pressure overload hypertrophy (POH) and Sham. Proteins were checked for additional O-GlcNAc citations in the O-GlcNAc Database v1.2 (www.oglcnac.org) from the Olivier-Van Stichelen Lab at the Medical College of Wisconsin. Proteins without previous citations are also highlighted in gray.
(DOCX)

S2 Table Proteins with significant changes in putative O-GlcNAc levels in pressure overload hypertrophy (POH) versus Sham. (DOCX)

S3 Table Proteins with significantly increased global and putative O-GlcNAc levels during pressure overload hypertrophy (POH). Log_2_ fold change of POH-Sham is shown for both global and O-GlcNAc protein levels.
(DOCX)

S4 Table Accessions for the putative proteins with increased O-GlcNAc levels during pressure-overload hypertrophy (POH) versus Sham for the biological processes with significant overexpression from Table 1. (DOCX)

S5 Table Accessions for the putative proteins with significantly increased O-GlcNAc levels during pressure-overload hypertrophy (POH) versus Sham for the KEGG pathways with significant overexpression from Table 2. (DOCX)

S1 Raw images Raw images for the western blots in this manuscript. (PDF)
