# Supplementary material for: First comprehensive identification of cardiac proteins with putative increased O-GlcNAc levels during pressure overload hypertrophy
Source: PLoS One. 2022 Oct 26;17(10):e0276285. doi: 10.1371/journal.pone.0276285 (PMC9605332; doi:10.1371/journal.pone.0276285)
Supplement: S1 Table — Proteins were checked for additional O-GlcNAc citations in the O-GlcNAc Database v1.2 (www.oglcnac.org) from the Olivier-Van Stichelen Lab at the Medical College of Wisconsin. Proteins without previous citations are also highlighted in gray. (DOCX) [file pone.0276285.s001.docx]

**S1 Table. Average O-GlcNAc levels for all identified putative proteins during pressure overload hypertrophy (POH) and Sham.** Proteins were checked for additional O-GlcNAc citations in the O-GlcNAc Database v1.2 (www.oglcnac.org) from the Olivier-Van Stichelen Lab at the Medical College of Wisconsin. Proteins without previous citations are also highlighted in gray.

| **Accession** | **Uniprot accession number** | **Average-**  **POH (n=5)** | **Average-sham (n=5)** | **Log2 Fold change**  **POH-Sham** | **p-value** | **Citations in the O-GlcNAc**  **Database v1.2?** |
| --- | --- | --- | --- | --- | --- | --- |
| RSSA_MOUSE | P14206 | -1.52 | -5.15 | 3.63 | 0.000037 | Yes |
| RANG_MOUSE | P34022 | -2.91 | -5.61 | 2.71 | 0.000424 | Yes |
| ERP44_MOUSE | Q9D1Q6 | -2.80 | -5.25 | 2.45 | 0.000487 | Yes |
| NHRF2_MOUSE | Q9JHL1 | -1.48 | -4.92 | 3.44 | 0.000586 | Yes |
| PTN11_MOUSE | P35235 | -2.53 | -5.12 | 2.59 | 0.000616 | Yes |
| ATPF1_MOUSE | Q811I0 | -2.75 | -5.24 | 2.49 | 0.000677 | Yes |
| SEPT8_MOUSE | Q8CHH9 | -2.89 | -5.38 | 2.49 | 0.001033 | Yes |
| IGJ_MOUSE | P01592 | -1.09 | -5.60 | 4.51 | 0.001124 | No |
| GRP78_MOUSE | P20029 | 2.30 | -2.26 | 4.56 | 0.001155 | Yes |
| TOM1_MOUSE | O88746 | -0.90 | -3.78 | 2.88 | 0.001606 | Yes |
| RL26_MOUSE | P61255 | -1.82 | -5.21 | 3.39 | 0.001779 | Yes |
| PAK2_MOUSE | Q8CIN4 | -2.24 | -4.25 | 2.02 | 0.002033 | Yes |
| PP14C_MOUSE | Q8R4S0 | -1.88 | -4.43 | 2.55 | 0.002649 | Yes |
| SRA1_MOUSE | Q80VJ2 | -2.70 | -5.39 | 2.69 | 0.002774 | Yes |
| G3BP1_MOUSE | P97855 | -0.96 | -5.02 | 4.06 | 0.002990 | Yes |
| GELS_MOUSE | P13020 | -3.25 | -5.38 | 2.13 | 0.003436 | Yes |
| NEXN_MOUSE | Q7TPW1 | -3.47 | -5.53 | 2.06 | 0.003737 | yes |
| CPZIP_MOUSE | Q3UZA1 | -2.04 | -5.02 | 2.99 | 0.003840 | Yes |
| GSTA4_MOUSE | P24472 | -2.64 | -5.17 | 2.53 | 0.003880 | No |
| CPT1B_MOUSE | Q924X2 | -1.24 | -4.68 | 3.45 | 0.004017 | Yes |
| MIC25_MOUSE | Q91VN4 | -2.31 | -5.00 | 2.69 | 0.004061 | Yes |
| HNRPQ_MOUSE | Q7TMK9 | -2.98 | -5.12 | 2.14 | 0.004176 | Yes |
| NDUS2_MOUSE | Q91WD5 | -2.99 | -4.92 | 1.93 | 0.004296 | Yes |
| PRDX6_MOUSE | O08709 | 0.00 | -3.55 | 3.54 | 0.004583 | Yes |
| SYP2L_MOUSE | Q8BWB1 | -1.82 | -5.27 | 3.45 | 0.005020 | Yes |
| CAH2_MOUSE | P00920 | -2.07 | -4.56 | 2.48 | 0.005035 | Yes |
| G6PI_MOUSE | P06745 | 1.70 | -2.59 | 4.29 | 0.005086 | Yes |
| AFG32_MOUSE | Q8JZQ2 | -2.95 | -5.36 | 2.41 | 0.005184 | Yes |
| MIC27_MOUSE | Q78IK4 | -1.11 | -4.05 | 2.94 | 0.005245 | Yes |
| IF5A1_MOUSE | P63242 | -2.02 | -5.30 | 3.29 | 0.005391 | Yes |
| RS18_MOUSE | P62270 | -2.57 | -5.16 | 2.59 | 0.005411 | Yes |
| TIM44_MOUSE | O35857 | -2.63 | -5.35 | 2.72 | 0.005733 | Yes |
| TRFE_MOUSE | Q921I1 | 0.00 | -4.14 | 4.13 | 0.005890 | Yes |
| TBA4A_MOUSE | P68368 | -2.67 | -4.82 | 2.15 | 0.006503 | Yes |
| G3BP2_MOUSE | P97379 | -2.43 | -4.99 | 2.55 | 0.006716 | Yes |
| FBN1_MOUSE | Q61554 | -1.48 | -4.91 | 3.43 | 0.006772 | Yes |
| GDIB_MOUSE | Q61598 | -2.39 | -5.44 | 3.05 | 0.006778 | Yes |
| MTCH2_MOUSE | Q791V5 | -2.56 | -4.88 | 2.32 | 0.006861 | Yes |
| CAV1_MOUSE | P49817 | -1.22 | -4.38 | 3.16 | 0.007277 | Yes |
| MYOZ2_MOUSE | Q9JJW5 | -0.72 | -3.84 | 3.12 | 0.007331 | No |
| MOES_MOUSE | P26041 | 2.51 | 1.01 | 1.51 | 0.007365 | Yes |
| DEST_MOUSE | Q9R0P5 | -2.19 | -4.87 | 2.68 | 0.007598 | Yes |
| GPSM1_MOUSE | Q6IR34 | -3.52 | -5.61 | 2.09 | 0.007880 | No |
| UB2L3_MOUSE | P68037 | -1.83 | -4.10 | 2.27 | 0.007916 | Yes |
| ITB1_MOUSE | P09055 | -2.82 | -4.97 | 2.15 | 0.007986 | Yes |
| ROA3_MOUSE | Q8BG05 | 0.06 | -3.31 | 3.37 | 0.008024 | Yes |
| RADI_MOUSE | P26043 | -3.04 | -5.64 | 2.60 | 0.008048 | Yes |
| MLIP_MOUSE | Q5FW52 | -2.13 | -5.30 | 3.17 | 0.008086 | Yes |
| CYB5_MOUSE | P56395 | -1.31 | -4.61 | 3.29 | 0.008112 | Yes |
| TGM2_MOUSE | P21981 | -1.69 | -4.83 | 3.14 | 0.008184 | Yes |
| PGAM1_MOUSE | Q9DBJ1 | -0.96 | -3.63 | 2.67 | 0.008304 | Yes |
| RL30_MOUSE | P62889 | 0.11 | -0.62 | 0.73 | 0.008471 | Yes |
| RAN_MOUSE | P62827 | -1.68 | -4.33 | 2.65 | 0.008549 | No |
| MIMIT_MOUSE | Q59J78 | -1.32 | -4.12 | 2.80 | 0.008659 | No |
| FKBP3_MOUSE | Q62446 | -2.04 | -5.36 | 3.32 | 0.008887 | Yes |
| SPTN1_MOUSE | P16546 | -2.88 | -5.41 | 2.53 | 0.009230 | Yes |
| PABP2_MOUSE | Q8CCS6 | -3.42 | -5.49 | 2.07 | 0.009390 | Yes |
| PCCA_MOUSE | Q91ZA3 | -1.34 | -4.87 | 3.52 | 0.009723 | Yes |
| DCTN2_MOUSE | Q99KJ8 | -1.87 | -5.32 | 3.45 | 0.009857 | Yes |
| EIF3B_MOUSE | Q8JZQ9 | -1.45 | -4.65 | 3.20 | 0.010636 | Yes |
| SFPQ_MOUSE | Q8VIJ6 | -1.03 | -5.35 | 4.32 | 0.010891 | Yes |
| NDUV1_MOUSE | Q91YT0 | 0.03 | -3.63 | 3.66 | 0.011055 | Yes |
| AK1A1_MOUSE | Q9JII6 | -2.75 | -4.77 | 2.02 | 0.011177 | Yes |
| ICAL_MOUSE | P51125 | -1.58 | -5.03 | 3.45 | 0.011343 | Yes |
| YBOX1_MOUSE | P62960 | -2.45 | -4.63 | 2.19 | 0.011552 | Yes |
| PRC2C_MOUSE | Q3TLH4 | -3.10 | -5.46 | 2.36 | 0.011628 | Yes |
| SGCA_MOUSE | P82350 | -3.09 | -5.62 | 2.53 | 0.012048 | No |
| COX5A_MOUSE | P12787 | 4.47 | 1.73 | 2.74 | 0.012066 | Yes |
| GRPE1_MOUSE | Q99LP6 | -1.71 | -5.22 | 3.51 | 0.012174 | Yes |
| L2HDH_MOUSE | Q91YP0 | -1.53 | -4.78 | 3.24 | 0.012217 | Yes |
| RS4X_MOUSE | P62702 | -2.26 | -5.10 | 2.84 | 0.012239 | Yes |
| TKT_MOUSE | P40142 | -2.71 | -5.20 | 2.48 | 0.012427 | Yes |
| PDLI1_MOUSE | O70400 | 1.97 | -0.12 | 2.09 | 0.012556 | Yes |
| RT02_MOUSE | Q924T2 | -2.94 | -4.68 | 1.74 | 0.012759 | Yes |
| STIP1_MOUSE | Q60864 | -1.19 | -4.76 | 3.57 | 0.013110 | Yes |
| HP1B3_MOUSE | Q3TEA8 | -2.80 | -5.67 | 2.88 | 0.013114 | Yes |
| TTHY_MOUSE | P07309 | -1.53 | -4.58 | 3.05 | 0.013560 | No |
| B2MG_MOUSE | P01887 | -1.43 | -3.77 | 2.34 | 0.013683 | Yes |
| RS7_MOUSE | P62082 | -2.80 | -4.86 | 2.06 | 0.013769 | Yes |
| EH1L1_MOUSE | Q99MS7 | -2.85 | -5.14 | 2.30 | 0.013895 | Yes |
| PGAM2_MOUSE | O70250 | 3.45 | 1.57 | 1.89 | 0.014374 | Yes |
| NOL3_MOUSE | Q9D1X0 | -3.25 | -5.12 | 1.87 | 0.014391 | No |
| ANXA7_MOUSE | Q07076 | -2.61 | -5.20 | 2.59 | 0.015013 | Yes |
| HSPB1_MOUSE | P14602 | 5.17 | 4.02 | 1.15 | 0.015054 | Yes |
| CTNA1_MOUSE | P26231 | -3.04 | -4.88 | 1.84 | 0.015091 | Yes |
| LASP1_MOUSE | Q61792 | -2.08 | -4.60 | 2.52 | 0.015634 | Yes |
| ALDH2_MOUSE | P47738 | 0.18 | -2.73 | 2.91 | 0.015639 | Yes |
| CAP1_MOUSE | P40124 | -2.58 | -4.68 | 2.10 | 0.015661 | Yes |
| ACADM_MOUSE | P45952 | 3.69 | 1.77 | 1.92 | 0.015739 | Yes |
| CYTC_MOUSE | P21460 | -3.86 | -5.92 | 2.06 | 0.016175 | No |
| CO3_MOUSE | P01027 | -2.58 | -5.66 | 3.08 | 0.016390 | Yes |
| RL12_MOUSE | P35979 | -1.82 | -5.38 | 3.56 | 0.016675 | Yes |
| LAMA2_MOUSE | Q60675 | -3.50 | -5.68 | 2.18 | 0.016960 | Yes |
| LC7L2_MOUSE | Q7TNC4 | -3.16 | -5.01 | 1.85 | 0.016983 | Yes |
| B2L13_MOUSE | P59017 | -2.38 | -5.41 | 3.03 | 0.017125 | Yes |
| GCAB_MOUSE | P01864 | -1.27 | -4.31 | 3.04 | 0.017133 | No |
| VIME_MOUSE | P20152 | 1.81 | -1.22 | 3.03 | 0.017561 | Yes |
| RT33_MOUSE | Q9D2R8 | -2.60 | -4.88 | 2.27 | 0.017899 | No |
| NDUB3_MOUSE | Q9CQZ6 | 1.00 | -3.72 | 4.72 | 0.018048 | No |
| CLIP1_MOUSE | Q922J3 | -2.26 | -5.71 | 3.45 | 0.018239 | Yes |
| TRI72_MOUSE | Q1XH17 | 0.99 | -0.78 | 1.77 | 0.018366 | No |
| ACTN2_MOUSE | Q9JI91 | -1.30 | -4.25 | 2.95 | 0.019728 | Yes |
| SNRPA_MOUSE | Q62189 | -1.72 | -4.19 | 2.46 | 0.020206 | Yes |
| NEBL_MOUSE | Q0II04 | -2.07 | -4.30 | 2.23 | 0.020220 | Yes |
| PLIN4_MOUSE | O88492 | -1.06 | -3.19 | 2.13 | 0.020328 | Yes |
| CLAP1_MOUSE | Q80TV8 | -3.11 | -4.84 | 1.73 | 0.020357 | Yes |
| THIL_MOUSE | Q8QZT1 | 4.20 | 2.24 | 1.96 | 0.020374 | Yes |
| AKAP2_MOUSE | O54931 | -1.72 | -3.65 | 1.94 | 0.020531 | Yes |
| QCR7_MOUSE | Q9D855 | 1.53 | -3.32 | 4.85 | 0.020701 | Yes |
| REEP5_MOUSE | Q60870 | -1.77 | -3.96 | 2.19 | 0.020951 | Yes |
| RL13A_MOUSE | P19253 | -2.62 | -5.30 | 2.68 | 0.020957 | Yes |
| DLRB1_MOUSE | P62627 | -2.45 | -5.41 | 2.97 | 0.021080 | No |
| NDUS8_MOUSE | Q8K3J1 | 0.80 | -1.74 | 2.54 | 0.021310 | Yes |
| PSMD9_MOUSE | Q9CR00 | -1.86 | -4.09 | 2.23 | 0.021361 | Yes |
| PALMD_MOUSE | Q9JHU2 | -2.71 | -4.78 | 2.06 | 0.021667 | No |
| AIFM1_MOUSE | Q9Z0X1 | 1.05 | -0.94 | 1.99 | 0.021870 | Yes |
| HSPB7_MOUSE | P35385 | -1.54 | -4.28 | 2.74 | 0.022053 | No |
| ROAA_MOUSE | Q99020 | -2.98 | -5.03 | 2.05 | 0.022069 | Yes |
| EF1D_MOUSE | P57776 | 1.36 | -3.09 | 4.44 | 0.022172 | Yes |
| PACN3_MOUSE | Q99JB8 | -1.52 | -3.54 | 2.02 | 0.022206 | Yes |
| 1433T_MOUSE | P68254 | -0.78 | -4.36 | 3.58 | 0.022250 | Yes |
| DLDH_MOUSE | O08749 | 2.76 | 1.24 | 1.52 | 0.022433 | Yes |
| COF1_MOUSE | P18760 | 0.24 | -0.58 | 0.82 | 0.022436 | Yes |
| MYH7_MOUSE | Q91Z83 | -1.82 | -4.52 | 2.70 | 0.022691 | Yes |
| MACD1_MOUSE | Q922B1 | -0.33 | -4.12 | 3.79 | 0.022738 | Yes |
| RS6_MOUSE | P62754 | -1.69 | -4.02 | 2.33 | 0.022869 | Yes |
| VAPB_MOUSE | Q9QY76 | -0.39 | -2.00 | 1.61 | 0.022997 | Yes |
| FBLN2_MOUSE | P37889 | -2.07 | -4.99 | 2.92 | 0.023528 | Yes |
| ALDOA_MOUSE | P05064 | 4.53 | 1.55 | 2.97 | 0.023540 | Yes |
| SODM_MOUSE | P09671 | -1.10 | -3.68 | 2.58 | 0.023723 | Yes |
| CHCH2_MOUSE | Q9D1L0 | -0.43 | -2.55 | 2.13 | 0.023896 | No |
| CD34_MOUSE | Q64314 | -2.25 | -4.86 | 2.61 | 0.023998 | No |
| MDHM_MOUSE | P08249 | 6.35 | 4.94 | 1.41 | 0.024177 | Yes |
| NDUV2_MOUSE | Q9D6J6 | 0.30 | -3.28 | 3.58 | 0.024182 | Yes |
| NIPS2_MOUSE | O55126 | 3.06 | 2.31 | 0.76 | 0.024568 | Yes |
| ATP5L_MOUSE | Q9CPQ8 | -3.45 | -5.46 | 2.01 | 0.025163 | Yes |
| 68MP_MOUSE | P56379 | -2.30 | -4.69 | 2.38 | 0.025386 | No |
| TM1L2_MOUSE | Q5SRX1 | -3.05 | -4.99 | 1.94 | 0.025570 | Yes |
| NDUBA_MOUSE | Q9DCS9 | 3.41 | 1.58 | 1.83 | 0.025575 | Yes |
| FLNA_MOUSE | Q8BTM8 | 0.59 | -2.46 | 3.05 | 0.025619 | Yes |
| CMC1_MOUSE | Q9CPZ8 | -2.06 | -4.30 | 2.24 | 0.025638 | Yes |
| RRBP1_MOUSE | Q99PL5 | 0.40 | -1.23 | 1.64 | 0.025895 | Yes |
| NDUBB_MOUSE | O09111 | 4.76 | 3.97 | 0.79 | 0.025942 | Yes |
| FUMH_MOUSE | P97807 | 2.20 | -1.83 | 4.03 | 0.026204 | Yes |
| RS12_MOUSE | P63323 | -3.73 | -5.66 | 1.92 | 0.026555 | Yes |
| AT1A2_MOUSE | Q6PIE5 | -1.14 | -3.84 | 2.70 | 0.027171 | Yes |
| GRHPR_MOUSE | Q91Z53 | -1.70 | -5.18 | 3.48 | 0.027511 | Yes |
| KNG1_MOUSE | O08677 | -0.48 | -2.63 | 2.15 | 0.027694 | No |
| RS21_MOUSE | Q9CQR2 | -1.40 | -3.81 | 2.41 | 0.027855 | Yes |
| FUBP2_MOUSE | Q3U0V1 | -4.23 | -5.47 | 1.24 | 0.028221 | Yes |
| CCHL_MOUSE | P53702 | -2.51 | -4.57 | 2.06 | 0.028633 | Yes |
| 1433Z_MOUSE | P63101 | 2.32 | 0.77 | 1.55 | 0.028791 | Yes |
| RL3_MOUSE | P27659 | -3.96 | -4.98 | 1.02 | 0.029487 | Yes |
| PGK1_MOUSE | P09411 | 2.58 | 1.57 | 1.01 | 0.029537 | Yes |
| SPRE_MOUSE | Q64105 | -0.87 | -3.11 | 2.23 | 0.029617 | Yes |
| ANT3_MOUSE | P32261 | -1.28 | -4.71 | 3.43 | 0.029790 | No |
| VINC_MOUSE | Q64727 | 0.20 | -2.73 | 2.92 | 0.030642 | Yes |
| UBA1_MOUSE | Q02053 | -2.45 | -4.19 | 1.74 | 0.030766 | Yes |
| FLNC_MOUSE | Q8VHX6 | 0.27 | -1.89 | 2.17 | 0.030938 | Yes |
| COX2_MOUSE | P00405 | 2.78 | 0.35 | 2.42 | 0.031094 | Yes |
| XIRP1_MOUSE | O70373 | -1.42 | -3.99 | 2.56 | 0.031134 | Yes |
| QCR9_MOUSE | Q8R1I1 | -1.42 | -3.94 | 2.52 | 0.031609 | Yes |
| THTM_MOUSE | Q99J99 | -2.95 | -4.64 | 1.69 | 0.031754 | Yes |
| DC1I2_MOUSE | O88487 | -1.29 | -3.84 | 2.55 | 0.031798 | Yes |
| SGCD_MOUSE | P82347 | -3.01 | -4.94 | 1.94 | 0.031901 | No |
| SUCB2_MOUSE | Q9Z2I8 | -1.46 | -3.81 | 2.35 | 0.032209 | Yes |
| DHB8_MOUSE | P50171 | -1.75 | -4.62 | 2.87 | 0.032228 | Yes |
| COX5B_MOUSE | P19536 | 6.79 | 5.43 | 1.36 | 0.032573 | Yes, |
| APOE_MOUSE | P08226 | -2.28 | -4.84 | 2.56 | 0.032778 | Yes |
| ANF_MOUSE | P05125 | 0.28 | -3.72 | 3.99 | 0.032950 | No |
| PIMT_MOUSE | P23506 | -0.21 | -2.07 | 1.86 | 0.033017 | Yes |
| ATPK_MOUSE | P56135 | 4.31 | 3.40 | 0.90 | 0.033236 | Yes |
| BASI_MOUSE | P18572 | -0.22 | -3.17 | 2.95 | 0.033832 | Yes |
| PSA1_MOUSE | Q9R1P4 | -1.02 | -4.63 | 3.61 | 0.033888 | Yes |
| OPA1_MOUSE | P58281 | -1.20 | -2.85 | 1.66 | 0.034390 | Yes |
| ANXA5_MOUSE | P48036 | -1.06 | -3.54 | 2.48 | 0.034499 | Yes |
| PABP1_MOUSE | P29341 | -3.38 | -5.09 | 1.71 | 0.035076 | Yes |
| LMNA_MOUSE | P48678 | -3.02 | -5.31 | 2.29 | 0.035599 | Yes |
| ALBU_MOUSE | P07724 | 5.20 | 2.89 | 2.31 | 0.035612 | Yes |
| ACADS_MOUSE | Q07417 | -0.62 | -3.51 | 2.89 | 0.036098 | Yes |
| MIC19_MOUSE | Q9CRB9 | 3.07 | 1.19 | 1.87 | 0.036290 | Yes |
| HXK1_MOUSE | P17710 | -0.82 | -2.40 | 1.59 | 0.036329 | Yes |
| RTN4_MOUSE | Q99P72 | -1.91 | -4.36 | 2.45 | 0.036333 | Yes |
| PGM1_MOUSE | Q9D0F9 | -3.00 | -4.67 | 1.67 | 0.036359 | Yes |
| USO1_MOUSE | Q9Z1Z0 | -2.46 | -4.48 | 2.03 | 0.036459 | Yes |
| ODP2_MOUSE | Q8BMF4 | 4.55 | 3.21 | 1.33 | 0.036617 | Yes |
| UBQL2_MOUSE | Q9QZM0 | -3.51 | -5.74 | 2.23 | 0.036720 | Yes |
| GLYG_MOUSE | Q9R062 | -0.55 | -3.66 | 3.11 | 0.037008 | Yes |
| MYH9_MOUSE | Q8VDD5 | -2.63 | -5.01 | 2.39 | 0.037288 | Yes |
| RS2_MOUSE | P25444 | -2.09 | -4.28 | 2.19 | 0.037503 | Yes |
| TELO2_MOUSE | Q9DC40 | 4.76 | 3.30 | 1.46 | 0.037611 | Yes |
| DESM_MOUSE | P31001 | -2.08 | -4.44 | 2.36 | 0.038075 | Yes |
| RS15_MOUSE | P62843 | -1.58 | -2.86 | 1.29 | 0.038250 | Yes |
| TAU_MOUSE | P10637 | -0.50 | -2.98 | 2.48 | 0.038330 | Yes |
| ANXA2_MOUSE | P07356 | -2.79 | -4.69 | 1.90 | 0.038343 | Yes |
| ACTN4_MOUSE | P57780 | -3.97 | -4.95 | 0.99 | 0.038390 | Yes |
| LAMB2_MOUSE | Q61292 | -4.08 | -5.57 | 1.49 | 0.038510 | Yes |
| B4GT1_MOUSE | P15535 | 2.26 | -0.59 | 2.85 | 0.039436 | Yes |
| HS90B_MOUSE | P11499 | 1.88 | -0.09 | 1.98 | 0.039681 | Yes |
| ATPB_MOUSE | P56480 | 8.22 | 7.26 | 0.96 | 0.039841 | Yes |
| PZP_MOUSE | Q61838 | -0.39 | -3.36 | 2.97 | 0.040026 | Yes |
| DBLOH_MOUSE | Q9JIQ3 | -3.15 | -4.80 | 1.64 | 0.040159 | Yes |
| NDUB8_MOUSE | Q9D6J5 | 4.26 | 3.42 | 0.85 | 0.040165 | Yes |
| HINT1_MOUSE | P70349 | 2.91 | 1.38 | 1.53 | 0.040639 | Yes |
| LETM1_MOUSE | Q9Z2I0 | -0.10 | -2.19 | 2.10 | 0.041120 | Yes |
| RS3_MOUSE | P62908 | 1.15 | 0.24 | 0.91 | 0.041365 | Yes |
| CLH1_MOUSE | Q68FD5 | -3.21 | -5.10 | 1.90 | 0.041409 | Yes |
| COQ9_MOUSE | Q8K1Z0 | 1.21 | -2.53 | 3.74 | 0.041481 | Yes |
| FRIL1_MOUSE | P29391 | -0.30 | -3.05 | 2.75 | 0.041916 | No |
| KINH_MOUSE | Q61768 | -0.73 | -3.14 | 2.41 | 0.042110 | Yes |
| ACADL_MOUSE | P51174 | 5.22 | 4.51 | 0.70 | 0.042221 | Yes |
| NDUS5_MOUSE | Q99LY9 | -0.44 | -3.90 | 3.46 | 0.042542 | Yes |
| DECR_MOUSE | Q9CQ62 | 1.59 | 0.10 | 1.48 | 0.043136 | No |
| GNAS2_MOUSE | P63094 | -3.37 | -4.96 | 1.59 | 0.043297 | Yes |
| UBE2N_MOUSE | P61089 | -2.22 | -4.83 | 2.60 | 0.043364 | Yes |
| AKAP1_MOUSE | O08715 | -2.66 | -4.39 | 1.72 | 0.043625 | Yes |
| MIC60_MOUSE | Q8CAQ8 | 2.39 | -0.90 | 3.29 | 0.044024 | Yes |
| DC1L1_MOUSE | Q8R1Q8 | -2.13 | -4.69 | 2.56 | 0.044594 | Yes |
| KAD2_MOUSE | Q9WTP6 | 1.87 | 0.18 | 1.69 | 0.044659 | Yes |
| PDIA1_MOUSE | P09103 | -1.87 | -4.26 | 2.39 | 0.044843 | Yes |
| AATC_MOUSE | P05201 | 2.02 | 0.01 | 2.01 | 0.045542 | Yes |
| H15_MOUSE | P43276 | 1.54 | -1.12 | 2.65 | 0.045582 | Yes |
| PICAL_MOUSE | Q7M6Y3 | -1.56 | -4.25 | 2.69 | 0.046503 | Yes |
| MIC26_MOUSE | Q9DCZ4 | -0.36 | -3.38 | 3.02 | 0.046638 | No |
| ANXA1_MOUSE | P10107 | -1.96 | -4.04 | 2.09 | 0.046907 | Yes |
| PCBP1_MOUSE | P60335 | -3.66 | -4.76 | 1.10 | 0.047293 | Yes |
| RL4_MOUSE | Q9D8E6 | -1.58 | -4.48 | 2.90 | 0.047507 | Yes |
| DNJA3_MOUSE | Q99M87 | -3.99 | -5.08 | 1.09 | 0.048128 | Yes |
| EF1G_MOUSE | Q9D8N0 | -1.64 | -4.06 | 2.42 | 0.049194 | Yes |
| ATP5I_MOUSE | Q06185 | 3.72 | 1.61 | 2.12 | 0.049277 | Yes |
| AUHM_MOUSE | Q9JLZ3 | -1.52 | -4.61 | 3.09 | 0.049292 | No |
| EIF3A_MOUSE | P23116 | -0.63 | -3.50 | 2.87 | 0.049343 | Yes |
| LAP2B_MOUSE | Q61029 | -2.37 | -3.84 | 1.48 | 0.049879 | Yes |
| AATM_MOUSE | P05202 | 4.89 | 3.76 | 1.14 | 0.050668 | Yes |
| NDKB_MOUSE | Q01768 | 1.48 | -0.43 | 1.91 | 0.051038 | Yes |
| VDAC2_MOUSE | Q60930 | -0.49 | -3.23 | 2.73 | 0.052088 | Yes |
| ATPD_MOUSE | Q9D3D9 | 3.71 | 2.57 | 1.14 | 0.052094 | Yes |
| ROA1_MOUSE | P49312 | -0.61 | -3.37 | 2.76 | 0.052387 | Yes |
| CX6B1_MOUSE | P56391 | 1.02 | 0.41 | 0.61 | 0.053323 | Yes |
| HSPB8_MOUSE | Q9JK92 | 0.13 | -2.42 | 2.55 | 0.053350 | Yes |
| MYPN_MOUSE | Q5DTJ9 | -2.26 | -4.34 | 2.08 | 0.054263 | Yes |
| KTN1_MOUSE | Q61595 | -1.97 | -4.31 | 2.34 | 0.054603 | Yes |
| CATA_MOUSE | P24270 | -0.77 | -2.73 | 1.96 | 0.054893 | Yes |
| AAK1_MOUSE | Q3UHJ0 | -3.48 | -5.69 | 2.21 | 0.054957 | Yes |
| TXLNB_MOUSE | Q8VBT1 | -0.75 | -3.27 | 2.52 | 0.055160 | No |
| STML2_MOUSE | Q99JB2 | -1.47 | -3.21 | 1.74 | 0.055298 | Yes |
| H13_MOUSE | P43277 | 2.67 | 0.94 | 1.73 | 0.055503 | Yes |
| RL10A_MOUSE | P53026 | -1.71 | -4.10 | 2.39 | 0.055523 | Yes |
| SLMAP_MOUSE | Q3URD3 | 0.50 | -3.62 | 4.12 | 0.055568 | Yes |
| THIO_MOUSE | P10639 | -0.28 | -2.65 | 2.37 | 0.055729 | Yes |
| MARE2_MOUSE | Q8R001 | -3.79 | -5.25 | 1.46 | 0.055963 | Yes |
| EF1A1_MOUSE | P10126 | -0.05 | -2.09 | 2.04 | 0.056257 | Yes |
| TAGL2_MOUSE | Q9WVA4 | 2.27 | 0.54 | 1.73 | 0.056305 | Yes |
| NDUA7_MOUSE | Q9Z1P6 | 5.51 | 4.67 | 0.84 | 0.057808 | Yes |
| PFKAL_MOUSE | P12382 | -3.11 | -5.03 | 1.91 | 0.058700 | Yes |
| ECHM_MOUSE | Q8BH95 | 1.58 | -0.10 | 1.68 | 0.058825 | Yes |
| FXR1_MOUSE | Q61584 | -2.46 | -4.76 | 2.30 | 0.058971 | Yes |
| MSRB2_MOUSE | Q78J03 | -1.33 | -3.72 | 2.39 | 0.059338 | No |
| HBA_MOUSE | P01942 | 8.42 | 7.55 | 0.88 | 0.059473 | Yes |
| RPN1_MOUSE | Q91YQ5 | -3.14 | -5.07 | 1.93 | 0.059498 | Yes |
| HIBCH_MOUSE | Q8QZS1 | -0.91 | -3.51 | 2.60 | 0.059755 | Yes |
| NNRE_MOUSE | Q8K4Z3 | -2.31 | -3.89 | 1.58 | 0.059891 | Yes |
| HSDL2_MOUSE | Q2TPA8 | 2.79 | 1.73 | 1.06 | 0.059988 | Yes |
| NDUAA_MOUSE | Q99LC3 | 1.82 | -0.46 | 2.29 | 0.060343 | Yes |
| EHD4_MOUSE | Q9EQP2 | 0.06 | -1.94 | 2.00 | 0.060893 | Yes |
| RL28_MOUSE | P41105 | -1.89 | -3.24 | 1.35 | 0.061838 | Yes |
| AL4A1_MOUSE | Q8CHT0 | 0.81 | -1.85 | 2.66 | 0.061889 | Yes |
| LPP_MOUSE | Q8BFW7 | -0.98 | -3.91 | 2.92 | 0.062086 | Yes |
| CSRP3_MOUSE | P50462 | 5.32 | 4.28 | 1.04 | 0.062164 | Yes |
| RL6_MOUSE | P47911 | 1.30 | -0.63 | 1.94 | 0.062421 | Yes |
| MYPT1_MOUSE | Q9DBR7 | -0.94 | -2.88 | 1.93 | 0.062972 | Yes |
| MYG_MOUSE | P04247 | 7.93 | 6.96 | 0.97 | 0.063249 | Yes |
| KCRM_MOUSE | P07310 | 4.52 | 3.36 | 1.16 | 0.063581 | Yes |
| 1433B_MOUSE | Q9CQV8 | 0.23 | -2.67 | 2.90 | 0.064000 | Yes |
| NRAP_MOUSE | Q80XB4 | -3.56 | -5.11 | 1.55 | 0.064272 | Yes |
| SRBS2_MOUSE | Q3UTJ2 | 1.51 | -0.72 | 2.23 | 0.065105 | Yes |
| ACTG_MOUSE | P63260 | -1.74 | -3.57 | 1.83 | 0.065782 | Yes |
| ZYX_MOUSE | Q62523 | 1.13 | -0.52 | 1.65 | 0.066190 | Yes |
| NDUB4_MOUSE | Q9CQC7 | 5.23 | 4.01 | 1.23 | 0.067077 | Yes |
| USMG5_MOUSE | Q78IK2 | -1.42 | -3.70 | 2.29 | 0.067196 | No |
| RS19_MOUSE | Q9CZX8 | -1.33 | -3.87 | 2.54 | 0.067674 | Yes |
| NMT1_MOUSE | O70310 | -2.52 | -4.24 | 1.72 | 0.068662 | Yes |
| ACOX1_MOUSE | Q9R0H0 | -1.95 | -4.06 | 2.11 | 0.068673 | Yes |
| NDUB5_MOUSE | Q9CQH3 | 3.84 | 2.97 | 0.87 | 0.068959 | Yes |
| DDX5_MOUSE | Q61656 | -3.98 | -4.99 | 1.01 | 0.068975 | Yes |
| MYL3_MOUSE | P09542 | 3.37 | 2.65 | 0.72 | 0.069375 | Yes |
| LNEBL_MOUSE | Q9DC07 | -1.64 | -3.70 | 2.06 | 0.069551 | Yes |
| NLTP_MOUSE | P32020 | -2.20 | -3.71 | 1.51 | 0.070081 | No |
| OCAD1_MOUSE | Q9CRD0 | -0.13 | -2.77 | 2.64 | 0.071026 | Yes |
| RL14_MOUSE | Q9CR57 | -0.61 | -3.06 | 2.45 | 0.071290 | Yes |
| RS9_MOUSE | Q6ZWN5 | -0.40 | -1.81 | 1.41 | 0.071305 | Yes |
| PDLI5_MOUSE | Q8CI51 | 2.57 | 0.57 | 2.00 | 0.071430 | Yes |
| IASPP_MOUSE | Q5I1X5 | -3.40 | -5.16 | 1.76 | 0.072131 | Yes |
| RS13_MOUSE | P62301 | -2.31 | -4.56 | 2.25 | 0.073124 | Yes |
| RS10_MOUSE | P63325 | -0.77 | -3.38 | 2.61 | 0.073179 | Yes |
| PPIA_MOUSE | P17742 | 2.44 | 0.18 | 2.26 | 0.073541 | Yes |
| CAV3_MOUSE | P51637 | -0.20 | -2.37 | 2.17 | 0.073576 | No |
| COXM1_MOUSE | Q9CPZ8 | -2.21 | -3.72 | 1.51 | 0.073866 | No |
| SRCA_MOUSE | Q7TQ48 | 5.52 | 4.48 | 1.03 | 0.074476 | No |
| GLNA_MOUSE | P15105 | -3.36 | -5.32 | 1.96 | 0.074920 | Yes |
| PERM1_MOUSE | Q149B8 | -2.06 | -3.87 | 1.82 | 0.075219 | No |
| SIR5_MOUSE | Q8K2C6 | -0.80 | -2.98 | 2.19 | 0.075628 | No |
| QCR1_MOUSE | Q9CZ13 | 2.81 | 1.13 | 1.68 | 0.075733 | Yes |
| IF4G2_MOUSE | Q62448 | -3.33 | -5.00 | 1.67 | 0.075851 | Yes |
| NDUS3_MOUSE | Q9DCT2 | 2.85 | 1.51 | 1.33 | 0.075963 | Yes |
| FBX40_MOUSE | P62932 | -3.82 | -4.90 | 1.09 | 0.076436 | No |
| NDUA5_MOUSE | Q9CPP6 | -1.79 | -3.39 | 1.60 | 0.076503 | No |
| ANXA4_MOUSE | P97429 | -2.41 | -3.96 | 1.55 | 0.077730 | Yes |
| VDAC1_MOUSE | Q60932 | 3.59 | 2.91 | 0.68 | 0.078143 | Yes |
| RL13_MOUSE | P47963 | 0.36 | -2.32 | 2.68 | 0.078493 | Yes |
| NDUB1_MOUSE | P0DN34 | 3.25 | 2.41 | 0.84 | 0.079787 | No |
| MYPT2_MOUSE | Q8BG95 | -1.22 | -3.48 | 2.26 | 0.081661 | Yes |
| UBAP2_MOUSE | Q91VX2 | 0.46 | -1.07 | 1.53 | 0.082033 | Yes |
| SRBS1_MOUSE | Q62417 | -2.22 | -3.99 | 1.77 | 0.082048 | Yes |
| AT1B1_MOUSE | P14094 | -1.50 | -4.14 | 2.64 | 0.082313 | Yes |
| FHOD3_MOUSE | Q76LL6 | -3.57 | -4.66 | 1.09 | 0.082323 | Yes |
| AT2A2_MOUSE | O55143 | 4.61 | 3.84 | 0.77 | 0.082640 | Yes |
| CLPP_MOUSE | O88696 | -2.80 | -4.70 | 1.90 | 0.084550 | No |
| NAR3_MOUSE | Q8R2G4 | -2.82 | -4.87 | 2.05 | 0.084711 | No |
| H2B1F_MOUSE | P10853 | 1.35 | 0.27 | 1.08 | 0.084902 | Yes |
| CASQ2_MOUSE | O09161 | 2.21 | 1.36 | 0.85 | 0.085586 | No |
| MCEE_MOUSE | Q9D1I5 | -2.09 | -2.95 | 0.86 | 0.085720 | No |
| BOLA3_MOUSE | Q8CEI1 | -3.58 | -5.16 | 1.58 | 0.086102 | No |
| NPM_MOUSE | Q61937 | -1.60 | -3.36 | 1.76 | 0.086463 | Yes |
| PRS30_MOUSE | Q9QYZ9 | 2.57 | 3.23 | -0.66 | 0.086482 | No |
| SCOT1_MOUSE | Q9D0K2 | -0.38 | -2.77 | 2.40 | 0.086633 | Yes |
| ECI1_MOUSE | P42125 | 4.06 | 1.89 | 2.18 | 0.086770 | Yes |
| ETFB_MOUSE | Q9DCW4 | 4.66 | 3.47 | 1.19 | 0.087522 | Yes |
| CX7A1_MOUSE | P56392 | 2.38 | 0.77 | 1.61 | 0.088773 | No |
| GSTM2_MOUSE | P15626 | -1.67 | -4.01 | 2.34 | 0.089227 | Yes |
| CACP_MOUSE | P47934 | -1.47 | -3.16 | 1.69 | 0.089757 | Yes |
| H11_MOUSE | P43275 | 0.86 | -0.18 | 1.04 | 0.089867 | Yes |
| TNNT2_MOUSE | P50752 | -0.13 | -3.09 | 2.96 | 0.089870 | Yes |
| IMB1_MOUSE | P70168 | -5.58 | -4.43 | -1.15 | 0.089918 | Yes |
| MCCB_MOUSE | Q3ULD5 | -2.70 | -4.19 | 1.50 | 0.090842 | Yes |
| AT5F1_MOUSE | Q9CQQ7 | 3.72 | 2.71 | 1.01 | 0.091351 | Yes |
| LIMC1_MOUSE | Q3UH68 | -2.03 | -3.29 | 1.26 | 0.092384 | Yes |
| CATB_MOUSE | P10605 | -2.77 | -4.20 | 1.43 | 0.093026 | Yes |
| RS25_MOUSE | P62852 | 0.72 | 0.14 | 0.58 | 0.093721 | Yes |
| HBB1_MOUSE | P02088 | 5.70 | 4.53 | 1.17 | 0.094010 | No |
| FABPH_MOUSE | P11404 | 5.35 | 4.44 | 0.91 | 0.094507 | No |
| WNK1_MOUSE | P83741 | -1.88 | -3.71 | 1.83 | 0.094659 | Yes |
| CLUS_MOUSE | Q06890 | -0.67 | -3.34 | 2.67 | 0.094855 | Yes |
| CAPZB_MOUSE | P47757 | -3.29 | -4.85 | 1.56 | 0.095274 | Yes |
| RL19_MOUSE | P84099 | -0.32 | -2.59 | 2.27 | 0.095768 | Yes |
| ROA2_MOUSE | O88569 | 2.48 | 1.10 | 1.38 | 0.095787 | Yes |
| EFTU_MOUSE | Q8BFR5 | 1.56 | -0.11 | 1.66 | 0.095822 | Yes |
| G3P_MOUSE | P16858 | 6.96 | 6.38 | 0.57 | 0.096797 | Yes |
| COQ7_MOUSE | P97478 | -4.99 | -6.00 | 1.01 | 0.096846 | No |
| NDUB9_MOUSE | Q9CQJ8 | 2.19 | 1.26 | 0.93 | 0.098562 | Yes |
| MDHC_MOUSE | P14152 | 4.06 | 3.21 | 0.85 | 0.099245 | Yes |
| ATPO_MOUSE | Q9DB20 | 4.61 | 3.46 | 1.15 | 0.100121 | Yes |
| ACS2L_MOUSE | Q99NB1 | -3.37 | -4.83 | 1.46 | 0.100201 | No |
| CLCA_MOUSE | O08585 | -4.24 | -5.77 | 1.53 | 0.100266 | Yes |
| SPG20_MOUSE | Q8R1X6 | -3.00 | -2.11 | -0.89 | 0.100970 | No |
| SRSF6_MOUSE | Q3TWW8 | -3.64 | -4.74 | 1.10 | 0.101792 | Yes |
| PRDX3_MOUSE | P20108 | 1.01 | -0.32 | 1.33 | 0.102255 | Yes |
| NDUA1_MOUSE | O35683 | 0.75 | -0.76 | 1.51 | 0.102945 | Yes |
| HCFC1_MOUSE | Q61191 | -2.03 | -3.57 | 1.54 | 0.103721 | Yes |
| NDRG2_MOUSE | Q9QYG0 | 2.75 | 1.12 | 1.63 | 0.104535 | No |
| NDUA4_MOUSE | Q62425 | 5.38 | 4.78 | 0.60 | 0.104793 | Yes |
| PACN2_MOUSE | Q9WVE8 | -1.33 | -2.40 | 1.07 | 0.104822 | Yes |
| SUCB1_MOUSE | Q9Z2I9 | 1.69 | -0.68 | 2.37 | 0.104981 | Yes |
| HMGCL_MOUSE | P38060 | -1.44 | -3.55 | 2.11 | 0.105505 | Yes |
| TPIS_MOUSE | P17751 | 2.42 | 1.01 | 1.41 | 0.106343 | Yes |
| NDUB2_MOUSE | Q9CPU2 | 1.51 | -0.14 | 1.65 | 0.107705 | No |
| HSP7C_MOUSE | P63017 | 2.90 | 1.95 | 0.94 | 0.107754 | Yes |
| ODO2_MOUSE | Q9D2G2 | 2.45 | 1.34 | 1.12 | 0.107874 | Yes |
| NQO1_MOUSE | Q64669 | -1.33 | -3.25 | 1.92 | 0.108134 | Yes |
| RCN3_MOUSE | Q8BH97 | -4.43 | -5.89 | 1.46 | 0.108284 | Yes |
| ATP5H_MOUSE | Q9DCX2 | 4.74 | 3.58 | 1.15 | 0.108449 | Yes |
| SPEG_MOUSE | Q62407 | -0.24 | -2.13 | 1.89 | 0.108457 | Yes |
| ACO13_MOUSE | Q9CQR4 | 0.38 | -0.48 | 0.86 | 0.108517 | No |
| FKB1A_MOUSE | P26883 | 1.73 | 1.05 | 0.69 | 0.108552 | Yes |
| CALR_MOUSE | P14211 | -0.40 | -0.70 | 0.31 | 0.108881 | Yes |
| CX7A2_MOUSE | P48771 | -2.80 | -4.26 | 1.45 | 0.109489 | Yes |
| ODPX_MOUSE | Q8BKZ9 | 0.14 | -2.40 | 2.55 | 0.109753 | No |
| RS11_MOUSE | P62281 | -0.18 | -0.63 | 0.45 | 0.110125 | Yes |
| TOM22_MOUSE | Q9CPQ3 | -2.49 | -3.49 | 1.00 | 0.110162 | No |
| SSDH_MOUSE | Q8BWF0 | -2.75 | -3.77 | 1.02 | 0.110313 | Yes |
| HNRPM_MOUSE | Q9D0E1 | -0.90 | -2.81 | 1.90 | 0.110727 | Yes |
| MCCA_MOUSE | Q99MR8 | -2.92 | -4.39 | 1.47 | 0.111636 | Yes |
| TERA_MOUSE | Q01853 | 1.87 | 0.33 | 1.54 | 0.112541 | Yes |
| IGH1M_MOUSE | P01869 | 7.92 | 6.56 | 1.35 | 0.112744 | No |
| IGHG1_MOUSE | P01868 | 7.92 | 6.56 | 1.35 | 0.112744 | Yes |
| CISD1_MOUSE | Q91WS0 | -1.15 | -3.29 | 2.14 | 0.112795 | Yes |
| THIM_MOUSE | Q8BWT1 | 5.63 | 4.77 | 0.86 | 0.112895 | Yes |
| MA7D1_MOUSE | A2AJI0 | -2.00 | -3.95 | 1.95 | 0.113016 | Yes |
| NDUB7_MOUSE | Q9CR61 | 4.71 | 5.07 | -0.36 | 0.113061 | Yes |
| SDHF1_MOUSE | Q3U276 | -4.12 | -5.27 | 1.14 | 0.113653 | No |
| PDIA3_MOUSE | P27773 | 0.13 | -2.34 | 2.47 | 0.114480 | Yes |
| EEA1_MOUSE | Q8BL66 | -4.23 | -5.07 | 0.84 | 0.114612 | Yes |
| ODPA_MOUSE | P35486 | 4.67 | 2.92 | 1.75 | 0.114660 | Yes |
| QCR8_MOUSE | Q9CQ69 | 4.27 | 3.32 | 0.95 | 0.114824 | Yes |
| TPM1_MOUSE | P58771 | 1.64 | -0.67 | 2.31 | 0.116087 | Yes |
| ATP5E_MOUSE | P56382 | -0.68 | -2.73 | 2.05 | 0.117602 | Yes |
| KAD1_MOUSE | Q9R0Y5 | 2.65 | 1.37 | 1.29 | 0.117912 | Yes |
| PPR3A_MOUSE | Q99MR9 | -2.37 | -4.12 | 1.75 | 0.118316 | No |
| SH3L3_MOUSE | Q91VW3 | -3.12 | -4.23 | 1.11 | 0.118344 | Yes |
| PALLD_MOUSE | Q9ET54 | -3.39 | -4.36 | 0.97 | 0.118398 | Yes |
| RL27_MOUSE | P61358 | -3.73 | -4.59 | 0.86 | 0.119565 | Yes |
| MIF_MOUSE | P34884 | 1.61 | 2.08 | -0.47 | 0.120191 | Yes |
| SPA3K_MOUSE | P07759 | -0.20 | -2.06 | 1.85 | 0.121284 | No |
| HMGB1_MOUSE | P63158 | -0.97 | -2.99 | 2.02 | 0.122233 | Yes |
| PHB2_MOUSE | O35129 | 0.15 | -1.79 | 1.94 | 0.123071 | Yes |
| SDHA_MOUSE | Q8K2B3 | 3.32 | 2.51 | 0.80 | 0.125092 | Yes |
| LDHB_MOUSE | P16125 | 2.63 | 1.23 | 1.41 | 0.125935 | Yes |
| CO1A1_MOUSE | P11087 | -2.13 | -3.80 | 1.68 | 0.125963 | Yes |
| HSP74_MOUSE | Q61316 | -1.13 | -2.27 | 1.14 | 0.126310 | Yes |
| KPYM_MOUSE | P52480 | 1.87 | 0.68 | 1.18 | 0.126464 | Yes |
| C560_MOUSE | Q9CZB0 | -1.52 | -3.45 | 1.92 | 0.126899 | No |
| SYEP_MOUSE | Q8CGC7 | -3.28 | -4.42 | 1.14 | 0.126994 | Yes |
| ETFA_MOUSE | Q99LC5 | 4.10 | 3.10 | 1.00 | 0.127513 | Yes |
| CAND2_MOUSE | Q6ZQ73 | -4.06 | -5.11 | 1.05 | 0.127811 | Yes |
| FETUA_MOUSE | P29699 | 1.23 | 0.00 | 1.23 | 0.128128 | Yes |
| HIG1A_MOUSE | Q9JLR9 | 1.51 | 2.32 | -0.81 | 0.128224 | Yes |
| QCR2_MOUSE | Q9DB77 | 4.68 | 4.01 | 0.67 | 0.128425 | Yes |
| 1433G_MOUSE | P61982 | 3.04 | 2.39 | 0.65 | 0.128439 | Yes |
| PTRF_MOUSE | O54724 | 3.73 | 2.87 | 0.86 | 0.128585 | No |
| CFAB_MOUSE | P04186 | -3.86 | -4.77 | 0.92 | 0.130011 | Yes |
| MIC13_MOUSE | Q8R404 | 0.89 | -0.18 | 1.06 | 0.130402 | Yes |
| MURC_MOUSE | A2AMM0 | -1.81 | -3.73 | 1.92 | 0.131273 | No |
| RS27A_MOUSE | P62983 | -0.43 | -2.78 | 2.35 | 0.132216 | Yes |
| PHB_MOUSE | Q3TV00 | 1.51 | 0.02 | 1.49 | 0.133318 | Yes |
| KCRS_MOUSE | Q6P8J7 | 5.00 | 3.83 | 1.17 | 0.134291 | Yes |
| LDHA_MOUSE | P06151 | 0.34 | -0.89 | 1.23 | 0.134900 | Yes |
| ODBA_MOUSE | P50136 | -0.84 | -1.71 | 0.87 | 0.134997 | Yes |
| HUWE1_MOUSE | Q7TMY8 | -2.90 | -3.56 | 0.66 | 0.135505 | Yes |
| EF1B_MOUSE | O70251 | -3.05 | -4.53 | 1.48 | 0.135552 | Yes |
| ACON_MOUSE | Q99KI0 | 6.22 | 5.59 | 0.62 | 0.135720 | Yes |
| MYPC3_MOUSE | O70468 | -0.37 | -1.92 | 1.55 | 0.135741 | Yes |
| CYB5B_MOUSE | Q9CQX2 | -2.03 | -3.79 | 1.76 | 0.137294 | Yes |
| CLYBL_MOUSE | Q8R4N0 | -2.45 | -4.03 | 1.58 | 0.138374 | No |
| KCRB_MOUSE | Q04447 | -2.39 | -3.89 | 1.51 | 0.138712 | Yes |
| NDUB6_MOUSE | Q3UIU2 | 3.46 | 2.83 | 0.63 | 0.140369 | Yes |
| RS3A_MOUSE | P97351 | 2.12 | 1.37 | 0.76 | 0.140583 | Yes |
| TLN1_MOUSE | P26039 | -2.54 | -4.22 | 1.68 | 0.142605 | Yes |
| MTCH1_MOUSE | Q791T5 | 0.02 | 0.39 | -0.37 | 0.143087 | No |
| CH60_MOUSE | P63038 | 4.93 | 4.12 | 0.81 | 0.144430 | Yes |
| ENPL_MOUSE | P08113 | 1.20 | 0.10 | 1.10 | 0.147576 | Yes |
| NDUAD_MOUSE | Q9ERS2 | 1.89 | -0.07 | 1.96 | 0.148120 | Yes |
| TCPD_MOUSE | P80315 | -0.74 | -2.56 | 1.82 | 0.148279 | Yes |
| MOT1_MOUSE | P53986 | 2.40 | 2.81 | -0.41 | 0.148616 | Yes |
| SDPR_MOUSE | Q63918 | 1.96 | 1.32 | 0.63 | 0.150942 | No |
| ANXA6_MOUSE | P14824 | -1.62 | -3.27 | 1.64 | 0.152317 | Yes |
| IDHP_MOUSE | P54071 | 3.18 | 1.92 | 1.26 | 0.152396 | Yes |
| MIC10_MOUSE | Q7TNS2 | -0.09 | -0.64 | 0.55 | 0.153432 | No |
| RL18_MOUSE | P35980 | -1.29 | -3.13 | 1.84 | 0.153585 | Yes |
| MAP4_MOUSE | P27546 | 4.15 | 3.70 | 0.45 | 0.153850 | Yes |
| EIF3H_MOUSE | Q91WK2 | -4.35 | -4.94 | 0.59 | 0.154905 | Yes |
| MMSA_MOUSE | Q9EQ20 | -0.25 | -2.07 | 1.82 | 0.155143 | Yes |
| PLIN5_MOUSE | Q8BVZ1 | -2.80 | -4.12 | 1.32 | 0.156206 | Yes |
| H14_MOUSE | P43274 | 4.61 | 3.96 | 0.65 | 0.156977 | Yes |
| RT28_MOUSE | Q9CY16 | -1.33 | -2.43 | 1.10 | 0.158355 | No |
| HNRPK_MOUSE | P61979 | 0.07 | -1.72 | 1.79 | 0.159195 | Yes |
| ALPK3_MOUSE | Q924C5 | -4.64 | -5.30 | 0.65 | 0.160346 | No |
| RL31_MOUSE | P62900 | 0.11 | -1.87 | 1.98 | 0.160729 | Yes |
| HSPB6_MOUSE | Q5EBG6 | 2.36 | 1.98 | 0.38 | 0.160787 | Yes |
| IDH3A_MOUSE | Q9D6R2 | 2.49 | 0.77 | 1.72 | 0.161473 | Yes |
| PYGM_MOUSE | Q9WUB3 | 3.22 | 2.59 | 0.63 | 0.163404 | Yes |
| PSMD2_MOUSE | Q8VDM4 | -3.70 | -4.74 | 1.04 | 0.164131 | Yes |
| SUCA_MOUSE | Q9WUM5 | 0.70 | -0.30 | 1.00 | 0.164304 | Yes |
| CAD13_MOUSE | Q9WTR5 | 0.67 | -0.49 | 1.16 | 0.165126 | Yes |
| ACSL1_MOUSE | P41216 | -0.69 | -2.13 | 1.43 | 0.166885 | Yes |
| SYIM_MOUSE | Q8BIJ6 | -2.55 | -3.74 | 1.19 | 0.167239 | Yes |
| PGBM_MOUSE | Q05793 | -2.53 | -4.06 | 1.54 | 0.168296 | Yes |
| PRDX2_MOUSE | Q61171 | 3.04 | 2.79 | 0.25 | 0.168776 | Yes |
| RL8_MOUSE | P62918 | -1.22 | -2.77 | 1.55 | 0.168883 | Yes |
| TBB4B_MOUSE | P68372 | 0.46 | -0.54 | 1.00 | 0.169301 | Yes |
| TIDC1_MOUSE | Q8BUY5 | -2.45 | -3.86 | 1.41 | 0.169584 | No |
| UBP5_MOUSE | P56399 | -3.99 | -4.96 | 0.97 | 0.170090 | Yes |
| CY1_MOUSE | Q9D0M3 | 4.82 | 4.32 | 0.50 | 0.170877 | Yes |
| LPPRC_MOUSE | Q6PB66 | -1.24 | -2.63 | 1.39 | 0.171140 | Yes |
| CD36_MOUSE | Q08857 | -0.31 | -1.91 | 1.59 | 0.171147 | Yes |
| FIBA_MOUSE | E9PV24 | 3.72 | 4.11 | -0.40 | 0.172496 | No |
| RL24_MOUSE | Q8BP67 | -0.41 | -2.06 | 1.65 | 0.172672 | Yes |
| CH10_MOUSE | Q64433 | 1.83 | 0.87 | 0.96 | 0.174144 | Yes |
| COBL1_MOUSE | Q3UMF0 | -3.51 | -4.66 | 1.15 | 0.174178 | Yes |
| ENOB_MOUSE | P21550 | 2.50 | 1.54 | 0.96 | 0.175406 | Yes |
| UBXN1_MOUSE | Q922Y1 | -4.33 | -5.09 | 0.75 | 0.175994 | Yes |
| FIBB_MOUSE | Q8K0E8 | 0.08 | -0.75 | 0.83 | 0.176033 | Yes |
| DMD_MOUSE | P11531 | -1.69 | -3.40 | 1.71 | 0.176702 | Yes |
| MLRV_MOUSE | P51667 | -0.27 | -1.73 | 1.46 | 0.178081 | Yes |
| ADT2_MOUSE | P51881 | 0.05 | -0.92 | 0.97 | 0.180096 | Yes |
| GTR4_MOUSE | P14142 | -1.64 | -2.92 | 1.29 | 0.180821 | No |
| ODPB_MOUSE | Q9D051 | 3.29 | 2.24 | 1.05 | 0.182019 | Yes |
| GRP75_MOUSE | P38647 | 4.27 | 3.29 | 0.98 | 0.182634 | Yes |
| CYC_MOUSE | P62897 | 5.63 | 5.20 | 0.43 | 0.184877 | Yes |
| TALDO_MOUSE | Q93092 | -0.16 | 0.25 | -0.41 | 0.185173 | Yes |
| EMAL1_MOUSE | Q05BC3 | -3.73 | -4.99 | 1.26 | 0.185275 | Yes |
| TNNI3_MOUSE | P48787 | 5.04 | 5.43 | -0.39 | 0.186119 | Yes |
| EF2_MOUSE | P58252 | -0.51 | -1.90 | 1.39 | 0.186203 | Yes |
| NDUA6_MOUSE | Q9CQZ5 | 2.14 | 1.87 | 0.27 | 0.187011 | No |
| NDUS6_MOUSE | P52503 | 4.27 | 3.85 | 0.41 | 0.190152 | Yes |
| ATPA_MOUSE | Q03265 | 7.55 | 7.10 | 0.45 | 0.192527 | Yes |
| MPCP_MOUSE | Q8VEM8 | 2.50 | 1.82 | 0.68 | 0.193762 | Yes |
| SSBP_MOUSE | Q9CYR0 | -1.53 | -3.00 | 1.47 | 0.194409 | Yes |
| H32_MOUSE | P84228 | -2.94 | -4.79 | 1.85 | 0.195262 | Yes |
| SF01_MOUSE | Q64213 | -1.74 | -3.12 | 1.38 | 0.195496 | Yes |
| JPH2_MOUSE | Q9ET78 | 2.49 | 2.10 | 0.39 | 0.197839 | No |
| MPC1_MOUSE | P63030 | -0.83 | -2.31 | 1.48 | 0.199225 | No |
| ALDR_MOUSE | P45376 | 0.61 | -0.42 | 1.03 | 0.200342 | Yes |
| AT5G2_MOUSE | P56383 | 4.89 | 4.40 | 0.49 | 0.202094 | No |
| LA_MOUSE | P32067 | -4.48 | -3.88 | -0.61 | 0.203195 | No |
| UGPA_MOUSE | Q91ZJ5 | -0.74 | -1.99 | 1.25 | 0.204582 | Yes |
| EIF2A_MOUSE | Q8BJW6 | -2.47 | -3.37 | 0.90 | 0.204864 | Yes |
| HRG_MOUSE | Q9ESB3 | -1.64 | -2.77 | 1.12 | 0.206096 | No |
| TBA1B_MOUSE | P05213 | -0.92 | -2.40 | 1.48 | 0.206771 | Yes |
| CPT2_MOUSE | P52825 | -1.25 | -2.79 | 1.54 | 0.208917 | Yes |
| TBCA_MOUSE | P48428 | -1.68 | -2.96 | 1.27 | 0.209395 | Yes |
| AT1A1_MOUSE | Q8VDN2 | 2.60 | 1.99 | 0.61 | 0.210142 | Yes |
| ENOA_MOUSE | P17182 | -3.16 | -4.37 | 1.20 | 0.210981 | Yes |
| CAPG_MOUSE | P24452 | -3.82 | -4.47 | 0.66 | 0.211313 | No |
| ACTC_MOUSE | P68033 | 4.61 | 4.28 | 0.33 | 0.211535 | Yes |
| PLM_MOUSE | Q9Z239 | -0.77 | -2.38 | 1.61 | 0.212555 | No |
| PYGB_MOUSE | Q8CI94 | 1.95 | 1.32 | 0.63 | 0.213614 | Yes |
| ATPG_MOUSE | Q91VR2 | 3.66 | 2.81 | 0.85 | 0.213719 | Yes |
| NDUA9_MOUSE | Q9DC69 | 1.30 | 0.22 | 1.07 | 0.215251 | Yes |
| ACTH_MOUSE | P63268 | -2.50 | -1.48 | -1.02 | 0.215548 | Yes |
| K2C5_MOUSE | Q922U2 | -3.91 | -4.81 | 0.90 | 0.216672 | Yes |
| RS20_MOUSE | P60867 | -4.22 | -3.35 | -0.86 | 0.216896 | Yes |
| POPD1_MOUSE | Q9ES83 | -4.16 | -5.18 | 1.01 | 0.217173 | No |
| RL22_MOUSE | P67984 | -3.44 | -4.32 | 0.87 | 0.218092 | Yes |
| HNRPC_MOUSE | Q9Z204 | -2.69 | -3.52 | 0.83 | 0.218109 | Yes |
| ECH1_MOUSE | O35459 | 2.85 | 3.09 | -0.24 | 0.218574 | Yes |
| COA3_MOUSE | Q9D2R6 | -1.71 | -2.58 | 0.87 | 0.220471 | No |
| ATX2_MOUSE | O70305 | -2.28 | -3.65 | 1.38 | 0.220665 | Yes |
| SH3BG_MOUSE | Q9WUZ7 | 0.16 | -1.09 | 1.25 | 0.221368 | No |
| APOA1_MOUSE | Q00623 | -3.38 | -4.42 | 1.04 | 0.222321 | No |
| COR1B_MOUSE | Q9WUM3 | -3.25 | -4.24 | 0.99 | 0.223879 | Yes |
| TOM70_MOUSE | Q9CZW5 | -4.02 | -4.93 | 0.91 | 0.224060 | Yes |
| EI3JA_MOUSE | Q3UGC7 | -1.39 | -2.27 | 0.88 | 0.224483 | No |
| PPIF_MOUSE | Q99KR7 | 3.32 | 2.67 | 0.65 | 0.225859 | Yes |
| RL21_MOUSE | O09167 | -0.63 | -1.18 | 0.55 | 0.226564 | Yes |
| ETFD_MOUSE | Q921G7 | 5.58 | 5.95 | -0.37 | 0.227269 | Yes |
| RM24_MOUSE | Q9CQ06 | -3.75 | -4.49 | 0.74 | 0.227567 | Yes |
| NDUS4_MOUSE | Q9CXZ1 | 1.11 | -0.13 | 1.25 | 0.227890 | Yes |
| HINT2_MOUSE | Q9D0S9 | 2.28 | 1.83 | 0.45 | 0.228025 | Yes |
| LUM_MOUSE | P51885 | -4.09 | -4.90 | 0.81 | 0.228992 | Yes |
| M2OM_MOUSE | Q9CR62 | 2.84 | 2.48 | 0.36 | 0.230297 | Yes |
| GSTM1_MOUSE | P10649 | 1.11 | 0.60 | 0.51 | 0.230599 | Yes |
| MMAB_MOUSE | Q9D273 | -4.03 | -4.68 | 0.66 | 0.233210 | Yes |
| H2A1_MOUSE | Q9QZQ8 | -0.34 | -1.99 | 1.65 | 0.233758 | Yes |
| H2A1K_MOUSE | Q8CGP7 | -0.34 | -2.01 | 1.67 | 0.234641 | Yes |
| UCRI_MOUSE | Q9CR68 | 5.82 | 5.61 | 0.21 | 0.235035 | Yes |
| TOIP1_MOUSE | Q921T2 | -3.63 | -4.32 | 0.70 | 0.236666 | Yes |
| H2A1F_MOUSE | Q8CGP5 | -0.34 | -1.93 | 1.60 | 0.239705 | Yes |
| EI3JB_MOUSE | Q66JS6 | -1.39 | -2.11 | 0.72 | 0.241621 | No |
| VIGLN_MOUSE | Q8VDJ3 | -3.83 | -4.35 | 0.51 | 0.242092 | Yes |
| RT36_MOUSE | Q9CQX8 | -0.68 | 0.00 | -0.68 | 0.242558 | No |
| LDB3_MOUSE | Q9JKS4 | 6.49 | 6.19 | 0.30 | 0.243212 | Yes |
| SPCS2_MOUSE | Q9CYN2 | -0.32 | -0.67 | 0.34 | 0.243679 | Yes |
| ACYP2_MOUSE | P56375 | -2.64 | -3.62 | 0.98 | 0.245615 | Yes |
| COX1_MOUSE | P00397 | -2.95 | -1.48 | -1.47 | 0.247120 | Yes |
| ADT1_MOUSE | P48962 | 3.63 | 2.93 | 0.70 | 0.247509 | Yes |
| NDUA8_MOUSE | Q9DCJ5 | 2.87 | 1.68 | 1.20 | 0.247693 | Yes |
| DHE3_MOUSE | P26443 | -3.05 | -3.82 | 0.77 | 0.249179 | Yes |
| H2AX_MOUSE | P27661 | -0.34 | -1.85 | 1.52 | 0.249202 | Yes |
| PAIRB_MOUSE | Q9CY58 | 1.10 | 0.67 | 0.43 | 0.249606 | Yes |
| ODB2_MOUSE | P53395 | -0.33 | -1.42 | 1.09 | 0.250019 | Yes |
| COF2_MOUSE | P45591 | -0.67 | -1.43 | 0.76 | 0.250742 | Yes |
| UB2D3_MOUSE | P61079 | -3.35 | -3.99 | 0.63 | 0.251753 | Yes |
| H10_MOUSE | P10922 | 1.92 | 1.44 | 0.48 | 0.253754 | Yes |
| PARK7_MOUSE | Q99LX0 | 0.37 | -0.43 | 0.80 | 0.254019 | Yes |
| MYH3_MOUSE | P13541 | -3.48 | -4.25 | 0.77 | 0.254141 | Yes |
| ECHA_MOUSE | Q8BMS1 | 4.11 | 3.55 | 0.56 | 0.254247 | Yes |
| SERPH_MOUSE | P19324 | -3.03 | -4.11 | 1.08 | 0.254258 | Yes |
| OTUD4_MOUSE | B2RRE7 | -4.14 | -4.57 | 0.43 | 0.254545 | Yes |
| RL7A_MOUSE | P12970 | -0.38 | -1.68 | 1.29 | 0.254967 | Yes |
| FABP4_MOUSE | P04117 | -0.17 | -1.25 | 1.08 | 0.256722 | No |
| ADK_MOUSE | P55264 | 0.67 | 0.85 | -0.18 | 0.256792 | Yes |
| RS30_MOUSE | P62862 | 2.03 | 1.50 | 0.52 | 0.257389 | Yes |
| H2A2C_MOUSE | Q64523 | -1.75 | -3.28 | 1.53 | 0.258279 | Yes |
| FIS1_MOUSE | Q9CQ92 | -2.38 | -3.38 | 1.00 | 0.258792 | Yes |
| STOM_MOUSE | P54116 | -4.31 | -4.74 | 0.43 | 0.260695 | Yes |
| SLAI2_MOUSE | Q8CI08 | -4.32 | -4.79 | 0.47 | 0.261381 | Yes |
| 1433E_MOUSE | P62259 | 0.63 | -0.38 | 1.01 | 0.261537 | Yes |
| NNTM_MOUSE | Q61941 | -3.42 | -2.34 | -1.08 | 0.261655 | Yes |
| PCCB_MOUSE | Q99MN9 | -0.95 | 0.89 | -1.84 | 0.261736 | Yes |
| H2AJ_MOUSE | Q8R1M2 | -0.34 | -1.72 | 1.38 | 0.261938 | Yes |
| H31_MOUSE | P68433 | -3.83 | -4.89 | 1.06 | 0.262141 | Yes |
| BDH_MOUSE | Q80XN0 | -2.12 | -3.13 | 1.01 | 0.262794 | Yes |
| K2C6B_MOUSE | Q9Z331 | -2.76 | -3.64 | 0.88 | 0.263217 | Yes |
| CO1A2_MOUSE | Q01149 | -1.66 | -0.93 | -0.73 | 0.267152 | Yes |
| UBP2L_MOUSE | Q80X50 | 2.03 | 1.73 | 0.30 | 0.269951 | Yes |
| HEMO_MOUSE | Q91X72 | 1.37 | 2.02 | -0.65 | 0.273934 | No |
| H2A3_MOUSE | Q8BFU2 | -0.34 | -1.59 | 1.25 | 0.275507 | Yes |
| MAVS_MOUSE | Q8VCF0 | -0.95 | -1.91 | 0.97 | 0.276103 | Yes |
| ACD10_MOUSE | Q8K370 | -1.18 | -1.96 | 0.79 | 0.277346 | Yes |
| PPLA_MOUSE | P61014 | 4.07 | 4.21 | -0.14 | 0.277485 | Yes |
| PGP_MOUSE | Q8CHP8 | -2.46 | -3.27 | 0.82 | 0.277863 | Yes |
| NUCL_MOUSE | P09405 | -1.35 | -1.77 | 0.43 | 0.279401 | Yes |
| LMCD1_MOUSE | Q8VEE1 | -3.03 | -3.69 | 0.66 | 0.281241 | Yes |
| CALX_MOUSE | P35564 | -0.08 | -0.54 | 0.46 | 0.281331 | Yes |
| PRS7_MOUSE | P46471 | -1.20 | -2.01 | 0.81 | 0.287446 | Yes |
| NDUC2_MOUSE | Q9CQ54 | 2.29 | 2.01 | 0.28 | 0.288245 | Yes |
| H2A1H_MOUSE | Q8CGP6 | -0.34 | -1.48 | 1.14 | 0.288804 | Yes |
| EHD1_MOUSE | Q9WVK4 | -2.31 | -1.65 | -0.65 | 0.291062 | Yes |
| SUMO2_MOUSE | P61957 | -3.07 | -3.52 | 0.45 | 0.291384 | Yes |
| RS14_MOUSE | P62264 | -0.17 | -1.03 | 0.86 | 0.294960 | Yes |
| KV2A7_MOUSE | P01631 | 3.25 | 3.00 | 0.25 | 0.296138 | No |
| EF1A2_MOUSE | P62631 | -0.17 | -1.58 | 1.41 | 0.299250 | Yes |
| PA2G4_MOUSE | P50580 | -1.16 | -1.36 | 0.20 | 0.299262 | Yes |
| K22O_MOUSE | Q3UV17 | -0.18 | 0.86 | -1.04 | 0.301803 | Yes |
| IDHG1_MOUSE | P70404 | 0.31 | -1.04 | 1.35 | 0.302443 | Yes |
| RL37_MOUSE | Q9D823 | -0.80 | -1.80 | 1.00 | 0.304224 | Yes |
| LONM_MOUSE | Q8CGK3 | -0.05 | -0.48 | 0.43 | 0.306688 | Yes |
| SNX3_MOUSE | O70492 | 0.52 | 0.72 | -0.20 | 0.308100 | Yes |
| COQ3_MOUSE | Q8BMS4 | -0.56 | -0.75 | 0.18 | 0.308121 | Yes |
| ECI2_MOUSE | Q9WUR2 | -1.45 | -2.07 | 0.63 | 0.311851 | Yes |
| HNRPU_MOUSE | Q8VEK3 | -0.65 | -0.18 | -0.48 | 0.314142 | Yes |
| COX41_MOUSE | P19783 | 6.89 | 6.70 | 0.19 | 0.322061 | Yes |
| TM38A_MOUSE | Q3TMP8 | -2.13 | -1.66 | -0.47 | 0.323208 | Yes |
| PEBP1_MOUSE | P70296 | -0.35 | -0.91 | 0.56 | 0.323344 | Yes |
| H2A2A_MOUSE | Q6GSS7 | -1.75 | -2.74 | 0.99 | 0.324523 | No |
| CMC2_MOUSE | Q8K199 | -2.14 | -2.80 | 0.66 | 0.324687 | Yes |
| SPTB2_MOUSE | Q62261 | -0.32 | -1.06 | 0.74 | 0.330835 | Yes |
| NDUA2_MOUSE | Q9CQ75 | 2.37 | 1.75 | 0.62 | 0.331788 | Yes |
| AIMP1_MOUSE | P31230 | -1.89 | -2.49 | 0.60 | 0.332587 | Yes |
| COQ8A_MOUSE | Q60936 | -0.86 | -0.37 | -0.49 | 0.335268 | No |
| ACOT2_MOUSE | Q9QYR9 | 0.24 | -0.59 | 0.83 | 0.335429 | Yes |
| CRYAB_MOUSE | P23927 | 7.74 | 7.60 | 0.14 | 0.336613 | Yes |
| H12_MOUSE | P15864 | 4.31 | 4.04 | 0.28 | 0.338268 | Yes |
| PRDX1_MOUSE | P35700 | 2.28 | 1.76 | 0.52 | 0.338882 | Yes |
| RL7_MOUSE | P14148 | 0.36 | -0.21 | 0.57 | 0.340809 | Yes |
| IF4G1_MOUSE | Q6NZJ6 | -2.28 | -2.74 | 0.46 | 0.346955 | Yes |
| NDUS1_MOUSE | Q91VD9 | 1.91 | 1.33 | 0.57 | 0.351407 | Yes |
| NACAM_MOUSE | P70670 | 6.16 | 5.90 | 0.25 | 0.351477 | Yes |
| PRS37_MOUSE | Q9DAA4 | -4.71 | -4.33 | -0.38 | 0.351933 | No |
| CBX3_MOUSE | P23198 | -0.76 | -0.88 | 0.12 | 0.353234 | Yes |
| LAMA4_MOUSE | P97927 | -4.60 | -4.78 | 0.18 | 0.353507 | Yes |
| NDUS7_MOUSE | Q9DC70 | 2.27 | 2.01 | 0.26 | 0.355385 | Yes |
| F162A_MOUSE | Q9D6U8 | 0.08 | -0.14 | 0.22 | 0.356036 | Yes |
| IF4B_MOUSE | Q8BGD9 | -1.09 | -0.74 | -0.34 | 0.356257 | Yes |
| GLRX5_MOUSE | Q80Y14 | -1.09 | -1.32 | 0.22 | 0.360971 | No |
| ES1_MOUSE | Q9D172 | 3.15 | 2.85 | 0.31 | 0.362038 | Yes |
| ACTB_MOUSE | P60710 | -0.81 | -1.33 | 0.52 | 0.364469 | Yes |
| IVD_MOUSE | Q9JHI5 | 0.04 | 0.67 | -0.63 | 0.365866 | Yes |
| UBC12_MOUSE | P61082 | -3.07 | -3.56 | 0.49 | 0.371761 | Yes |
| H4_MOUSE | P62806 | -4.14 | -4.41 | 0.27 | 0.373474 | Yes |
| PRDX5_MOUSE | P99029 | 0.80 | 0.49 | 0.31 | 0.375147 | Yes |
| RS8_MOUSE | P62242 | -4.24 | -3.79 | -0.45 | 0.379690 | Yes |
| RAD_MOUSE | O88667 | -0.59 | -0.05 | -0.54 | 0.384515 | No |
| COX7B_MOUSE | P56393 | 3.29 | 3.08 | 0.21 | 0.385831 | No |
| ECHB_MOUSE | Q99JY0 | 5.23 | 5.11 | 0.12 | 0.385865 | Yes |
| RL29_MOUSE | P47915 | 3.11 | 2.97 | 0.15 | 0.387313 | Yes |
| RL32_MOUSE | P62911 | -0.99 | -0.38 | -0.61 | 0.395585 | Yes |
| RL23A_MOUSE | P62751 | 2.11 | 1.94 | 0.17 | 0.399848 | Yes |
| LAMC1_MOUSE | P02468 | -4.50 | -4.66 | 0.16 | 0.399952 | Yes |
| H2A2B_MOUSE | Q64522 | -1.75 | -2.25 | 0.50 | 0.403078 | Yes |
| PDK2_MOUSE | Q9JK42 | -2.39 | -2.10 | -0.29 | 0.405350 | Yes |
| BAG6_MOUSE | Q9Z1R2 | -5.04 | -4.78 | -0.26 | 0.405818 | Yes |
| CRIP2_MOUSE | Q9DCT8 | 4.88 | 4.74 | 0.14 | 0.405945 | Yes |
| GDIR1_MOUSE | Q99PT1 | 4.04 | 3.96 | 0.08 | 0.415552 | Yes |
| NAMPT_MOUSE | Q99KQ4 | -1.62 | -2.05 | 0.43 | 0.416688 | Yes |
| DBNL_MOUSE | Q62418 | -2.61 | -2.42 | -0.18 | 0.420420 | Yes |
| ACADV_MOUSE | P50544 | 4.86 | 4.79 | 0.07 | 0.421347 | Yes |
| ADX_MOUSE | P46656 | -1.60 | -1.47 | -0.13 | 0.422420 | No |
| MYH6_MOUSE | Q02566 | 4.15 | 3.98 | 0.16 | 0.423628 | Yes |
| GSTO1_MOUSE | O09131 | -1.56 | -1.65 | 0.09 | 0.427461 | Yes |
| WFS1_MOUSE | P56695 | -0.99 | -1.07 | 0.07 | 0.428518 | Yes |
| PLST_MOUSE | Q99K51 | -2.31 | -2.64 | 0.33 | 0.428989 | Yes |
| ARMC1_MOUSE | Q9D7A8 | -3.91 | -4.11 | 0.19 | 0.430819 | Yes |
| DPYL2_MOUSE | O08553 | 0.79 | 0.70 | 0.09 | 0.432733 | Yes |
| SRSF3_MOUSE | P84104 | -4.71 | -4.51 | -0.20 | 0.435436 | Yes |
| LNP_MOUSE | Q7TQ95 | -4.41 | -4.60 | 0.19 | 0.436232 | Yes |
| SDHB_MOUSE | Q9CQA3 | 4.82 | 4.76 | 0.06 | 0.440207 | Yes |
| CXA1_MOUSE | P23242 | -3.79 | -3.62 | -0.17 | 0.440470 | Yes |
| HCDH_MOUSE | Q61425 | 4.18 | 4.14 | 0.05 | 0.440940 | Yes |
| COX6C_MOUSE | Q9CPQ1 | 3.71 | 3.59 | 0.12 | 0.441764 | Yes |
| TRY2_MOUSE | P07146 | 1.48 | 1.27 | 0.21 | 0.441921 | No |
| MACF1_MOUSE | Q9QXZ0 | 1.74 | 1.81 | -0.07 | 0.443129 | Yes |
| QKI_MOUSE | Q9QYS9 | -1.89 | -1.97 | 0.08 | 0.444595 | Yes |
| CMYA5_MOUSE | Q70KF4 | -4.71 | -4.77 | 0.07 | 0.445999 | Yes |
| EGLN1_MOUSE | Q91YE3 | -3.60 | -3.74 | 0.13 | 0.449888 | Yes |
| IGKC_MOUSE | P01837 | 7.13 | 7.06 | 0.07 | 0.454883 | Yes |
| PCY1A_MOUSE | P49586 | -3.76 | -3.85 | 0.09 | 0.459123 | Yes |
| NDUAC_MOUSE | Q7TMF3 | 3.83 | 3.80 | 0.03 | 0.466698 | Yes |
| PIN4_MOUSE | Q9CWW6 | -4.52 | -4.45 | -0.07 | 0.469199 | Yes |
| FRIH_MOUSE | P09528 | 2.35 | 2.33 | 0.02 | 0.470345 | Yes |
| IF4H_MOUSE | Q9WUK2 | 0.92 | 0.86 | 0.06 | 0.470791 | Yes |
| SLK_MOUSE | O54988 | -5.02 | -5.07 | 0.05 | 0.472488 | Yes |
| EFHD2_MOUSE | Q9D8Y0 | -0.58 | -0.67 | 0.09 | 0.472830 | Yes |
| NP1L4_MOUSE | Q78ZA7 | -1.79 | -1.73 | -0.06 | 0.473383 | Yes |
| QCR6_MOUSE | P99028 | 2.48 | 2.54 | -0.06 | 0.473449 | No |
| BAG3_MOUSE | Q9JLV1 | 1.11 | 1.00 | 0.11 | 0.476326 | Yes |
| KAD3_MOUSE | Q9WTP7 | -4.77 | -4.84 | 0.07 | 0.477792 | Yes |
| MYH11_MOUSE | O08638 | -3.92 | -3.87 | -0.05 | 0.478741 | Yes |
| CISY_MOUSE | Q9CZU6 | 4.96 | 4.93 | 0.03 | 0.479899 | Yes |
| TRDN_MOUSE | E9Q9K5 | -3.61 | -3.57 | -0.04 | 0.485630 | No |
| ODO1_MOUSE | Q60597 | 2.77 | 2.79 | -0.02 | 0.487520 | No |
| THIKA_MOUSE | Q921H8 | -3.90 | -3.93 | 0.03 | 0.489509 | No |
| NSF1C_MOUSE | Q9CZ44 | 0.66 | 0.69 | -0.02 | 0.489865 | Yes |
| CO6A1_MOUSE | Q04857 | -5.78 | -5.76 | -0.01 | 0.491049 | Yes |
| TAGL_MOUSE | P37804 | 1.18 | 1.19 | -0.01 | 0.495353 | Yes |
| PROSC_MOUSE | Q9Z2Y8 | -0.16 | -0.14 | -0.01 | 0.496363 | No |
| RL35_MOUSE | Q6ZWV7 | -3.89 | -3.90 | 0.01 | 0.496787 | Yes |
| SLIRP_MOUSE | Q9D8T7 | 0.08 | 0.08 | 0.00 | 0.498050 | Yes |
| RS24_MOUSE | P62849 | -1.99 | -1.98 | -0.01 | 0.498152 | Yes |
| IF4A3_MOUSE | Q91VC3 | -4.14 | -4.14 | 0.00 | 0.499981 | Yes |
