# Supplementary material for: First comprehensive identification of cardiac proteins with putative increased O-GlcNAc levels during pressure overload hypertrophy
Source: PLoS One. 2022 Oct 26;17(10):e0276285. doi: 10.1371/journal.pone.0276285 (PMC9605332; doi:10.1371/journal.pone.0276285)
Supplement: S2 Table — (DOCX) [file pone.0276285.s002.docx]

**S2 Table. Proteins with significant changes in putative O-GlcNAc levels in pressure overload hypertrophy (POH) versus Sham**

| **Accession** | **Uniprot accession number** | **Description (Common Name)** | **average-POH**  **n=5** | **average-sham**  **n=5** | **log2 Fold change POH-Sham** | **p-value** |
| --- | --- | --- | --- | --- | --- | --- |
| RSSA_MOUSE | P14206 | 40S ribosomal protein SA | -1.52 | -5.15 | 3.63 | 0.00004 |
| RANG_MOUSE | P34022 | Ran-specific GTPase-activating protein | -2.91 | -5.61 | 2.71 | 0.00042 |
| ERP44_MOUSE | Q9D1Q6 | Endoplasmic reticulum resident protein 44 | -2.80 | -5.25 | 2.45 | 0.00049 |
| NHRF2_MOUSE | Q9JHL1 | Na(+)/H(+) exchange regulatory cofactor NHE-RF2 | -1.48 | -4.92 | 3.44 | 0.00059 |
| PTN11_MOUSE | P35235 | Tyrosine-protein phosphatase non-receptor type 11 | -2.53 | -5.12 | 2.59 | 0.00062 |
| ATPF1_MOUSE | Q811I0 | ATP synthase mitochondrial F1 complex assembly factor 1 | -2.75 | -5.24 | 2.49 | 0.00068 |
| SEPT8_MOUSE | Q8CHH9 | Septin-8 | -2.89 | -5.38 | 2.49 | 0.00103 |
| IGJ_MOUSE | P01592 | Immunoglobulin J chain | -1.09 | -5.60 | 4.51 | 0.00112 |
| GRP78_MOUSE | P20029 | 78 kDa glucose-regulated protein | 2.30 | -2.26 | 4.56 | 0.00116 |
| TOM1_MOUSE | O88746 | Target of Myb protein 1 | -0.90 | -3.78 | 2.88 | 0.00161 |
| RL26_MOUSE | P61255 | 60S ribosomal protein L26 | -1.82 | -5.21 | 3.39 | 0.00178 |
| PAK2_MOUSE | Q8CIN4 | Serine/threonine-protein kinase PAK 2 | -2.24 | -4.25 | 2.02 | 0.00203 |
| PP14C_MOUSE | Q8R4S0 | Protein phosphatase 1 regulatory subunit 14C | -1.88 | -4.43 | 2.55 | 0.00265 |
| SRA1_MOUSE | Q80VJ2 | Steroid receptor RNA activator 1 | -2.70 | -5.39 | 2.69 | 0.00277 |
| G3BP1_MOUSE | P97855 | Ras GTPase-activating protein-binding protein 1 | -0.96 | -5.02 | 4.06 | 0.00299 |
| GELS_MOUSE | P13020 | Gelsolin | -3.25 | -5.38 | 2.13 | 0.00344 |
| NEXN_MOUSE | Q7TPW1 | Nexilin | -3.47 | -5.53 | 2.06 | 0.00374 |
| CPZIP_MOUSE | Q3UZA1 | CapZ-interacting protein | -2.04 | -5.02 | 2.99 | 0.00384 |
| GSTA4_MOUSE | P24472 | Glutathione S-transferase A4 | -2.64 | -5.17 | 2.53 | 0.00388 |
| CPT1B_MOUSE | Q924X2 | Carnitine O-palmitoyltransferase 1 muscle isoform | -1.24 | -4.68 | 3.45 | 0.00402 |
| MIC25_MOUSE | Q91VN4 | MICOS complex subunit Mic25 | -2.31 | -5.00 | 2.69 | 0.00406 |
| HNRPQ_MOUSE | Q7TMK9 | Heterogeneous nuclear ribonucleoprotein Q | -2.98 | -5.12 | 2.14 | 0.00418 |
| NDUS2_MOUSE | Q91WD5 | NADH dehydrogenase [ubiquinone] iron-sulfur protein 2 mitochondrial | -2.99 | -4.92 | 1.93 | 0.00430 |
| PRDX6_MOUSE | O08709 | Peroxiredoxin-6 | 0.00 | -3.55 | 3.54 | 0.00458 |
| SYP2L_MOUSE | Q8BWB1 | Synaptopodin 2-like protein | -1.82 | -5.27 | 3.45 | 0.00502 |
| CAH2_MOUSE | P00920 | Carbonic anhydrase 2 | -2.07 | -4.56 | 2.48 | 0.00503 |
| G6PI_MOUSE | P06745 | Glucose-6-phosphate isomerase | 1.70 | -2.59 | 4.29 | 0.00509 |
| AFG32_MOUSE | Q8JZQ2 | AFG3-like protein 2 | -2.95 | -5.36 | 2.41 | 0.00518 |
| MIC27_MOUSE | Q78IK4 | MICOS complex subunit Mic27 | -1.11 | -4.05 | 2.94 | 0.00524 |
| IF5A1_MOUSE | P63242 | Eukaryotic translation initiation factor 5A-1 | -2.02 | -5.30 | 3.29 | 0.00539 |
| RS18_MOUSE | P62270 | 40S ribosomal protein S18 | -2.57 | -5.16 | 2.59 | 0.00541 |
| TIM44_MOUSE | O35857 | Mitochondrial import inner membrane translocase subunit TIM44 | -2.63 | -5.35 | 2.72 | 0.00573 |
| TRFE_MOUSE | Q921I1 | Serotransferrin | 0.00 | -4.14 | 4.13 | 0.00589 |
| TBA4A_MOUSE | P68368 | Tubulin alpha-4A chain | -2.67 | -4.82 | 2.15 | 0.00650 |
| G3BP2_MOUSE | P97379 | Ras GTPase-activating protein-binding protein 2 | -2.43 | -4.99 | 2.55 | 0.00672 |
| FBN1_MOUSE | Q61554 | Fibrillin-1 | -1.48 | -4.91 | 3.43 | 0.00677 |
| GDIB_MOUSE | Q61598 | Rab GDP dissociation inhibitor beta | -2.39 | -5.44 | 3.05 | 0.00678 |
| MTCH2_MOUSE | Q791V5 | Mitochondrial carrier homolog 2 | -2.56 | -4.88 | 2.32 | 0.00686 |
| CAV1_MOUSE | P49817 | Caveolin-1 | -1.22 | -4.38 | 3.16 | 0.00728 |
| MYOZ2_MOUSE | Q9JJW5 | Myozenin-2 | -0.72 | -3.84 | 3.12 | 0.00733 |
| MOES_MOUSE | P26041 | Moesin | 2.51 | 1.01 | 1.51 | 0.00737 |
| DEST_MOUSE | Q9R0P5 | Destrin | -2.19 | -4.87 | 2.68 | 0.00760 |
| GPSM1_MOUSE | Q6IR34 | G-protein-signaling modulator 1 | -3.52 | -5.61 | 2.09 | 0.00788 |
| UB2L3_MOUSE | P68037 | Ubiquitin-conjugating enzyme E2 L3 | -1.83 | -4.10 | 2.27 | 0.00792 |
| ITB1_MOUSE | P09055 | Integrin beta-1 | -2.82 | -4.97 | 2.15 | 0.00799 |
| ROA3_MOUSE | Q8BG05 | Heterogeneous nuclear ribonucleoprotein A3 | 0.06 | -3.31 | 3.37 | 0.00802 |
| RADI_MOUSE | P26043 | Radixin | -3.04 | -5.64 | 2.60 | 0.00805 |
| MLIP_MOUSE | Q5FW52 | Muscular LMNA-interacting protein | -2.13 | -5.30 | 3.17 | 0.00809 |
| CYB5_MOUSE | P56395 | Cytochrome b5 | -1.31 | -4.61 | 3.29 | 0.00811 |
| TGM2_MOUSE | P21981 | Protein-glutamine gamma-glutamyltransferase 2 | -1.69 | -4.83 | 3.14 | 0.00818 |
| PGAM1_MOUSE | Q9DBJ1 | Phosphoglycerate mutase 1 | -0.96 | -3.63 | 2.67 | 0.00830 |
| RL30_MOUSE | P62889 | 60S ribosomal protein L30 | 0.11 | -0.62 | 0.73 | 0.00847 |
| RAN_MOUSE | P62827 | GTP-binding nuclear protein Ran | -1.68 | -4.33 | 2.65 | 0.00855 |
| MIMIT_MOUSE | Q59J78 | Mimitin mitochondrial | -1.32 | -4.12 | 2.80 | 0.00866 |
| FKBP3_MOUSE | Q62446 | Peptidyl-prolyl cis-trans isomerase FKBP3 | -2.04 | -5.36 | 3.32 | 0.00889 |
| SPTN1_MOUSE | P16546 | Spectrin alpha chain non-erythrocytic 1 | -2.88 | -5.41 | 2.53 | 0.00923 |
| PABP2_MOUSE | Q8CCS6 | Polyadenylate-binding protein 2 | -3.42 | -5.49 | 2.07 | 0.00939 |
| PCCA_MOUSE | Q91ZA3 | Propionyl-CoA carboxylase alpha chain mitochondrial | -1.34 | -4.87 | 3.52 | 0.00972 |
| DCTN2_MOUSE | Q99KJ8 | Dynactin subunit 2 | -1.87 | -5.32 | 3.45 | 0.00986 |
| EIF3B_MOUSE | Q8JZQ9 | Eukaryotic translation initiation factor 3 subunit B | -1.45 | -4.65 | 3.20 | 0.01064 |
| SFPQ_MOUSE | Q8VIJ6 | Splicing factor proline- and glutamine-rich | -1.03 | -5.35 | 4.32 | 0.01089 |
| NDUV1_MOUSE | Q91YT0 | NADH dehydrogenase [ubiquinone] flavoprotein 1 mitochondrial | 0.03 | -3.63 | 3.66 | 0.01105 |
| AK1A1_MOUSE | Q9JII6 | Alcohol dehydrogenase [NADP(+)] | -2.75 | -4.77 | 2.02 | 0.01118 |
| ICAL_MOUSE | P51125 | Calpastatin | -1.58 | -5.03 | 3.45 | 0.01134 |
| YBOX1_MOUSE | P62960 | Nuclease-sensitive element-binding protein 1 | -2.45 | -4.63 | 2.19 | 0.01155 |
| PRC2C_MOUSE | Q3TLH4 | Protein PRRC2C | -3.10 | -5.46 | 2.36 | 0.01163 |
| SGCA_MOUSE | P82350 | Alpha-sarcoglycan | -3.09 | -5.62 | 2.53 | 0.01205 |
| COX5A_MOUSE | P12787 | Cytochrome c oxidase subunit 5A mitochondrial | 4.47 | 1.73 | 2.74 | 0.01207 |
| GRPE1_MOUSE | Q99LP6 | GrpE protein homolog 1 mitochondrial | -1.71 | -5.22 | 3.51 | 0.01217 |
| L2HDH_MOUSE | Q91YP0 | L-2-hydroxyglutarate dehydrogenase mitochondrial | -1.53 | -4.78 | 3.24 | 0.01222 |
| RS4X_MOUSE | P62702 | 40S ribosomal protein S4 X isoform | -2.26 | -5.10 | 2.84 | 0.01224 |
| TKT_MOUSE | P40142 | Transketolase | -2.71 | -5.20 | 2.48 | 0.01243 |
| PDLI1_MOUSE | O70400 | PDZ and LIM domain protein 1 | 1.97 | -0.12 | 2.09 | 0.01256 |
| RT02_MOUSE | Q924T2 | 28S ribosomal protein S2 mitochondrial | -2.94 | -4.68 | 1.74 | 0.01276 |
| STIP1_MOUSE | Q60864 | Stress-induced-phosphoprotein 1 | -1.19 | -4.76 | 3.57 | 0.01311 |
| HP1B3_MOUSE | Q3TEA8 | Heterochromatin protein 1-binding protein 3 | -2.80 | -5.67 | 2.88 | 0.01311 |
| TTHY_MOUSE | P07309 | Transthyretin | -1.53 | -4.58 | 3.05 | 0.01356 |
| B2MG_MOUSE | P01887 | Beta-2-microglobulin | -1.43 | -3.77 | 2.34 | 0.01368 |
| RS7_MOUSE | P62082 | 40S ribosomal protein S7 | -2.80 | -4.86 | 2.06 | 0.01377 |
| EH1L1_MOUSE | Q99MS7 | EH domain-binding protein 1-like protein 1 | -2.85 | -5.14 | 2.30 | 0.01390 |
| PGAM2_MOUSE | O70250 | Phosphoglycerate mutase 2 | 3.45 | 1.57 | 1.89 | 0.01437 |
| NOL3_MOUSE | Q9D1X0 | Nucleolar protein 3 | -3.25 | -5.12 | 1.87 | 0.01439 |
| ANXA7_MOUSE | Q07076 | Annexin A7 | -2.61 | -5.20 | 2.59 | 0.01501 |
| HSPB1_MOUSE | P14602 | Heat shock protein beta-1 | 5.17 | 4.02 | 1.15 | 0.01505 |
| CTNA1_MOUSE | P26231 | Catenin alpha-1 | -3.04 | -4.88 | 1.84 | 0.01509 |
| LASP1_MOUSE | Q61792 | LIM and SH3 domain protein 1 | -2.08 | -4.60 | 2.52 | 0.01563 |
| ALDH2_MOUSE | P47738 | Aldehyde dehydrogenase mitochondrial | 0.18 | -2.73 | 2.91 | 0.01564 |
| CAP1_MOUSE | P40124 | Adenylyl cyclase-associated protein 1 | -2.58 | -4.68 | 2.10 | 0.01566 |
| ACADM_MOUSE | P45952 | Medium-chain specific acyl-CoA dehydrogenase mitochondrial | 3.69 | 1.77 | 1.92 | 0.01574 |
| CYTC_MOUSE | P21460 | Cystatin-C | -3.86 | -5.92 | 2.06 | 0.01618 |
| CO3_MOUSE | P01027 | Complement C3 | -2.58 | -5.66 | 3.08 | 0.01639 |
| RL12_MOUSE | P35979 | 60S ribosomal protein L12 | -1.82 | -5.38 | 3.56 | 0.01668 |
| LAMA2_MOUSE | Q60675 | Laminin subunit alpha-2 | -3.50 | -5.68 | 2.18 | 0.01696 |
| LC7L2_MOUSE | Q7TNC4 | Putative RNA-binding protein Luc7-like 2 | -3.16 | -5.01 | 1.85 | 0.01698 |
| B2L13_MOUSE | P59017 | Bcl-2-like protein 13 | -2.38 | -5.41 | 3.03 | 0.01713 |
| GCAB_MOUSE | P01864 | Ig gamma-2A chain C region secreted form | -1.27 | -4.31 | 3.04 | 0.01713 |
| VIME_MOUSE | P20152 | Vimentin | 1.81 | -1.22 | 3.03 | 0.01756 |
| RT33_MOUSE | Q9D2R8 | 28S ribosomal protein S33 mitochondrial | -2.60 | -4.88 | 2.27 | 0.01790 |
| NDUB3_MOUSE | Q9CQZ6 | NADH dehydrogenase [ubiquinone] 1 beta subcomplex subunit 3 | 1.00 | -3.72 | 4.72 | 0.01805 |
| CLIP1_MOUSE | Q922J3 | CAP-Gly domain-containing linker protein 1 | -2.26 | -5.71 | 3.45 | 0.01824 |
| TRI72_MOUSE | Q1XH17 | Tripartite motif-containing protein 72 | 0.99 | -0.78 | 1.77 | 0.01837 |
| ACTN2_MOUSE | Q9JI91 | Alpha-actinin-2 | -1.30 | -4.25 | 2.95 | 0.01973 |
| SNRPA_MOUSE | Q62189 | U1 small nuclear ribonucleoprotein A | -1.72 | -4.19 | 2.46 | 0.02021 |
| NEBL_MOUSE | Q0II04 | Nebulette | -2.07 | -4.30 | 2.23 | 0.02022 |
| PLIN4_MOUSE | O88492 | Perilipin-4 | -1.06 | -3.19 | 2.13 | 0.02033 |
| CLAP1_MOUSE | Q80TV8 | CLIP-associating protein 1 | -3.11 | -4.84 | 1.73 | 0.02036 |
| THIL_MOUSE | Q8QZT1 | Acetyl-CoA acetyltransferase mitochondrial | 4.20 | 2.24 | 1.96 | 0.02037 |
| AKAP2_MOUSE | O54931 | A-kinase anchor protein 2 | -1.72 | -3.65 | 1.94 | 0.02053 |
| QCR7_MOUSE | Q9D855 | Cytochrome b-c1 complex subunit 7 | 1.53 | -3.32 | 4.85 | 0.02070 |
| REEP5_MOUSE | Q60870 | Receptor expression-enhancing protein 5 | -1.77 | -3.96 | 2.19 | 0.02095 |
| RL13A_MOUSE | P19253 | 60S ribosomal protein L13a | -2.62 | -5.30 | 2.68 | 0.02096 |
| DLRB1_MOUSE | P62627 | Dynein light chain roadblock-type 1 | -2.45 | -5.41 | 2.97 | 0.02108 |
| NDUS8_MOUSE | Q8K3J1 | NADH dehydrogenase [ubiquinone] iron-sulfur protein 8 mitochondrial | 0.80 | -1.74 | 2.54 | 0.02131 |
| PSMD9_MOUSE | Q9CR00 | 26S proteasome non-ATPase regulatory subunit 9 | -1.86 | -4.09 | 2.23 | 0.02136 |
| PALMD_MOUSE | Q9JHU2 | Palmdelphin | -2.71 | -4.78 | 2.06 | 0.02167 |
| AIFM1_MOUSE | Q9Z0X1 | Apoptosis-inducing factor 1 mitochondrial | 1.05 | -0.94 | 1.99 | 0.02187 |
| HSPB7_MOUSE | P35385 | Heat shock protein beta-7 | -1.54 | -4.28 | 2.74 | 0.02205 |
| ROAA_MOUSE | Q99020 | Heterogeneous nuclear ribonucleoprotein A/B | -2.98 | -5.03 | 2.05 | 0.02207 |
| EF1D_MOUSE | P57776 | Elongation factor 1-delta | 1.36 | -3.09 | 4.44 | 0.02217 |
| PACN3_MOUSE | Q99JB8 | Protein kinase C and casein kinase II substrate protein 3 | -1.52 | -3.54 | 2.02 | 0.02221 |
| 1433T_MOUSE | P68254 | 14-3-3 protein theta | -0.78 | -4.36 | 3.58 | 0.02225 |
| DLDH_MOUSE | O08749 | Dihydrolipoyl dehydrogenase mitochondrial | 2.76 | 1.24 | 1.52 | 0.02243 |
| COF1_MOUSE | P18760 | Cofilin-1 | 0.24 | -0.58 | 0.82 | 0.02244 |
| MYH7_MOUSE | Q91Z83 | Myosin-7 | -1.82 | -4.52 | 2.70 | 0.02269 |
| MACD1_MOUSE | Q922B1 | O-acetyl-ADP-ribose deacetylase MACROD1 | -0.33 | -4.12 | 3.79 | 0.02274 |
| RS6_MOUSE | P62754 | 40S ribosomal protein S6 | -1.69 | -4.02 | 2.33 | 0.02287 |
| VAPB_MOUSE | Q9QY76 | Vesicle-associated membrane protein-associated protein B | -0.39 | -2.00 | 1.61 | 0.02300 |
| FBLN2_MOUSE | P37889 | Fibulin-2 | -2.07 | -4.99 | 2.92 | 0.02353 |
| ALDOA_MOUSE | P05064 | Fructose-bisphosphate aldolase A | 4.53 | 1.55 | 2.97 | 0.02354 |
| SODM_MOUSE | P09671 | Superoxide dismutase [Mn] mitochondrial | -1.10 | -3.68 | 2.58 | 0.02372 |
| CHCH2_MOUSE | Q9D1L0 | Coiled-coil-helix-coiled-coil-helix domain-containing protein 2 | -0.43 | -2.55 | 2.13 | 0.02390 |
| CD34_MOUSE | Q64314 | Hematopoietic progenitor cell antigen CD34 | -2.25 | -4.86 | 2.61 | 0.02400 |
| MDHM_MOUSE | P08249 | Malate dehydrogenase mitochondrial | 6.35 | 4.94 | 1.41 | 0.02418 |
| NDUV2_MOUSE | Q9D6J6 | NADH dehydrogenase [ubiquinone] flavoprotein 2 mitochondrial | 0.30 | -3.28 | 3.58 | 0.02418 |
| NIPS2_MOUSE | O55126 | Protein NipSnap homolog 2 | 3.06 | 2.31 | 0.76 | 0.02457 |
| ATP5L_MOUSE | Q9CPQ8 | ATP synthase subunit g mitochondrial | -3.45 | -5.46 | 2.01 | 0.02516 |
| 68MP_MOUSE | P56379 | 6.8 kDa mitochondrial proteolipid | -2.30 | -4.69 | 2.38 | 0.02539 |
| TM1L2_MOUSE | Q5SRX1 | TOM1-like protein 2 | -3.05 | -4.99 | 1.94 | 0.02557 |
| NDUBA_MOUSE | Q9DCS9 | NADH dehydrogenase [ubiquinone] 1 beta subcomplex subunit 10 | 3.41 | 1.58 | 1.83 | 0.02557 |
| FLNA_MOUSE | Q8BTM8 | Filamin-A | 0.59 | -2.46 | 3.05 | 0.02562 |
| CMC1_MOUSE | Q9CPZ8 | Calcium-binding mitochondrial carrier protein Aralar1 | -2.06 | -4.30 | 2.24 | 0.02564 |
| RRBP1_MOUSE | Q99PL5 | Ribosome-binding protein 1 | 0.40 | -1.23 | 1.64 | 0.02589 |
| NDUBB_MOUSE | O09111 | NADH dehydrogenase [ubiquinone] 1 beta subcomplex subunit 11 mitochondrial | 4.76 | 3.97 | 0.79 | 0.02594 |
| FUMH_MOUSE | P97807 | Fumarate hydratase mitochondrial | 2.20 | -1.83 | 4.03 | 0.02620 |
| RS12_MOUSE | P63323 | 40S ribosomal protein S12 | -3.73 | -5.66 | 1.92 | 0.02656 |
| AT1A2_MOUSE | Q6PIE5 | Sodium/potassium-transporting ATPase subunit alpha-2 | -1.14 | -3.84 | 2.70 | 0.02717 |
| GRHPR_MOUSE | Q91Z53 | Glyoxylate reductase/hydroxypyruvate reductase | -1.70 | -5.18 | 3.48 | 0.02751 |
| KNG1_MOUSE | O08677 | Kininogen-1 | -0.48 | -2.63 | 2.15 | 0.02769 |
| RS21_MOUSE | Q9CQR2 | 40S ribosomal protein S21 | -1.40 | -3.81 | 2.41 | 0.02786 |
| FUBP2_MOUSE | Q3U0V1 | Far upstream element-binding protein 2 | -4.23 | -5.47 | 1.24 | 0.02822 |
| CCHL_MOUSE | P53702 | Cytochrome c-type heme lyase | -2.51 | -4.57 | 2.06 | 0.02863 |
| 1433Z_MOUSE | P63101 | 14-3-3 protein zeta/delta | 2.32 | 0.77 | 1.55 | 0.02879 |
| RL3_MOUSE | P27659 | 60S ribosomal protein L3 | -3.96 | -4.98 | 1.02 | 0.02949 |
| PGK1_MOUSE | P09411 | Phosphoglycerate kinase 1 | 2.58 | 1.57 | 1.01 | 0.02954 |
| SPRE_MOUSE | Q64105 | Sepiapterin reductase | -0.87 | -3.11 | 2.23 | 0.02962 |
| ANT3_MOUSE | P32261 | Antithrombin-III | -1.28 | -4.71 | 3.43 | 0.02979 |
| VINC_MOUSE | Q64727 | Vinculin | 0.20 | -2.73 | 2.92 | 0.03064 |
| UBA1_MOUSE | Q02053 | Ubiquitin-like modifier-activating enzyme 1 | -2.45 | -4.19 | 1.74 | 0.03077 |
| FLNC_MOUSE | Q8VHX6 | Filamin-C | 0.27 | -1.89 | 2.17 | 0.03094 |
| COX2_MOUSE | P00405 | Cytochrome c oxidase subunit 2 | 2.78 | 0.35 | 2.42 | 0.03109 |
| XIRP1_MOUSE | O70373 | Xin actin-binding repeat-containing protein 1 | -1.42 | -3.99 | 2.56 | 0.03113 |
| QCR9_MOUSE | Q8R1I1 | Cytochrome b-c1 complex subunit 9 | -1.42 | -3.94 | 2.52 | 0.03161 |
| THTM_MOUSE | Q99J99 | 3-mercaptopyruvate sulfurtransferase | -2.95 | -4.64 | 1.69 | 0.03175 |
| DC1I2_MOUSE | O88487 | Cytoplasmic dynein 1 intermediate chain 2 | -1.29 | -3.84 | 2.55 | 0.03180 |
| SGCD_MOUSE | P82347 | Delta-sarcoglycan | -3.01 | -4.94 | 1.94 | 0.03190 |
| SUCB2_MOUSE | Q9Z2I8 | Succinate--CoA ligase [GDP-forming] subunit beta mitochondrial | -1.46 | -3.81 | 2.35 | 0.03221 |
| DHB8_MOUSE | P50171 | Estradiol 17-beta-dehydrogenase 8 | -1.75 | -4.62 | 2.87 | 0.03223 |
| COX5B_MOUSE | P19536 | Cytochrome c oxidase subunit 5B mitochondrial | 6.79 | 5.43 | 1.36 | 0.03257 |
| APOE_MOUSE | P08226 | Apolipoprotein E | -2.28 | -4.84 | 2.56 | 0.03278 |
| ANF_MOUSE | P05125 | Natriuretic peptides A | 0.28 | -3.72 | 3.99 | 0.03295 |
| PIMT_MOUSE | P23506 | Protein-L-isoaspartate(D-aspartate) O-methyltransferase | -0.21 | -2.07 | 1.86 | 0.03302 |
| ATPK_MOUSE | P56135 | ATP synthase subunit f mitochondrial | 4.31 | 3.40 | 0.90 | 0.03324 |
| BASI_MOUSE | P18572 | Basigin | -0.22 | -3.17 | 2.95 | 0.03383 |
| PSA1_MOUSE | Q9R1P4 | Proteasome subunit alpha type-1 | -1.02 | -4.63 | 3.61 | 0.03389 |
| OPA1_MOUSE | P58281 | Dynamin-like 120 kDa protein mitochondrial | -1.20 | -2.85 | 1.66 | 0.03439 |
| ANXA5_MOUSE | P48036 | Annexin A5 | -1.06 | -3.54 | 2.48 | 0.03450 |
| PABP1_MOUSE | P29341 | Polyadenylate-binding protein 1 | -3.38 | -5.09 | 1.71 | 0.03508 |
| LMNA_MOUSE | P48678 | Prelamin-A/C | -3.02 | -5.31 | 2.29 | 0.03560 |
| ALBU_MOUSE | P07724 | Serum albumin | 5.20 | 2.89 | 2.31 | 0.03561 |
| ACADS_MOUSE | Q07417 | Short-chain specific acyl-CoA dehydrogenase mitochondrial | -0.62 | -3.51 | 2.89 | 0.03610 |
| MIC19_MOUSE | Q9CRB9 | MICOS complex subunit Mic19 | 3.07 | 1.19 | 1.87 | 0.03629 |
| HXK1_MOUSE | P17710 | Hexokinase-1 | -0.82 | -2.40 | 1.59 | 0.03633 |
| RTN4_MOUSE | Q99P72 | Reticulon-4 | -1.91 | -4.36 | 2.45 | 0.03633 |
| PGM1_MOUSE | Q9D0F9 | Phosphoglucomutase-1 | -3.00 | -4.67 | 1.67 | 0.03636 |
| USO1_MOUSE | Q9Z1Z0 | General vesicular transport factor p115 | -2.46 | -4.48 | 2.03 | 0.03646 |
| ODP2_MOUSE | Q8BMF4 | Dihydrolipoyllysine-residue acetyltransferase component of pyruvate dehydrogenase complex mitochondrial | 4.55 | 3.21 | 1.33 | 0.03662 |
| UBQL2_MOUSE | Q9QZM0 | Ubiquilin-2 | -3.51 | -5.74 | 2.23 | 0.03672 |
| GLYG_MOUSE | Q9R062 | Glycogenin-1 | -0.55 | -3.66 | 3.11 | 0.03701 |
| MYH9_MOUSE | Q8VDD5 | Myosin-9 | -2.63 | -5.01 | 2.39 | 0.03729 |
| RS2_MOUSE | P25444 | 40S ribosomal protein S2 | -2.09 | -4.28 | 2.19 | 0.03750 |
| TELO2_MOUSE | Q9DC40 | Telomere length regulation protein TEL2 homolog | 4.76 | 3.30 | 1.46 | 0.03761 |
| DESM_MOUSE | P31001 | Desmin | -2.08 | -4.44 | 2.36 | 0.03808 |
| RS15_MOUSE | P62843 | 40S ribosomal protein S15 | -1.58 | -2.86 | 1.29 | 0.03825 |
| TAU_MOUSE | P10637 | Microtubule-associated protein tau | -0.50 | -2.98 | 2.48 | 0.03833 |
| ANXA2_MOUSE | P07356 | Annexin A2 | -2.79 | -4.69 | 1.90 | 0.03834 |
| ACTN4_MOUSE | P57780 | Alpha-actinin-4 | -3.97 | -4.95 | 0.99 | 0.03839 |
| LAMB2_MOUSE | Q61292 | Laminin subunit beta-2 | -4.08 | -5.57 | 1.49 | 0.03851 |
| B4GT1_MOUSE | P15535 | Beta-1 4-galactosyltransferase 1 | 2.26 | -0.59 | 2.85 | 0.03944 |
| HS90B_MOUSE | P11499 | Heat shock protein HSP 90-beta | 1.88 | -0.09 | 1.98 | 0.03968 |
| ATPB_MOUSE | P56480 | ATP synthase subunit beta mitochondrial | 8.22 | 7.26 | 0.96 | 0.03984 |
| PZP_MOUSE | Q61838 | Pregnancy zone protein | -0.39 | -3.36 | 2.97 | 0.04003 |
| DBLOH_MOUSE | Q9JIQ3 | Diablo homolog mitochondrial | -3.15 | -4.80 | 1.64 | 0.04016 |
| NDUB8_MOUSE | Q9D6J5 | NADH dehydrogenase [ubiquinone] 1 beta subcomplex subunit 8 mitochondrial | 4.26 | 3.42 | 0.85 | 0.04016 |
| HINT1_MOUSE | P70349 | Histidine triad nucleotide-binding protein 1 | 2.91 | 1.38 | 1.53 | 0.04064 |
| LETM1_MOUSE | Q9Z2I0 | LETM1 and EF-hand domain-containing protein 1 mitochondrial | -0.10 | -2.19 | 2.10 | 0.04112 |
| RS3_MOUSE | P62908 | 40S ribosomal protein S3 | 1.15 | 0.24 | 0.91 | 0.04137 |
| CLH1_MOUSE | Q68FD5 | Clathrin heavy chain 1 | -3.21 | -5.10 | 1.90 | 0.04141 |
| COQ9_MOUSE | Q8K1Z0 | Ubiquinone biosynthesis protein COQ9 mitochondrial | 1.21 | -2.53 | 3.74 | 0.04148 |
| FRIL1_MOUSE | P29391 | Ferritin light chain 1 | -0.30 | -3.05 | 2.75 | 0.04192 |
| KINH_MOUSE | Q61768 | Kinesin-1 heavy chain | -0.73 | -3.14 | 2.41 | 0.04211 |
| ACADL_MOUSE | P51174 | Long-chain specific acyl-CoA dehydrogenase mitochondrial | 5.22 | 4.51 | 0.70 | 0.04222 |
| NDUS5_MOUSE | Q99LY9 | NADH dehydrogenase [ubiquinone] iron-sulfur protein 5 | -0.44 | -3.90 | 3.46 | 0.04254 |
| DECR_MOUSE | Q9CQ62 | 2 4-dienoyl-CoA reductase mitochondrial | 1.59 | 0.10 | 1.48 | 0.04314 |
| GNAS2_MOUSE | P63094 | Guanine nucleotide-binding protein G(s) subunit alpha isoforms short | -3.37 | -4.96 | 1.59 | 0.04330 |
| UBE2N_MOUSE | P61089 | Ubiquitin-conjugating enzyme E2 N | -2.22 | -4.83 | 2.60 | 0.04336 |
| AKAP1_MOUSE | O08715 | A-kinase anchor protein 1 mitochondrial | -2.66 | -4.39 | 1.72 | 0.04363 |
| MIC60_MOUSE | Q8CAQ8 | MICOS complex subunit Mic60 | 2.39 | -0.90 | 3.29 | 0.04402 |
| DC1L1_MOUSE | Q8R1Q8 | Cytoplasmic dynein 1 light intermediate chain 1 | -2.13 | -4.69 | 2.56 | 0.04459 |
| KAD2_MOUSE | Q9WTP6 | Adenylate kinase 2 mitochondrial | 1.87 | 0.18 | 1.69 | 0.04466 |
| PDIA1_MOUSE | P09103 | Protein disulfide-isomerase | -1.87 | -4.26 | 2.39 | 0.04484 |
| AATC_MOUSE | P05201 | Aspartate aminotransferase cytoplasmic | 2.02 | 0.01 | 2.01 | 0.04554 |
| H15_MOUSE | P43276 | Histone H1.5 | 1.54 | -1.12 | 2.65 | 0.04558 |
| PICAL_MOUSE | Q7M6Y3 | Phosphatidylinositol-binding clathrin assembly protein | -1.56 | -4.25 | 2.69 | 0.04650 |
| MIC26_MOUSE | Q9DCZ4 | MICOS complex subunit Mic26 | -0.36 | -3.38 | 3.02 | 0.04664 |
| ANXA1_MOUSE | P10107 | Annexin A1 | -1.96 | -4.04 | 2.09 | 0.04691 |
| PCBP1_MOUSE | P60335 | Poly(rC)-binding protein 1 | -3.66 | -4.76 | 1.10 | 0.04729 |
| RL4_MOUSE | Q9D8E6 | 60S ribosomal protein L4 | -1.58 | -4.48 | 2.90 | 0.04751 |
| DNJA3_MOUSE | Q99M87 | DnaJ homolog subfamily A member 3 mitochondrial | -3.99 | -5.08 | 1.09 | 0.04813 |
| EF1G_MOUSE | Q9D8N0 | Elongation factor 1-gamma | -1.64 | -4.06 | 2.42 | 0.04919 |
| ATP5I_MOUSE | Q06185 | ATP synthase subunit e mitochondrial | 3.72 | 1.61 | 2.12 | 0.04928 |
| AUHM_MOUSE | Q9JLZ3 | Methylglutaconyl-CoA hydratase mitochondrial | -1.52 | -4.61 | 3.09 | 0.04929 |
| EIF3A_MOUSE | P23116 | Eukaryotic translation initiation factor 3 subunit A | -0.63 | -3.50 | 2.87 | 0.04934 |
| LAP2B_MOUSE | Q61029 | Lamina-associated polypeptide 2 isoforms beta/delta/epsilon/gamma | -2.37 | -3.84 | 1.48 | 0.04988 |
