# Supplementary material for: First comprehensive identification of cardiac proteins with putative increased O-GlcNAc levels during pressure overload hypertrophy
Source: PLoS One. 2022 Oct 26;17(10):e0276285. doi: 10.1371/journal.pone.0276285 (PMC9605332; doi:10.1371/journal.pone.0276285)
Supplement: S3 Table — Log2 fold change of POH-Sham is shown for both global and O-GlcNAc protein levels. (DOCX) [file pone.0276285.s003.docx]

**S3 Table. Proteins with significantly increased global and putative O-GlcNAc levels during pressure overload hypertrophy (POH).** Log_2_ fold change of POH-Sham is shown for both global and O-GlcNAc.

| **Accession** | **Uniprot accession number** | **Description** | **Global: log2 Fold change POH-Sham** | **O-GlcNAc: log2 Fold change POH-Sham** | **Global: p-value** | **O-GlcNAc: p-value** |
| --- | --- | --- | --- | --- | --- | --- |
| TBA4A_MOUSE | P68368 | Tubulin alpha-4A chain | 0.27 | 2.15 | 0.0013 | 0.0065 |
| NEXN_MOUSE | Q7TPW1 | Nexilin | 0.53 | 2.06 | 0.0001 | 0.0037 |
| PAK2_MOUSE | Q8CIN4 | Serine/threonine-protein kinase PAK 2 | 1.26 | 2.02 | 0.0073 | 0.0020 |
| NDUS2_MOUSE | Q91WD5 | NADH dehydrogenase [ubiquinone] iron-sulfur protein 2 mitochondrial | 0.24 | 1.93 | 0.0275 | 0.0043 |
| RL12_MOUSE | P35979 | 60S ribosomal protein L12 | 0.43 | 3.56 | 0.0131 | 0.0167 |
| PDLI1_MOUSE | O70400 | PDZ and LIM domain protein 1 | 0.51 | 2.09 | 0.0101 | 0.0126 |
| B2MG_MOUSE | P01887 | Beta-2-microglobulin | 1.76 | 2.34 | 0.0399 | 0.0137 |
| MYH7_MOUSE | Q91Z83 | Myosin-7 | 1.05 | 2.70 | 0.0001 | 0.0227 |
| RS12_MOUSE | P63323 | 40S ribosomal protein S12 | 0.74 | 1.92 | 0.0130 | 0.0266 |
| RL13A_MOUSE | P19253 | 60S ribosomal protein L13a | 2.16 | 2.68 | 0.0262 | 0.0210 |
| CLIP1_MOUSE | Q922J3 | CAP-Gly domain-containing linker protein 1 | 0.98 | 3.45 | 0.0354 | 0.0182 |
| MACD1_MOUSE | Q922B1 | O-acetyl-ADP-ribose deacetylase MACROD1 | 0.44 | 3.79 | 0.0008 | 0.0227 |
| FLNC_MOUSE | Q8VHX6 | Filamin-C | 0.63 | 2.17 | 0.0083 | 0.0309 |
| NDUS8_MOUSE | Q8K3J1 | NADH dehydrogenase [ubiquinone] iron-sulfur protein 8 mitochondrial | 0.73 | 2.54 | 0.0104 | 0.0213 |
| EF1D_MOUSE | P57776 | Elongation factor 1-delta | 0.25 | 4.44 | 0.0385 | 0.0222 |
| NIPS2_MOUSE | O55126 | Protein NipSnap homolog 2 | 0.49 | 0.76 | 0.0165 | 0.0246 |
| HXK1_MOUSE | P17710 | Hexokinase-1 | 0.32 | 1.59 | 0.0363 | 0.0363 |
| CCHL_MOUSE | P53702 | Cytochrome c-type heme lyase | 2.46 | 2.06 | 0.0435 | 0.0286 |
| MYH9_MOUSE | Q8VDD5 | Myosin-9 | 0.64 | 2.39 | 0.0012 | 0.0373 |
| KINH_MOUSE | Q61768 | Kinesin-1 heavy chain | 1.24 | 2.41 | 0.0106 | 0.0421 |
| PDIA1_MOUSE | P09103 | Protein disulfide-isomerase | 0.35 | 2.39 | 0.0484 | 0.0448 |
| ANXA5_MOUSE | P48036 | Annexin A5 | 0.56 | 2.48 | 0.0302 | 0.0345 |
| RS2_MOUSE | P25444 | 40S ribosomal protein S2 | 0.71 | 2.19 | 0.0038 | 0.0375 |
| RS3_MOUSE | P62908 | 40S ribosomal protein S3 | 0.48 | 0.91 | 0.0112 | 0.0414 |
| ACADL_MOUSE | P51174 | Long-chain specific acyl-CoA dehydrogenase mitochondrial | 0.21 | 0.70 | 0.0081 | 0.0422 |
| RL4_MOUSE | Q9D8E6 | 60S ribosomal protein L4 | 0.54 | 2.90 | 0.0003 | 0.0475 |
