# Supplementary material for: First comprehensive identification of cardiac proteins with putative increased O-GlcNAc levels during pressure overload hypertrophy
Source: PLoS One. 2022 Oct 26;17(10):e0276285. doi: 10.1371/journal.pone.0276285 (PMC9605332; doi:10.1371/journal.pone.0276285)
Supplement: S4 Table — (DOCX) [file pone.0276285.s004.docx]

**S4 Table. Accessions for the putative proteins with increased O-GlcNAc levels during pressure-overload hypertrophy (POH) versus Sham for biological processes with significant overexpression from Table 1.**

| **Term** | **Count** | **Accessions** |
| --- | --- | --- |
| GO:0006412~translation | 24 | RL30_MOUSE, RT02_MOUSE, RL26_MOUSE, RS18_MOUSE, RL12_MOUSE, RS21_MOUSE, RL4_MOUSE, RS6_MOUSE, EF1G_MOUSE, EIF3A_MOUSE, RS12_MOUSE, RS3_MOUSE, CMC1_MOUSE, RS15_MOUSE, RRBP1_MOUSE, RS2_MOUSE, EIF3B_MOUSE, EF1D_MOUSE, RL13A_MOUSE, RS4X_MOUSE, IF5A1_MOUSE, RL3_MOUSE, RSSA_MOUSE, RS7_MOUSE |
| GO:0042407~cristae formation | 7 | AFG32_MOUSE, MIC27_MOUSE, MIC25_MOUSE, LETM1_MOUSE, MIC60_MOUSE, MIC19_MOUSE, MIC26_MOUSE |
| GO:0055114~oxidation-reduction process | 29 | PRDX6_MOUSE, NDUV2_MOUSE, NDUS2_MOUSE, NDUB3_MOUSE, QCR7_MOUSE, NDUB8_MOUSE, SPRE_MOUSE, GRHPR_MOUSE, NDUBA_MOUSE, DECR_MOUSE, ACADM_MOUSE, CYB5_MOUSE, SODM_MOUSE, NDUV1_MOUSE, L2HDH_MOUSE, GDIB_MOUSE, ACADS_MOUSE, DHB8_MOUSE, AIFM1_MOUSE, NDUS8_MOUSE, DLDH_MOUSE, NDUS5_MOUSE, NDUBB_MOUSE, MDHM_MOUSE, AK1A1_MOUSE, QCR9_MOUSE, COX2_MOUSE, ALDH2_MOUSE, ACADL_MOUSE |
| GO:0098609~cell-cell adhesion | 13 | NHRF2_MOUSE, 1433Z_MOUSE, PRDX6_MOUSE, VAPB_MOUSE, EF1G_MOUSE, ALDOA_MOUSE, BASI_MOUSE, LASP1_MOUSE, LAP2B_MOUSE, RS2_MOUSE, PDLI1_MOUSE, USO1_MOUSE, EF1D_MOUSE |
| GO:0006754~ATP biosynthetic process | 6 | NIPS2_MOUSE, ATP5I_MOUSE, ATP5L_MOUSE, ATPB_MOUSE, ALDOA_MOUSE, ATPK_MOUSE |
| GO:0046034~ATP metabolic process | 7 | KAD2_MOUSE, AT1A2_MOUSE, ATP5I_MOUSE, ATP5L_MOUSE, ATPB_MOUSE, MYH7_MOUSE, ATPK_MOUSE |
| GO:0006096~glycolytic process | 6 | PGAM2_MOUSE, PGAM1_MOUSE, PGK1_MOUSE, HXK1_MOUSE, ALDOA_MOUSE, G6PI_MOUSE |
| GO:0006810~transport | 42 | ACTN4_MOUSE, TOM1_MOUSE, NDUV2_MOUSE, NDUS2_MOUSE, NDUB3_MOUSE, QCR7_MOUSE, TRI72_MOUSE, DC1L1_MOUSE, CPT1B_MOUSE, NDUB8_MOUSE, LASP1_MOUSE, TM1L2_MOUSE, NDUBA_MOUSE, AT1A2_MOUSE, CYB5_MOUSE, G3BP2_MOUSE, APOE_MOUSE, DC1I2_MOUSE, NDUV1_MOUSE, ATPK_MOUSE, FUBP2_MOUSE, TRFE_MOUSE, ATP5I_MOUSE, DLRB1_MOUSE, NDUS8_MOUSE, TIM44_MOUSE, ALBU_MOUSE, CMC1_MOUSE, RRBP1_MOUSE, NDUS5_MOUSE, NDUBB_MOUSE, QCR9_MOUSE, COX2_MOUSE, ATP5L_MOUSE, USO1_MOUSE, ATPB_MOUSE, RAN_MOUSE, MTCH2_MOUSE, G3BP1_MOUSE, TTHY_MOUSE, IF5A1_MOUSE, CLIP1_MOUSE |
