# Supplementary material for: First comprehensive identification of cardiac proteins with putative increased O-GlcNAc levels during pressure overload hypertrophy
Source: PLoS One. 2022 Oct 26;17(10):e0276285. doi: 10.1371/journal.pone.0276285 (PMC9605332; doi:10.1371/journal.pone.0276285)
Supplement: S5 Table — (DOCX) [file pone.0276285.s005.docx]

**S5 Table. Accessions for the putative proteins with significantly increased O-GlcNAc levels during pressure-overload hypertrophy (POH) versus Sham for KEGG pathways with significant overexpression from Table 2.**

| **Term** | **Count** | **Accessions** |
| --- | --- | --- |
| mmu01200:Carbon metabolism | 17 | SUCB2_MOUSE, PCCA_MOUSE, ACADS_MOUSE, HXK1_MOUSE, ALDOA_MOUSE, DLDH_MOUSE, PGAM2_MOUSE, PGAM1_MOUSE, MDHM_MOUSE, TKT_MOUSE, PGK1_MOUSE, ACADM_MOUSE, ODP2_MOUSE, FUMH_MOUSE, THIL_MOUSE, AATC_MOUSE, G6PI_MOUSE |
| mmu00190:Oxidative phosphorylation | 18 | COX5A_MOUSE, NDUV2_MOUSE, NDUS2_MOUSE, NDUB3_MOUSE, QCR7_MOUSE, ATP5I_MOUSE, NDUB8_MOUSE, NDUS8_MOUSE, NDUBA_MOUSE, NDUS5_MOUSE, NDUBB_MOUSE, QCR9_MOUSE, COX2_MOUSE, ATP5L_MOUSE, ATPB_MOUSE, COX5B_MOUSE, NDUV1_MOUSE, ATPK_MOUSE |
| mmu01130:Biosynthesis of antibiotics | 20 | KAD2_MOUSE, SUCB2_MOUSE, PCCA_MOUSE, HXK1_MOUSE, ALDOA_MOUSE, DLDH_MOUSE, PGAM2_MOUSE, PGAM1_MOUSE, MDHM_MOUSE, TKT_MOUSE, PGK1_MOUSE, ACADM_MOUSE, ODP2_MOUSE, AK1A1_MOUSE, FUMH_MOUSE, PGM1_MOUSE, THIL_MOUSE, AATC_MOUSE, ALDH2_MOUSE, G6PI_MOUSE |
| mmu05012:Parkinson's disease | 17 | UBA1_MOUSE, COX5A_MOUSE, UB2L3_MOUSE, NDUV2_MOUSE, NDUS2_MOUSE, NDUB3_MOUSE, QCR7_MOUSE, NDUB8_MOUSE, NDUS8_MOUSE, NDUBA_MOUSE, NDUS5_MOUSE, NDUBB_MOUSE, QCR9_MOUSE, COX2_MOUSE, ATPB_MOUSE, COX5B_MOUSE, NDUV1_MOUSE |
| mmu05016:Huntington's disease | 19 | DCTN2_MOUSE, COX5A_MOUSE, NDUV2_MOUSE, NDUS2_MOUSE, NDUB3_MOUSE, QCR7_MOUSE, NDUB8_MOUSE, NDUS8_MOUSE, NDUBA_MOUSE, NDUS5_MOUSE, NDUBB_MOUSE, CLH1_MOUSE, QCR9_MOUSE, SODM_MOUSE, COX2_MOUSE, ATPB_MOUSE, TGM2_MOUSE, COX5B_MOUSE, NDUV1_MOUSE |
| mmu03010:Ribosome | 16 | RL30_MOUSE, RT02_MOUSE, RL26_MOUSE, RS18_MOUSE, RL12_MOUSE, RS21_MOUSE, RL4_MOUSE, RS12_MOUSE, RS3_MOUSE, RS15_MOUSE, RS2_MOUSE, RL13A_MOUSE, RS4X_MOUSE, RL3_MOUSE, RSSA_MOUSE, RS7_MOUSE |
| mmu05010:Alzheimer's disease | 17 | COX5A_MOUSE, NDUV2_MOUSE, NDUS2_MOUSE, NDUB3_MOUSE, QCR7_MOUSE, NDUB8_MOUSE, NDUS8_MOUSE, NDUBA_MOUSE, NDUS5_MOUSE, NDUBB_MOUSE, QCR9_MOUSE, COX2_MOUSE, ATPB_MOUSE, APOE_MOUSE, COX5B_MOUSE, NDUV1_MOUSE, TAU_MOUSE |
| mmu00010:Glycolysis / Gluconeogenesis | 11 | DLDH_MOUSE, PGAM2_MOUSE, PGAM1_MOUSE, PGK1_MOUSE, ODP2_MOUSE, AK1A1_MOUSE, PGM1_MOUSE, HXK1_MOUSE, ALDOA_MOUSE, ALDH2_MOUSE, G6PI_MOUSE |
| mmu01100:Metabolic pathways | 47 | COX5A_MOUSE, PRDX6_MOUSE, NDUV2_MOUSE, NDUS2_MOUSE, NDUB3_MOUSE, QCR7_MOUSE, NDUB8_MOUSE, SPRE_MOUSE, THTM_MOUSE, GRHPR_MOUSE, AUHM_MOUSE, NDUBA_MOUSE, PGAM2_MOUSE, ODP2_MOUSE, ACADM_MOUSE, COX5B_MOUSE, NDUV1_MOUSE, ATPK_MOUSE, G6PI_MOUSE, KAD2_MOUSE, SUCB2_MOUSE, ATP5I_MOUSE, ACADS_MOUSE, PCCA_MOUSE, DHB8_MOUSE, HXK1_MOUSE, ALDOA_MOUSE, NDUS8_MOUSE, DLDH_MOUSE, NDUS5_MOUSE, PGAM1_MOUSE, NDUBB_MOUSE, PGK1_MOUSE, MDHM_MOUSE, TKT_MOUSE, B4GT1_MOUSE, AK1A1_MOUSE, QCR9_MOUSE, FUMH_MOUSE, COX2_MOUSE, PGM1_MOUSE, ATP5L_MOUSE, ATPB_MOUSE, THIL_MOUSE, AATC_MOUSE, ALDH2_MOUSE, ACADL_MOUSE |
| mmu04932:Non-alcoholic fatty liver disease (NAFLD) | 14 | COX5A_MOUSE, NDUV2_MOUSE, NDUS2_MOUSE, NDUB3_MOUSE, QCR7_MOUSE, NDUB8_MOUSE, NDUS8_MOUSE, NDUBA_MOUSE, NDUS5_MOUSE, NDUBB_MOUSE, QCR9_MOUSE, COX2_MOUSE, COX5B_MOUSE, NDUV1_MOUSE |
| mmu00620:Pyruvate metabolism | 7 | DLDH_MOUSE, MDHM_MOUSE, ODP2_MOUSE, FUMH_MOUSE, THIL_MOUSE, GRHPR_MOUSE, ALDH2_MOUSE |
| mmu00280:Valine, leucine and isoleucine degradation | 7 | DLDH_MOUSE, ACADM_MOUSE, ACADS_MOUSE, PCCA_MOUSE, THIL_MOUSE, ALDH2_MOUSE, AUHM_MOUSE |
| mmu00630:Glyoxylate and dicarboxylate metabolism | 5 | DLDH_MOUSE, MDHM_MOUSE, PCCA_MOUSE, THIL_MOUSE, GRHPR_MOUSE |
| mmu00071:Fatty acid degradation | 6 | ACADM_MOUSE, CPT1B_MOUSE, ACADS_MOUSE, THIL_MOUSE, ALDH2_MOUSE, ACADL_MOUSE |
| mmu00020:Citrate cycle (TCA cycle) | 5 | DLDH_MOUSE, SUCB2_MOUSE, MDHM_MOUSE, ODP2_MOUSE, FUMH_MOUSE |
| mmu04260:Cardiac muscle contraction | 7 | COX5A_MOUSE, AT1A2_MOUSE, QCR7_MOUSE, QCR9_MOUSE, COX2_MOUSE, MYH7_MOUSE, COX5B_MOUSE |
