# Supplementary material for: First comprehensive identification of cardiac proteins with putative increased O-GlcNAc levels during pressure overload hypertrophy
Source: PLoS One. 2022 Oct 26;17(10):e0276285. doi: 10.1371/journal.pone.0276285 (PMC9605332; doi:10.1371/journal.pone.0276285)
Supplement: S1 Raw images — (PDF) [file pone.0276285.s006.pdf]

Figure 2

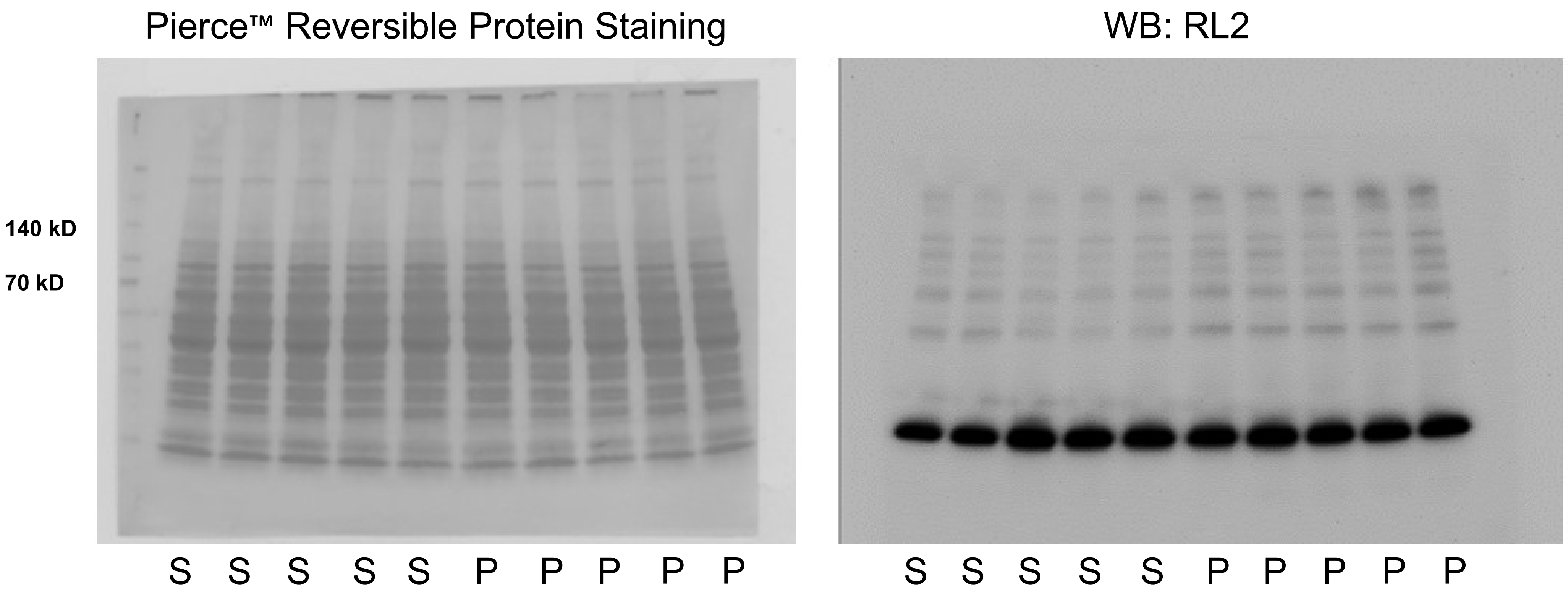

Legend: S=Sham; P=POH

Figure 4A

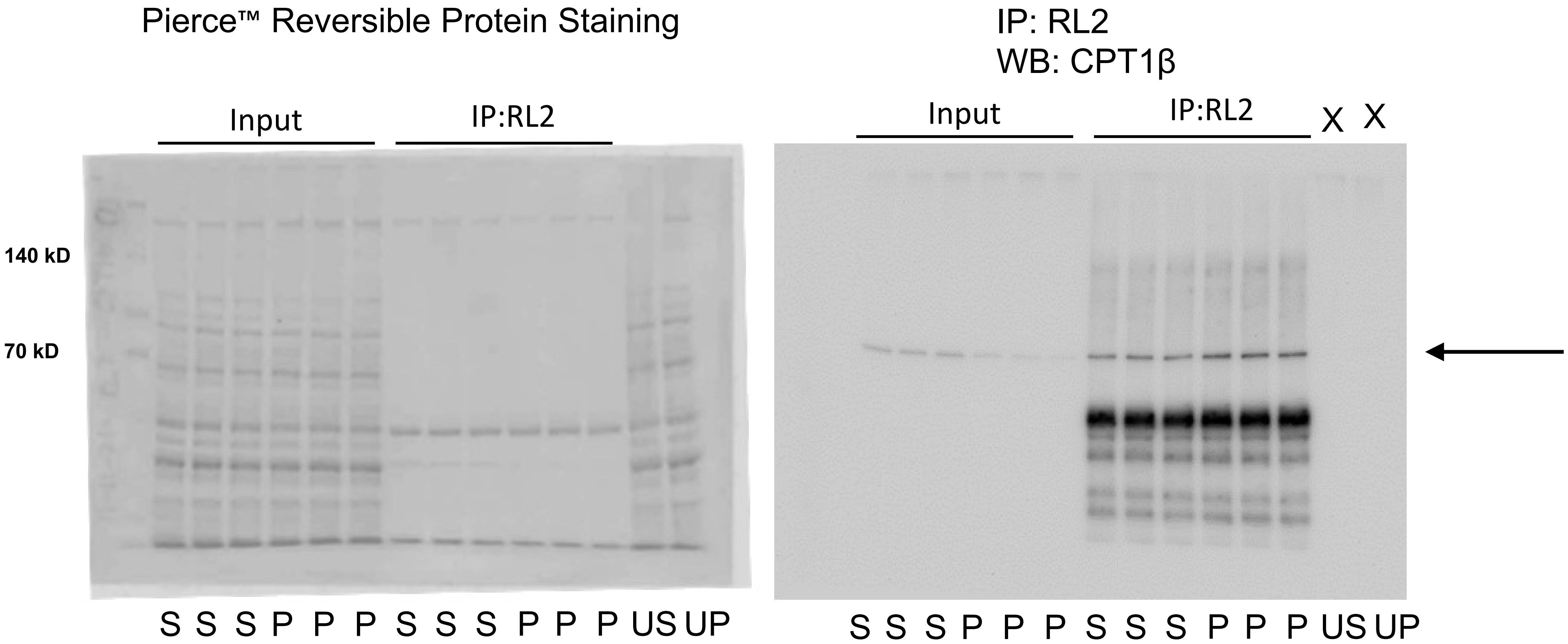

Legend: S=Sham; P=POH, Unbound-Sham = US, Unbound-POH=UP

Figure 4D

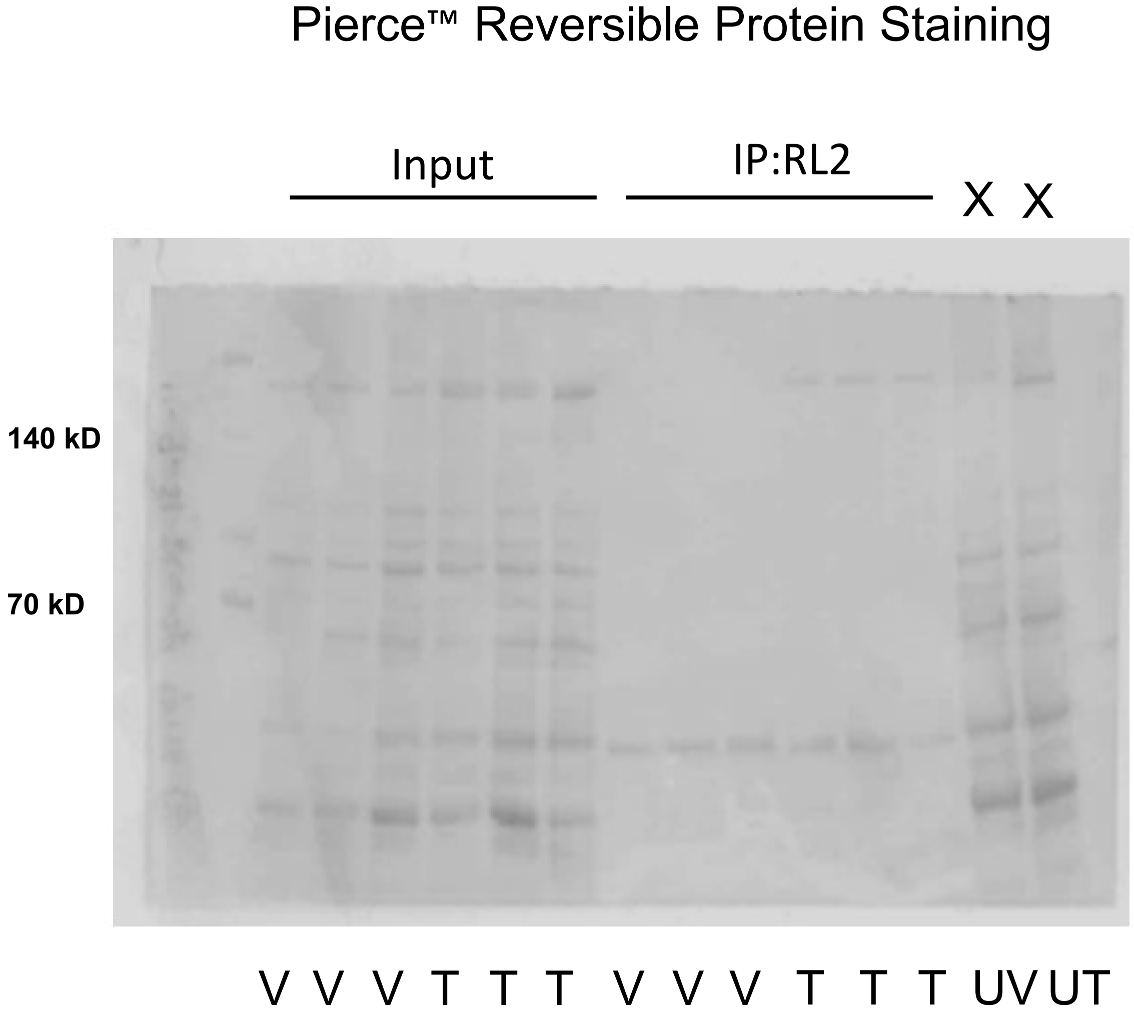

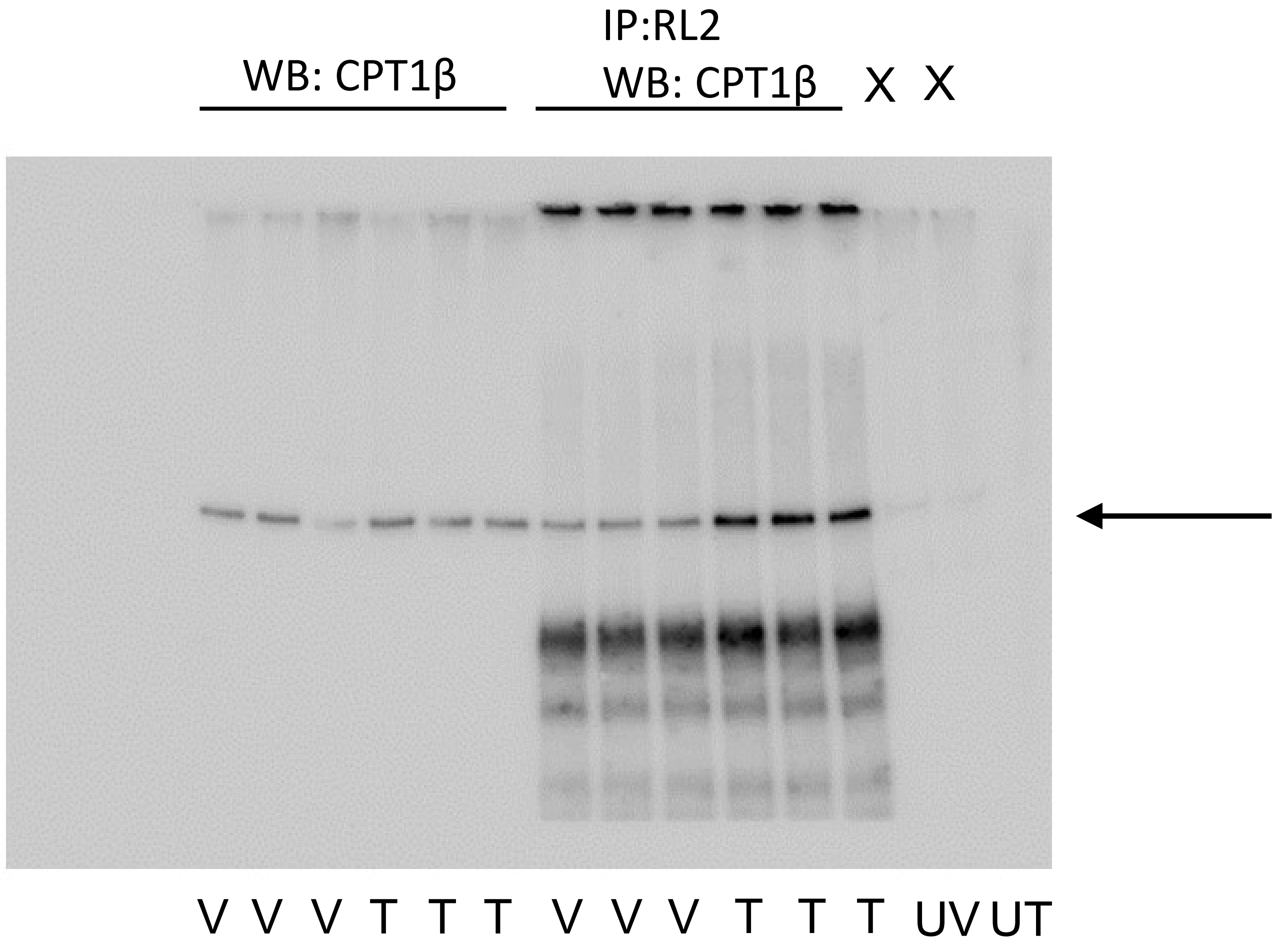

Legend: V=V-TAC; T=TMG-TAC; UV=unbound V-TAC; UT=unbound TMG-TAC

Figure 6A

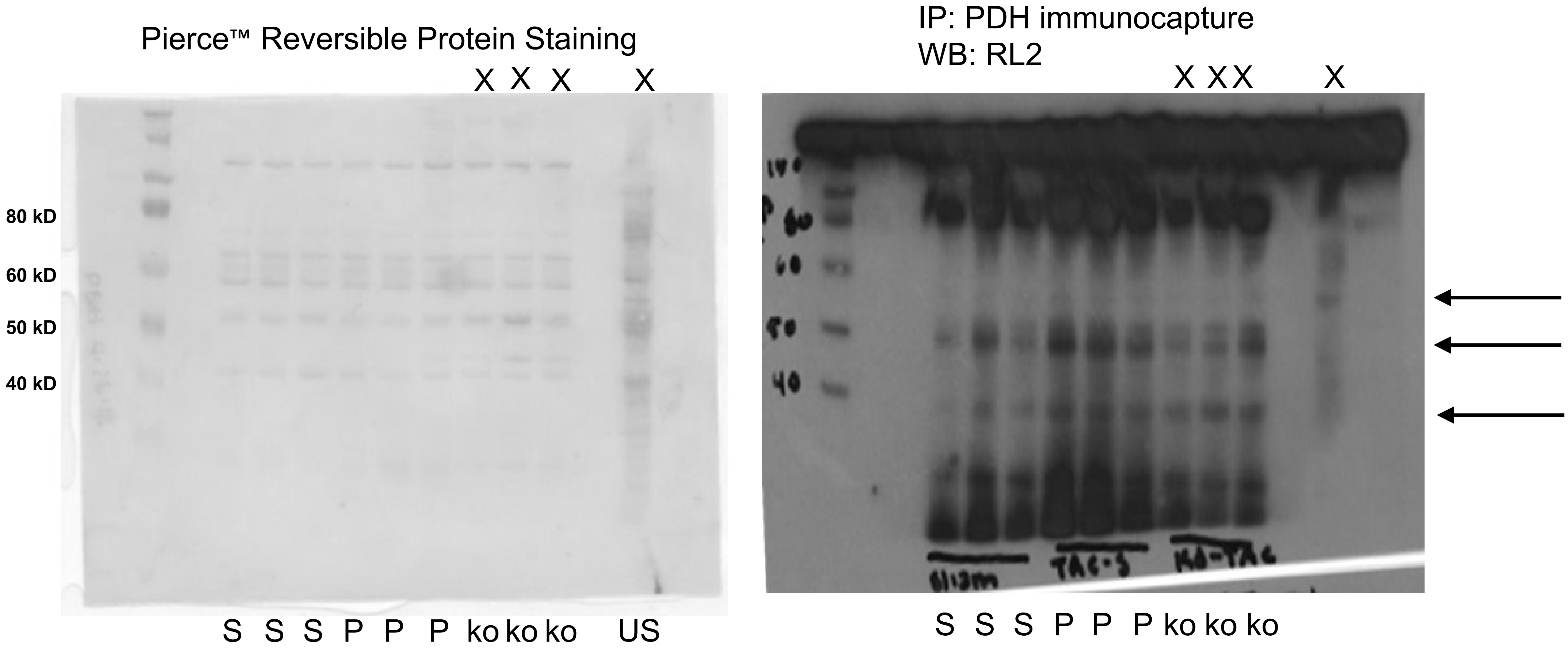

Legend: S=Sham; P=POH, Unbound-Sham = US. KO=O-GlcNAC Transferase (OGT) knock-out TAC which were samples from a previous study.

Figure 6D

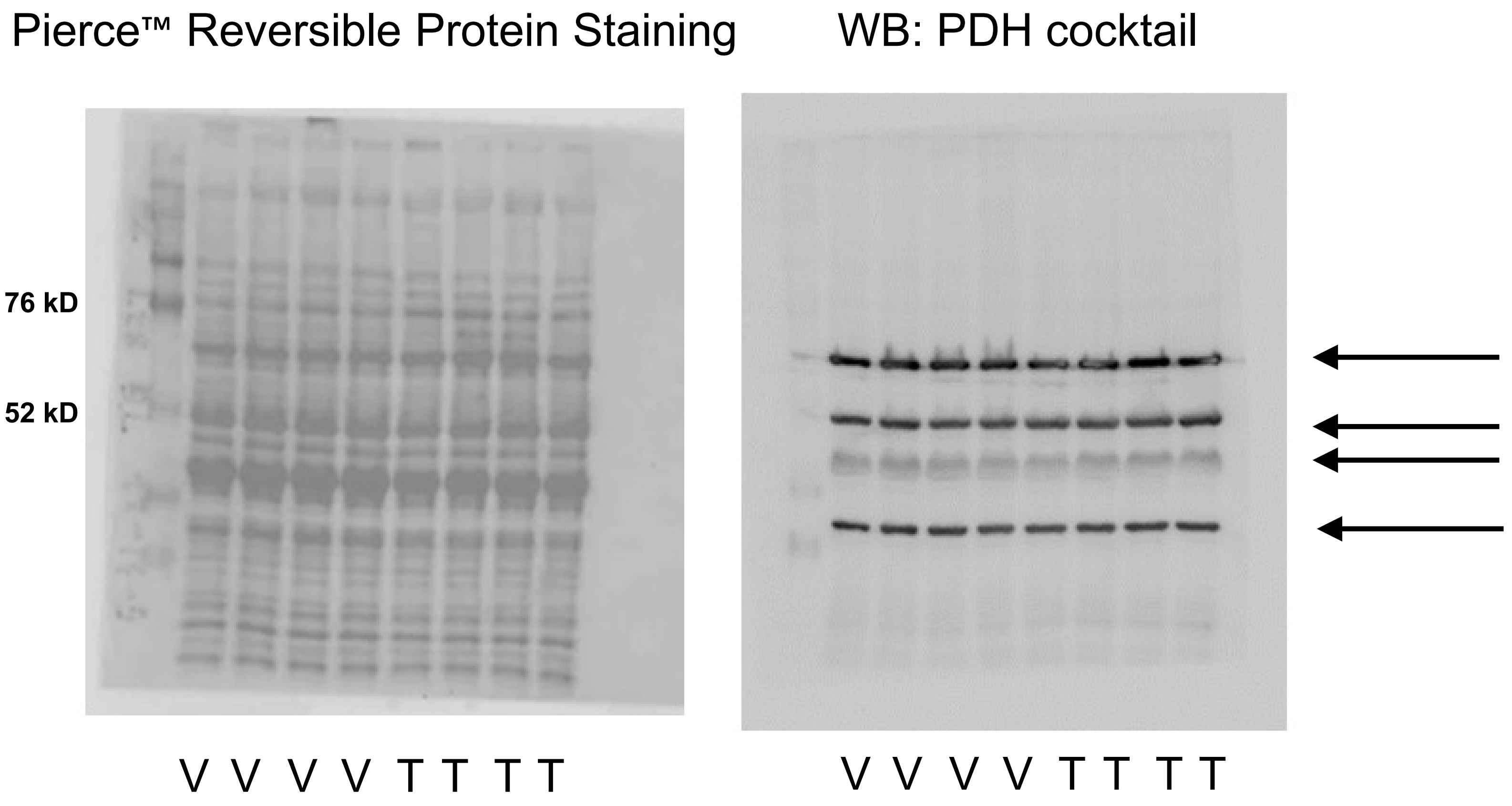

Legend: V=V-TAC; T=TMG-TAC

Figure 6H

Pierce™ Reversible Protein Staining

IP: RL2

76 kD

52 kD

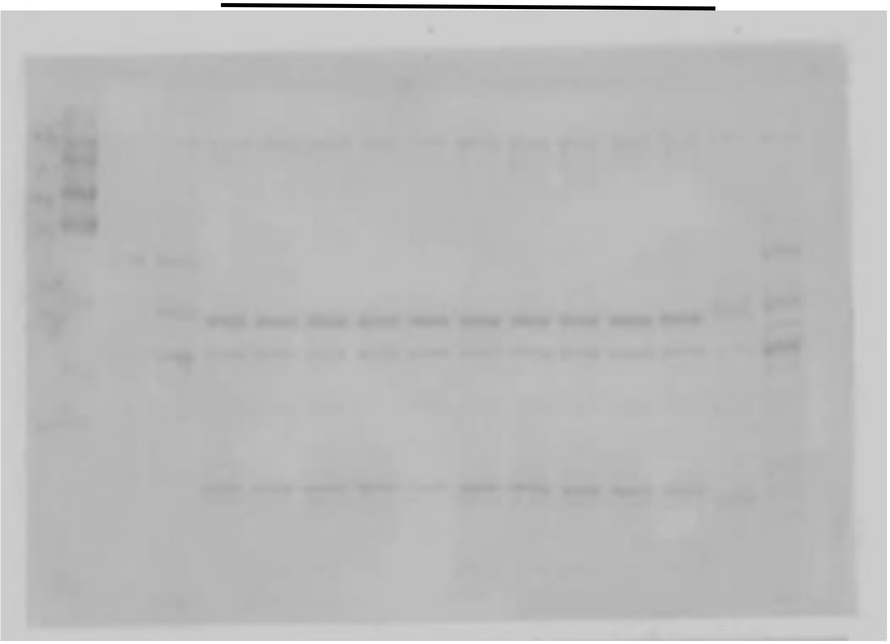

IV IT V V V V V T T T T T IgG UV

IP: RL2
WB: PDH Cocktail

X X X X

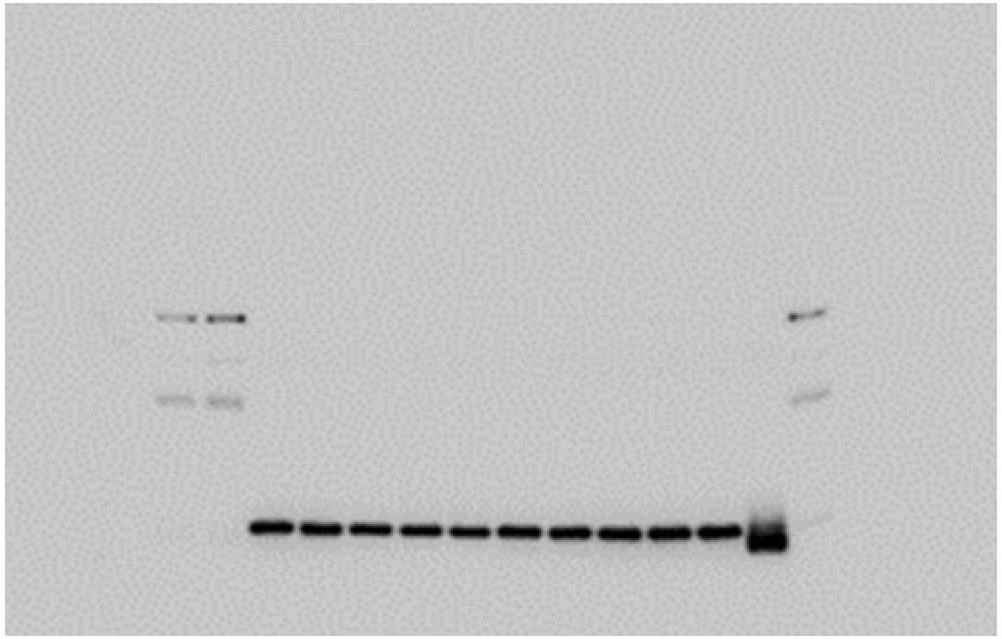

Note, the prominent band at about 22 kD inhibited detection of the PDH subunits by WB post-IP, so the image used for quantification was captured by excluding that band and focusing on molecular weight region of interest.

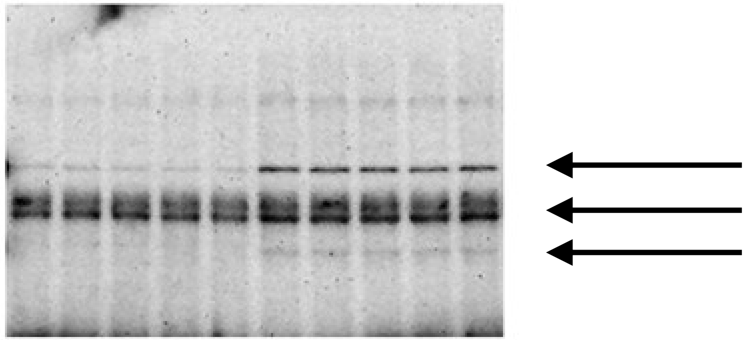

V V V V V T T T T T

Legend: V=V-TAC; T=TMG-TAC; IV: input V-TAC; IT: input TMG-TAC; UV=unbound V-TAC, IgG=IgG added to V-TAC
